# Phylogenomic Analyses of *Bradyrhizobium* Reveal Uneven Distribution of the Lateral and Subpolar Flagellar Systems, Which Extends to *Rhizobiales*

**DOI:** 10.3390/microorganisms7020050

**Published:** 2019-02-13

**Authors:** Daniel Garrido-Sanz, Miguel Redondo-Nieto, Elías Mongiardini, Esther Blanco-Romero, David Durán, Juan I. Quelas, Marta Martin, Rafael Rivilla, Aníbal R. Lodeiro, M. Julia Althabegoiti

**Affiliations:** 1 Departamento de Biología, Facultad de Ciencias, Universidad Autónoma de Madrid, c/Darwin 2, 28049 Madrid, Spain; daniel.garrido@uam.es (D.G.-S.); miguel.redondo@uam.es (M.R.-N.); esther.blanco@uam.es (E.B.-R.); david.duran@uam.es (D.D.); m.martin@uam.es (M.M.); rafael.rivilla@uam.es (R.R.); 2 Instituto de Biotecnología y Biología Molecular (IBBM), Facultad de Ciencias Exactas, UNLP y CCT-La Plata-CONICET, La Plata B1900, Argentina; mongiardini@biol.unlp.edu.ar (E.M.); quelas@biol.unlp.edu.ar (J.I.Q.); lodeiro@biol.unlp.edu.ar (A.R.L.)

**Keywords:** *Bradyrhizobium*, *Rhizobiales*, phylogenomic, phylogenetic, flagellar systems

## Abstract

Dual flagellar systems have been described in several bacterial genera, but the extent of their prevalence has not been fully explored. *Bradyrhizobium diazoefficiens* USDA 110^T^ possesses two flagellar systems, the subpolar and the lateral flagella. The lateral flagellum of *Bradyrhizobium* displays no obvious role, since its performance is explained by cooperation with the subpolar flagellum. In contrast, the lateral flagellum is the only type of flagella present in the related *Rhizobiaceae* family. In this work, we have analyzed the phylogeny of the *Bradyrhizobium* genus by means of Genome-to-Genome Blast Distance Phylogeny (GBDP) and Average Nucleotide Identity (ANI) comparisons of 128 genomes and divided it into 13 phylogenomic groups. While all the *Bradyrhizobium* genomes encode the subpolar flagellum, none of them encodes only the lateral flagellum. The simultaneous presence of both flagella is exclusive of the *B. japonicum* phylogenomic group. Additionally, 292 *Rhizobiales* order genomes were analyzed and both flagellar systems are present together in only nine genera. Phylogenetic analysis of 150 representative *Rhizobiales* genomes revealed an uneven distribution of these flagellar systems. While genomes within and close to the *Rhizobiaceae* family only possess the lateral flagellum, the subpolar flagellum is exclusive of more early-diverging families, where certain genera also present both flagella.

## 1. Introduction

Many of the diverse lineages that integrate the *Bacteria* domain have a free-living planktonic state as an important part of their lifestyles. Furthermore, in planktonic state, bacteria synthesize extracellular structures known as flagella that are used for chemotactic motility. Flagellar systems are widely distributed among bacteria, conferring significant adaptive advantages [1]. Flagella are composed of a basal body that anchors the flagellar structure to the cell surface, a long filament protruding from the cell body, and a hook that serves as a connector between the basal body and the filament. The basal body contains the flagellar motor, consisting of a stator (MotAB or PomAB, according to the motive force) anchored to the cytoplasmic membrane and peptidoglycan, and a rotor that moves the extracellular substructure. This rotor is composed of the MS-ring (FliF), the C-ring (FliGMN) and the rod (FlgBCFG). FlgH and FlgI also belong to the rod and form the bushings, called the L and P rings that are found in the outer membrane and in the peptidoglycan layer, respectively. The bushings surround the drive shaft and keep it in place. The hook (FlgE) connects the filament to the basal body and FlgL and FlgK establish the hook-filament junction. The filament that projects out of the cell, often reaching up to 10–20 times the cell’s length, is composed of thousands of flagellin monomers (FliC, Fla or LafA) [2,3]. All the components of this structure are synthesized inside the cell and therefore must be secreted through the export apparatus (FlhAB and FliIPRQ). Furthermore, the above-described flagellar core genes belong to a complex system that requires around 50 proteins for their assemblage, regulation, and function, being an expensive organelle from the energetic standpoint [4,5]. Indeed, a substantial amount of protein synthesis is required to maintain the flagellar system throughout the cell cycle, and considerable energy is dedicated to motor rotation.

Position, number, and identity of the flagellar filaments around the cell differ among bacterial species. Flagellar filaments can be subpolar or polar if positioned near or at the cell pole, respectively; or peritrichous when they are located around the cell [6]. Moreover, different genetic arrangements occur in flagellar systems, since all the genes can be located in a single cluster or they can be scattered in different clusters along the genome. Several works indicate that a number of species of diverse genera present two sets of flagellar genes, therefore they putatively encode dual flagellar systems [1,7,8,9,10]. Among aquatic bacteria, dual flagellar systems have been reported in strains or species of *Vibrio*, *Aeromonas*, *Rhodospirillum*, *Rhodobacter* and *Shewanella* [7,8,9,11,12]. Microorganisms from other environments, such as strains of the gut inhabitant *Escherichia coli* [13] or strains of certain species of the soil bacteria *Azospirillum*, *Bradyrhizobium*, and *Pseudomonas* are also described as possessing two flagellar systems [10,14,15]. An irregular distribution of flagella is frequently found among different species or strains [13,15,16,17,18]. Considering the energetic demands for flagellar synthesis and functioning, the existence of dual flagellar systems in these bacteria is noteworthy and requires deeper analysis of their evolution and adaptive value.

*Bradyrhizobium diazoefficiens* USDA 110^T^ possesses two flagellar systems [10,19,20]. The genes for the subpolar flagellum are distributed in four major clusters and several orphan genes along the chromosome, while all genes encoding the lateral flagellum are located in a 35-kb cluster [21]. However, a preliminary survey gave no indications of the possible acquisition of this cluster by Horizontal Gene Transfer (HGT) [22]. Previous works demonstrate that the flagellar systems of *B. diazoefficiens* USDA 110^T^ have distinctive characteristics. The subpolar flagellum, which is constitutively expressed, is required to swim in liquid and to swarm over wet surfaces [23]. In addition, it is chemotactically active and behaves as an adhesin on glass surfaces. The lateral flagella are expressed in liquid medium, consuming a considerable portion of cellular energy and being induced by the carbon source and the presence of agar or the viscosity of the medium [22,24]. It does not contribute to swimming speed, but is partially responsible for swarming [22,23]. Mutants lacking the subpolar flagellum are impaired in colonization of soybean roots or in competition for nodulation, while the role of the lateral flagellum on this bacteria-plant interaction remains unclear [19]. In nature, some *Bradyrhizobium* species such as *B. elkanii* possess only the subpolar flagellum, yet they live in the same soil environment and nodulate the same plant host as *B. diazoefficiens* or *B. japonicum*, which possess both flagellar systems [22,25]. These facts add more puzzlement to the question of the adaptive value of lateral flagella in this genus, which has been found as the dominant and most ubiquitous in soils worldwide [26,27].

To date, few studies have focused on analyzing the distribution of flagellar systems in bacteria [1,9]. To shed more light on these questions regarding dual flagellar systems, here we studied the phylogenetic distribution of the lateral and subpolar flagella and analyzed their synteny throughout *Bradyrhizobium* and the *Rhizobiales* order of *alpha-proteobacteria*.

## 2. Materials and Methods

### 2.1. Datasets and Phylogenomic Analysis of Bradyrhizobium

*Bradyrhizobium* genomes were downloaded from the NCBI ftp server [28] and the JGI IMG database [29] on October 2017, resulting in a total of 128 genomes listed in the Supplementary Table S1. Phylogenomic analyses were conducted by (i) calculating intergenomic distances using the Genome-to-Genome Blast Distance Phylogeny (GBDP) algorithm via the Genome-to-Genome Distance Calculator (GGDC) 2.1 web service [30,31] and (ii) by using blast-implemented Average Nucleotide Identity (ANIb) [32] using a public available script [33]. Resulting sets of intergenomic distances obtained by GBDP and distances based on ANIb as previously described [34,35] (Supplementary Data S1) were converted into distance matrices and imported into MEGA 7 software [36] to build the Neighbor-Joining phylogenomic trees. *Rhizobium lupini* HPC(L) and *Mesorhizobium ciceri* WSM1271 genomes were used as outgroups.

Clustering of GBDP and ANIb intergenomic distances from the *Bradyrhizobium* genus into phylogenomic groups was examined with OPTSIL clustering software version 1.5 [37]. Here, and also as previously proposed [34,38], an *F* = 0.5 (i.e., average-linkage clustering) was chosen and *T* values were examined to detect the best clustering threshold based on reference partitions that yielded the highest Modified Rand Index (MRI) values. Species-level clusters were also examined using the established thresholds for both, GBDP and ANIb analyses.

### 2.2. Phylogenetic Analysis of Rhizobiales Order Genomes

All-against-all orthologous amino acid searches with DIAMOND version 0.9.22.123 [39] implementation were performed using OrthoFinder version 2.2.6 [40] on 150 representative proteomes belonging to the *Rhizobiales* order of *alpha-proteobacteria* (Supplementary Table S1) and *Caulobacter vibrioides* CB15 as outgroup. The resulting set of 104-core, single copy orthologous amino acid sequences was aligned using Clustal Omega [41] and concatenated to form the super-matrix for phylogenetic analysis. Gblocks version 0.91b [42] was used to remove poorly aligned columns and divergent regions, using a minimum block length of two amino acids and allowing gap positions in all sequences. Maximum likelihood (ML) phylogenetic trees were built using the Pthreads-parallelized RAxML package [43] version 8.2.10. The LG model of amino acid evolution [44] combined with gamma-distributed substitution rates and empirical amino acid frequencies were used. Fast bootstrapping with subsequent search for the best tree [45] and the autoMRE criterion [46] were also applied. Maximum parsimony (MP) tree searches were performed with PAUP* software [47] version 4.0a, using 100 rounds of random sequence addition and subsequent TBR branch swapping, saving no more than 10 best trees per round and collapsing potential zero-length branches during the tree search. MP bootstrap support was calculated with PAUP* using 1,000 replicates with 10 rounds of heuristic search per replicate.

### 2.3. Phylogenetic Analysis of the Flagellar Systems

Phylogenetic multilocus sequence analysis (MLSA) of the lateral flagellum in the *Bradyrhizobium* genus was performed using 35 conserved and complete coding DNA sequences (CDSs; *lafR*, bll6847, *flgN*, *flgJ*, *fliR*, *flhA*, *fliQ*, *flgD*, *flbT*, *flaF*, *flgL*, *flgK*, *flgE*, *fliK*, *motB*, bll6863, *fliF*, *fliL*, *flgH*, bll6870, *flgI*, *flgA*, *flgG*, *fliE*, *flgC, flgB*, *flhB*, *fliG*, *fliN*, bll6880, *fliM*, *motA*, *flgF1*, *fliI* and bll6886 as in *B. diazoefficiens* USDA 110^T^ genome BA000040.2 from position 7,544,342 to 7,577,700). Homologous sequences of these CDSs were retrieved from the genomic annotation, if available or by blast searches, aligned, processed and concatenated as described above, following the order in which they appear in *B. diazoefficiens* USDA 110^T^. The ML phylogenetic tree was built with RAxML as previously specified but using the GTR model of nucleotide substitution [48] combined with the gamma model of rate heterogeneity and optimization of substitution rates with the BFGS algorithm optimization. Rapid bootstrapping and subsequent ML search combined with autoRME criterium were used as specified above.

MLSA of both, the subpolar and lateral flagellar systems present in the genomes of the *Rhizobiales* order, was performed with 13 orthologous conserved amino acid sequences between these flagella (FliR, FlhA, FliQ, FliF, FliP, FlgH, FlgI, FlgG, FliE, FlgC, FlgB, FlhB, FliG) identified with OrthoFinder as described above. Genomes without this complete protein set or duplications were removed from the analysis. Concatenation of sequences, alignment and ML and MP tree searches were conducted as previously described.

### 2.4. Lateral and Subpolar Flagellar Systems Identification and Synteny

Genome annotations from all the genomes used in this study were obtained from the NCBI RefSeq database when possible; a RAST annotation pipeline [49] was used. Lateral and subpolar flagellar apparatus were screened in each of these genomes by means of genomic annotation searches and blastn and blastp from the blast+ software version 2.2.28 [50], using as reference the *B. diazoefficiens* USDA 110^T^ genomic sequence (BA000040.2) from position 7,545,799 to 7,552,767 containing the genes *fliR*, *flhA*, *fliQ*, *flgD*, *flbT*, *flaF*, *flgL* and *flgK* of the lateral flagellar apparatus, and from positions 6,375,374 to 6,380,905 containing the genes *flhB*, *fliR*, *fliQ*, *fliE*, *flgC*, *flgB*, *fliO* and *fliP* of the subpolar flagellum, which are distinctive of both flagella across all genomes analyzed. In the case of phylogenetically distant genomes, genomic sequences of both flagellar systems identified in close related genomes were used as blast queries.

Syntenic organization of the lateral flagellar system was examined using the genomic annotations in all the genomes analyzed and represented by a self-written Perl script.

## 3. Results and Discussion

### 3.1. Phylogenomic Analysis of the Bradyrhizobium Genus

**Figure 1:**
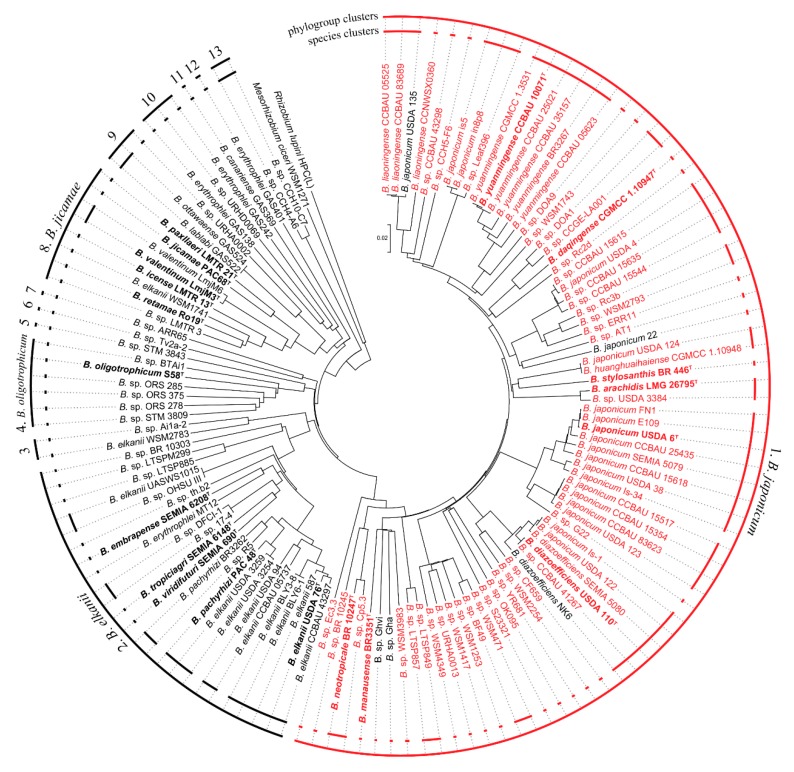
Phylogenomic tree based on GBDP intergenomic distances of 128 *Bradyrhizobium* genomes and clusters at phylogenetic groups (PGs) level and species level (SPs). *Rhizobium lupini* HPC(L) and *Mesorhizobium ciceri* WSM1271 were used as outgroups. Only genomes clustered within the PG1 (*B. japonicum*) harbor both, the lateral and subpolar flagellar systems (red typing), while genomes of the remaining PGs (2 to 13) and some within the PG1 only harbor the subpolar flagellar system (black typing). Bold and ^T^ indicate type strain. Phylogroups and species clusters according to GBDP intergenomic distance-based clustering.

The *Bradyrhizobium* genus has been described as a taxonomically complex group due to its high 16S rRNA gene conservation [51,52], although MLSA studies suggested the existence of two major groups within this genus [53,54]. Nowadays, the number of sequenced genomes allows the use of phylogenomics to achieve a deeper knowledge of *Bradyrhizobium* lineages. Phylogenomic GBDP and ANIb-based analysis of 128 *Bradyrhizobium* genomes and further clustering of all *Bradyrhizobium* intergenomic distances (Supplementary Data S1) with OTPSIL revealed the presence of 13 groups within the genus (Figure 1, Supplementary Figure S1). These 13 phylogenomic groups (PG) are in full agreement according to the Modified Rand Index (MRI = 1) with the reference partition using a distance threshold *T* = 0.153 within the GBDP distances, which equals 28.1% digital DNA-DNA hybridization (dDDH). The same 13 PGs with a MRI = 0.993 were obtained by clustering intergenomic distances calculated with ANIb using *T* = 0.15 (Supplementary Data S2), which equals an ANIb of 85%. The fact that both phylogenomic methods yielded similar clustering results, highlights the robustness of the PGs identified in this study. Furthermore, these distance thresholds (0.153 and 0.15 for GBDP and ANIb, respectively) are similar to those reported in a phylogroup identification study in *Pseudomonas* [34]. Of these 13 *Bradyrhizobium* PGs, only four contain sequenced type-strains, and according to the oldest species description: *B. japonicum* (PG 1), *B. elkanii* (PG 2), *B. oligotrophicum* (PG 4) and *B. jicamae* (PG 8) (Figure 1). The PGs presented here are in agreement with previous 16S rRNA PCR-based analysis along with MLSA of housekeeping genes of the *Bradyrhizobium* genus, which showed two major groups [54,55,56,57], namely Group I (GI) and Group II (GII). On the one hand, GI is consistent with our PG1, both including the species *B. japonicum*, *B. diazoefficiens*, *B. liaoningense*, *B. yuanmingense*, *B. daqigense*, *B. stylosanthis*, *B. arachidis*, *B. manausense* and *B. neotropicale*. On the other hand, GII includes more early-diverging species, such as *B. elkanii*. *B. pachyrhizi*, *B. embrapense*, *B. tropiciagri*, *B. viridifuturi*, *B. retamae*, *B. icense*, *B. valentinum*, *B. paxllaeri* and *B. jicamae*, all of which correspond with PGs 2 to 13 according to our results. The only discrepancy between both analyses lies in *B. oligotrophicum*, which is placed within the GI according to previous analysis [57], while in our study, it is an independent group.

Furthermore, when using the standard species thresholds of dDDH and ANI [30,32], there are 81 species clusters according to 70% dDDH and 77 species clusters according to an ANIb of 96% within the 128 *Bradyrhizobium* genomes analyzed (Figure 1, Supplementary Figure S1 and Data S2). In the case of GBDP, total correlation with the reference partition (i.e., MRI = 1) was achieved, while ANIb yielded a less optimal clustering result (MRI = 0.965). The 81 species-level clustering established by 70% dDDH was found at 96.6% ANIb (MRI = 0.948). This result was not unexpected as inconsistencies within ANIb results have been previously shown [34]. In any case, both methods evidence incorrect and misleading species naming throughout the *Bradyrhizobium* genus. Among these, the most notorious are *B. japonicum* strains is5, in8p8, USDA 4, 22, USDA 124 and Is-1 and *B. elkanii* strains CCBAU 05737, USDA 94, USDA 3254, USDA 3259, WSM2783 and WSM1741. These strains do not belong to the species they have been assigned to, as dDDH and ANIb values are clearly below the species threshold compared with the type strains of these species (*B. japonicum* USDA 6^T^ and *B. elkanii* USDA 76^T^, Supplementary Data S1). *B. japonicum* strains is5, in8p8, 22 and Is-1 were assigned to the species level based on 16S rRNA [58]. In the case of *B. elkanii* strains USDA 94, USDA 3254 and USDA 3259 and *B. japonicum* USDA 4 and USDA 124, it has been previously suggested that they do not belong to these species based on MLSA results [59]. However, phylogenomics and specifically GBDP and ANI-based analysis provide a more accurate *in silico* species delimitation that can replace the conventional DNA-DNA hybridization “gold standard” [30,32].

### 3.2. Lateral and Subpolar Flagellar Systems in the Bradyrhizobium Genus

The genetic organization of the genes encoding each flagellar system of *B. diazoefficiens* USDA 110^T^ has been previously elucidated [10,19,21,22]. While genes of the subpolar flagellar system are distributed in at least four major clusters and several orphan genes, those of the lateral flagellar system are contiguous in the genome of *B. diazoefficiens* USDA 110^T^. Although this strain is not the only *Bradyrhizobium* species in which these two flagellar systems have been reported [22,25], to our knowledge, no extensive analysis in this genus regarding the distribution of flagellar systems has been done to date.

Phylogenomic GBDP-based analysis of 128 *Bradyrhizobium* genomes and the search for both flagellar systems have revealed that while all the genomes harbor the genes for the subpolar flagellar system, only 70 genomes present both flagellar systems (Figure 1, Supplementary Table S2). These genomes are phylogenetically linked, being all included within the *B. japonicum* PG (PG1). On the other hand, strains from PGs 2 to 13 only harbor the subpolar flagellar system. Interestingly, five genomes within the *B. japonicum* phylogroup (*B. japonicum* strains USDA 135 and 22, *B. diazoefficiens* NK6 and unclassified isolates Gha and Ghvi) do not contain genes for the lateral flagellum (Figure 1). These results, and the linkage of all the genes in a genetic cluster, might suggest that the lateral flagellar system could have been acquired by horizontal gene transfer (HGT), although certain genomes might have lost it afterwards. However, no evidence of HGT was found in the genomic sequences, such as signatures of direct repeats or GC% changes, perhaps because these traces can be diluted over time. Furthermore, *B. diazoefficiens* USDA 110^T^ is known to contain multiple genomic islands [60]. Nonetheless, none of the genes of the lateral or subpolar flagella are found within these previously identified genetic islands.

**Figure 2:**
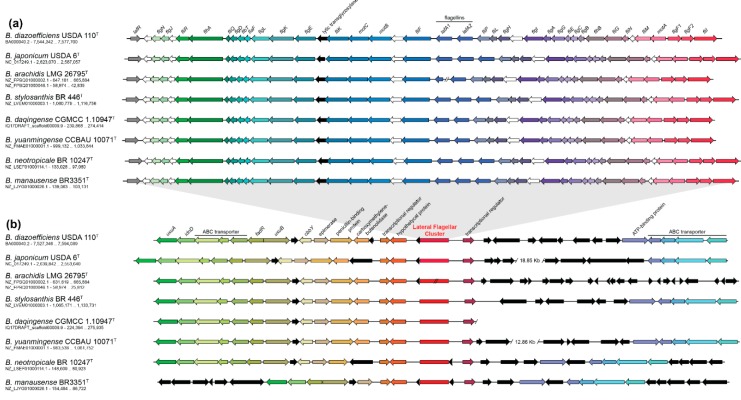
Synteny of the lateral flagellar system and genetic context among type strains of the *B. japonicum* PG. (**a**) Synteny of the lateral flagellar system showing high conservation and flagellins duplication events. White arrows indicate hypothetical proteins. (**b**) Synteny of the genetic context surrounding the lateral flagellar system (red square, not to scale). The upstream region synteny of the lateral flagellum is maintained across type strains while downstream region has suffered extensive genomic reorganizations. Black arrows represent hypothetical proteins and/or coding sequences with no homologous sequences in the genetic context among the genomes represented. NCBI accs. no. and coordinates of the regions shown are specified under the species name.

Phylogenetic MSLA of 35 concatenated CDSs of the lateral flagellar system in *Bradyrhizobium* genomes shows high agreement with the GBDP phylogenomic tree (Supplementary Figure S2). Furthermore, syntenic organization of the lateral flagellar genes in a genetic cluster is conserved throughout sequenced type-strains of the *B. japonicum* PG (*B. diazoefficiens* USDA 110^T^, *B. japonicum* USDA 6^T^, *B. arachidis* LMG 26795^T^, *B. stylosanthis* BR 446^T^, *B. daqigense* CGMCC 1.10947^T^, *B. yuanmingense* CCBAU 10071^T^, *B. neotropicale* BR 10247^T^ and *B. manausense* BR3351^T^), although duplication events in genes encoding lateral flagellins (*lafA*) are observed (Figure 2a). A variable number of flagellin genes has been previously observed across different *Bradyrhizobium* species and even outside this genus [19,21,61,62]. This level of conservation is also maintained within all genomes from the *B. japonicum* PG (Supplementary Figure S3). However, although there is synteny in the genomic region upstream, and the lateral flagellar cluster is consistent among all the type-strain genomes (Figure 2b), the downstream genomic region has undergone extensive rearrangements as only synteny of a transcriptional regulator and an ABC transporter cluster is found. In some genomes (*B. japonicum* USDA 6^T^ and *B. yuanmingense* CCBAU 10071^T^), this cluster is found several kilobases apart from the end of the lateral flagellar cluster (Figure 2b). In the case of the draft genomes *B. arachidis* LMG 26795^T^ and *B. daqigense* CGMCC 1.10947^T^, this cluster could not be found due to high genome fragmentation. Thus, the high degree of synteny maintenance within the lateral flagellar system and the upstream region, together with the lack of this flagellum in early-diverging groups, might suggest a unique HGT event as the origin of the lateral flagellar system in *Bradyrhizobium*, followed by a strictly vertical evolution. However, further analyses are required in order to test support the lateral flagellum HGT acquisition hypothesis.

Regarding the subpolar flagellar system, the available data show a high dispersion of genes through the chromosome in all the *Bradyrhizobium* genomes analyzed, which prevents the syntenic elucidation of the subpolar flagellum as a whole. In any case, the arrangement of genes within some clusters allows the distinction between each different flagellum.

### 3.3. Lateral Flagellar System Outside Bradyrhizobium

**Figure 3:**
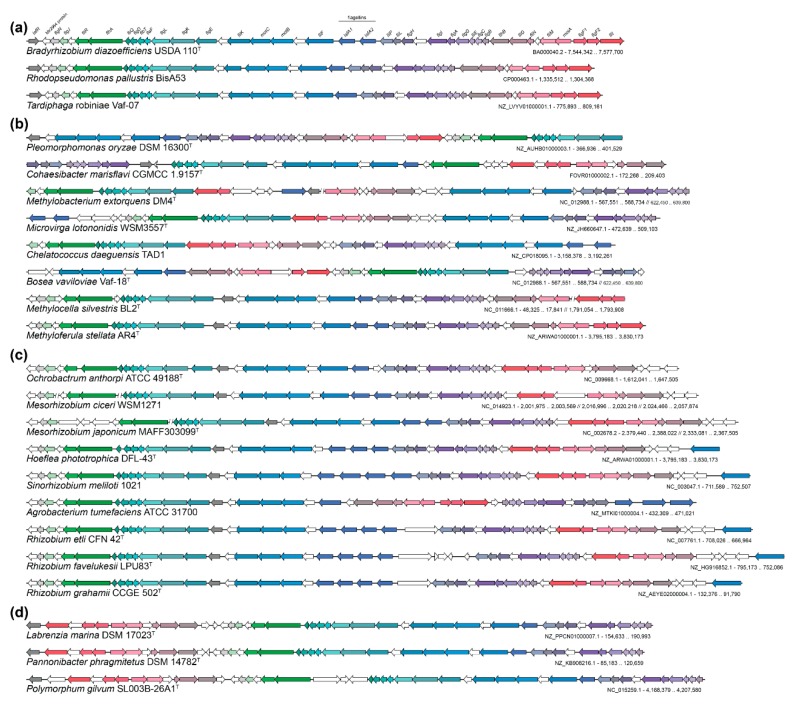
Synteny of the lateral flagellar system across representative *Rhizobiales* genomes and closest relatives outside *Rhizobiales*. (**a**) Synteny of the lateral flagellar systems of *Bradyrhizobium*, *Rhodopseudomonas* and *Tardiphaga* type strain species genomes. (**b**) Synteny of the lateral flagellar systems of diverging *Rhizobiales* genomes belonging to *Pleomorphomonas, Cohaesibacter, Methylobacterium, Microvirga*, *Chelatococcus*, *Bosea*, *Methylocella* and *Methyloferula* species. (**c**) Synteny of lateral flagellar systems of genomes belonging to *Ochrobactrum*, *Mesorhizobium*, *Hoeflea*, *Sinorhizobium*, *Agrobacterium* and *Rhizobium* species. (**d**) Synteny of the lateral flagellar systems of closest relative genomes outside *Rhizobiales* belonging to *Labrenzia*, *Pannonibacter* and *Polymorphum* species of the *Rhodobacteraceae* family. White arrows indicate hypothetical or unknown proteins or proteins with no involvement in flagellar biosynthesis or regulation. Species name, genome NCBI accs. no. and coordinates of the regions shown are specified under the syntenic representation of each genome.

In order to find the closest lateral flagellar systems outside *Bradyrhizobium*, 31 protein sequences of the lateral flagellum were used as queries in blastp against the whole non-redundant (nr) NCBI protein database. The results show the highest sequence homology with *Rhodopseudomonas palustris* BisA53 and *Tardiphaga* sp. OK245 (Supplementary Table S3), genera belonging to *Rhizobiales* order of *alpha-proteobacteria*. The lateral flagellar system of *R. palustris* BisA53 has been previously reported to be closely related to that of *B. diazoefficiens* USDA 110^T^ [22]. Interestingly, among 14 *Rhodopseudomonas* sequenced genomes, only two genomes (*R. palustris* strains BisA53 and BisB18) contain both, the lateral and subpolar flagellar systems, while the remaining genomes (*R. palustris* strains HaA2, JSC-3b, CGA009, BAL398, BisB5, TIE-1, DX-1, 42OL and ELI 1980, *R. pseudopalustris* DSM 123^T^ and unclassified isolates AAP120 and B29) only harbor the subpolar flagellum. On the other hand, *Tardiphaga* is another genus from the *Rhizobiales* order with only three sequenced genomes and they all have both, the lateral and the subpolar flagellar systems. Furthermore, syntenic organization of the lateral flagellum between these two genera and *Bradyrhizobium* is highly maintained, although a flagellin duplication in *Bradyrhizobium* has occurred (Figure 3a), which suggests a common origin of the lateral flagella.

Although *Rhodopseudomonas* and *Tardiphaga* are the closest relatives to the lateral flagellar system of *B. diazoefficiens* USDA 110^T^, and species from both genera also possess the subpolar flagellum, the blast results (Supplementary Table S3) show that other genera from the *Rhizobiales* order of *alpha-proteobacteria* possess the lateral flagellar system.

### 3.4. Flagellar Systems Distribution in Rhizobiales

To further analyze the presence of the subpolar and lateral flagellar systems in the *Rhizobiales* order, we performed a search on sequenced type-strain or reference species genomes for all the genera comprised within the *Rhizobiales* order. According to the NCBI Taxonomy, the *Rhizobiales* order is composed of 13 families, containing 130 genera and 741 named species of which only 292 species from 67 genera have sequenced genomes (Table 1, Supplementary Data S3). From the total number of sequenced species, 62.3% (182) present the lateral flagellum, while 28.8% (84) harbor the subpolar flagellum and 18.5% (54) do not present any flagellar system. On the other hand, 9.6% (28) of species from nine genera possess both flagellar systems. Most of these genera also contain species with only the subpolar system (*Bosea*, *Bradyrhizobium*, *Rhodopseudomonas*, *Methylobacterium* and *Pleomorphomonas*). We also found genera in which, aside from species with both flagella, there are also species with only the lateral flagellum (*Microvirga*) and genera in which all the sequenced species harbor both flagellar systems (*Tardiphaga*, *Salinarimonas* and *Cohaesibacter*) (Table 1), showing an uneven flagellar distribution throughout the *Rhizobiales* order.

**Figure 4:**
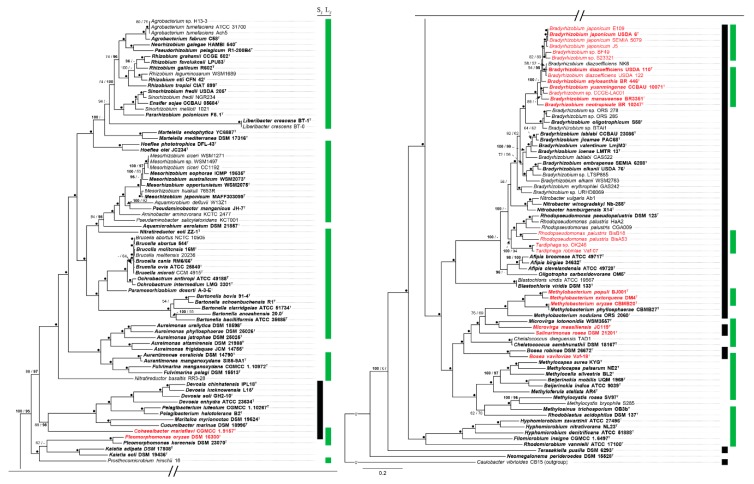
ML tree inferred from 104 concatenated single-copy amino acid sequences of 150 representative *Rhizobiales* genomes. *Caulobacter vibrioides* CB15 was used as outgroup. Bootstrap values under ML and MP tree searches are indicated above branches, left and right respectively. Dots indicate maximum support under all settings and values above 95% are shown in bold. Subpolar flagellum (S_F_, black), lateral flagellum (L_F_, green). Bold and ^T^ indicate type strain. Red typing indicates genomes with both flagellar systems. For additional information, see Supplementary Data S3.

The phylogenetic maximum-likelihood tree of 150 representative genomes belonging to 54 genera and 13 families of the *Rhizobiales* order, built with 104 amino acid sequences (Figure 4) is in agreement with previous accepted *Rhizobiales* order phylogenies [63,64]. Bootstrap support in both, the maximum likelihood (ML) and maximum parsimony (MP) tree searches, indicates a robust *Rhizobiales* phylogeny (Figure 4). Regarding the distribution of the flagellar systems across all examined species, we found that genera from the *Rhizobiaceae*, *Aurantimonadaceae*, *Phyllobacteraceae, Hyphomicrobiaceae, Beijerinckiaceae* and *Brucellaceae* families (i.e., *Agrobacterium*, *Rhizobium*, *Sinorhizobium*, *Mesorhizobium*, *Brucella*, *Ochrobactrum*, *Bartonella*, *Aureimonas* and *Aurantimonas*, among others) harbor only one flagellum that is homologous to the lateral flagellum of *B. diazoefficiens* USDA 110^T^ (Figure 4). On the other hand, species belonging to genera from the *Bradyrhizobiaceae* family (i.e., *Bradyrhizobium*, *Rhodopseudomonas*, *Tardiphaga*, *Nitrobacter*, *Afipia* and *Oligotropha*, among others) present flagella that are homologous to the subpolar flagellum of *B. diazoefficiens* USDA 110^T^. However, aside from the *B. japonicum* PG, the lateral flagellar system is also found in certain species of *Rhodopseudomonas* and in all *Tardiphaga* species (Figure 4). Interestingly, some genera phylogenetically close to *Bradyrhizobiaceae* family, such as *Beijerinckia*, *Methylocapsa*, *Methylocella*, *Methyloferula*, *Hyphomicrobium* and others, only possess the lateral flagellum. Also, dual flagellar systems are found in some of these genera, as is the case of *Methylobacterium*, *Microvirga*, *Salinarimonas* and *Bosea*, where certain species only harbor one of the flagellar systems (Figure 4).

Interestingly, a homologous subpolar flagellum Fla2 (according to conservation in some gene clusters, i.e., *fliQEflgCB*, *flgFGAH*, *flhBfliR*) is present in the related *alpha-proteobacteria* of the *Rhodobacteraceae* order *Rhodobacter sphaeroides* [9,63]. This finding does not agree with the type of flagellum that dominates in *Rhizobiales* (lateral flagellum). The fact that early-diverging lineages within this order only possess the lateral flagellum while some genera have retained only the subpolar flagellum, might suggests HGTs events, supposedly affecting the lateral flagellum, as the genetic organization of the subpolar flagellum scattered within the chromosome make its transference unlikely. Conversely, no genomic signatures or HGT evidence are found, and further analyses are required in order to either validate or refuse the HGT hypothesis of the lateral flagellum acquisition in *Rhizobiales*.

### 3.5. Flagellar Systems Phylogeny in Rhizobiales

**Figure 5:**
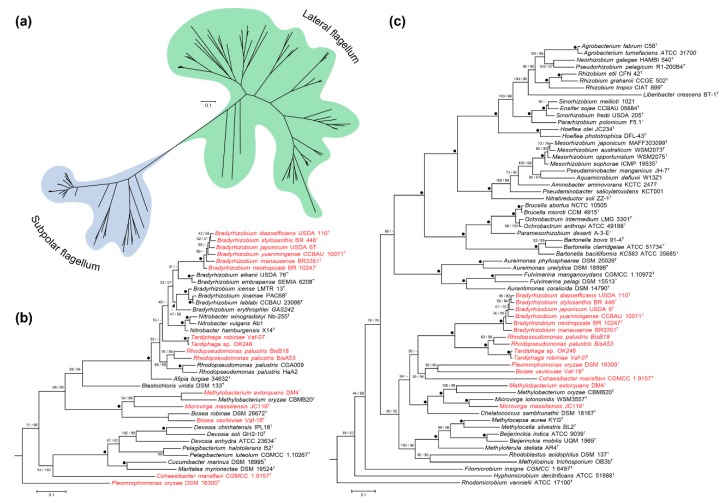
ML tree analysis of the subpolar and lateral flagellar systems across representative *Rhizobiales* genomes based on the concatenated sequences of 13 orthologous amino acid sequences, common to both flagella. Bootstrap values under ML and MP are indicated above branches. Dots indicate maximum support under all settings. (**a**) Subpolar and lateral flagellar clusters in unrooted tree. (**b**) Subpolar and (**c**) lateral flagellar system cluster trees were rooted using the other flagellar cluster as outgroup. Genomes harboring both flagellar systems are emphasized in red typing. Bold and ^T^ indicate type strain.

To address the relationship of both flagella across representative *Rhizobiales* genomes, a MLSA-based phylogeny was performed with 13 conserved and common amino acid sequences to both flagellar systems. As expected, the result shows a clear divergence of both flagella (Figure 5a). The subpolar flagellum branching pattern follows the same evolutive relationship than the observed in the phylogeny of *Rhizobiales* (Figure 5b, Figure 4), being divided into two major groups; one comprising *Bradyrhizobiaceae* family genomes and the related genera *Methylobacterium*, *Microvirga* and *Bosea*, and the other one comprising genera such as *Devosia*, *Pelagibacterium* and *Methylobacterium* among others. A similar branching pattern as observed in the *Rhizobiales* order phylogeny is also shown within the lateral flagellum (Figure 5c, Figure 4), being divided into two mayor groups; one comprising *Rhizobiaceae*, *Aurantimonadaceae* and *Phyllobacteraceae* families and the other one involving *Bradyrhizobiaceae* and related families. These subpolar and lateral flagellar system trees (Figure 5b,c) do not seem to support any HGT event because their speciation speed encompasses that of the phylogenetic *Rhizobiales* order tree.

Outside the *Rhizobiales* order, the closest lateral flagellum relatives are found in *Labrenzia, Pannonibacter* and *Polymorphum* species (Supplementary Table S4). They all belong to the *Rhodobacterales* order of *alpha-proteobacteria* and, based on previous phylogenetic analysis of the *Rhodobacteraceae* family, are very closely related [65]. Furthermore, syntenic organization of the lateral flagellum of these genera is strongly conserved and resembles the lateral flagellum of *Rhizobiales* order genomes (Figure 3d). Interestingly, *Labrenzia*, *Pannonibacter* and *Polymorphum* genomes also harbor a second flagellar gene set scattered through the chromosome, which is homologous to the subpolar flagellum of the *Rhizobiales* order. Additionally, this finding also shows that the lateral flagellar system is not only restricted to *Rhizobiales*, but it is also found in other *alpha-proteobacteria*. The prevalence of the lateral flagellum throughout different phylogenetically distant genera followed by subsequent loss events affecting certain species from genera such as *Bradyrhizobium*, might be attributed to the genetic structure of this flagellum in a single genetic cluster and the specific ecological niche these bacteria inhabit. However, the relevance of this flagellum as an adaptive trait should be also further analyzed, as in *Bradyrhizobium* no clear advantage has been uncovered to date.

## 4. Conclusions

Few studies have explored the distribution of flagellar systems in bacteria [1,9] until now. The present work makes a survey of the type and distribution of the flagellar systems within the *Rhizobiales* order. Analysis of the presence of the subpolar and lateral flagella in *Rhizobiales* sequenced species revealed an uneven flagellar distribution across this order. Dual flagellar systems are a rather uncommon phenomenon, affecting a small fraction of these species. The results presented here show that the lateral flagellar system is the most common flagellum within the order, while the subpolar flagellum is less abundant. The structure of the subpolar flagellum scattered throughout the chromosome in several clusters makes a transference event unlikely. Conversely, although genes of the lateral flagellum are clustered together, phylogenetic analysis of the flagellar systems did not show incongruencies compared to the species tree, which do not support horizontal transference but rather speciation. Nonetheless, there is a tendency for maintaining only one of the two flagella within the *Rhizobiales* order. The reasons underlying this observation might be related with flagellar incompatibilities or excessive energy consumption. If so, a bias towards flagellar loss is likely, although the consequences are different depending on the specific flagellum that is lost. From a functional point of view, the possible advantages for each flagellum would be dependent of the ecological benefits that might be derived from their presence. In the genus *Bradyrhizobium,* the tendency to maintain the lateral flagella in roughly half of the species probably suggests a still unknown function. In the case of *B. diazoefficiens* USDA 110^T^, studies indicate that its function could be related to swimming in viscous media and restraining cell adhesion on surfaces [22]. Furthermore, the lateral flagellum is the unique system present in the well-studied family of *Rhizobiaceae* and allows them to perform swimming and swarming motilities [66,67].

## Figures and Tables

**Table 1:** Distribution of the flagellar systems in 67 genera of the *Rhizobiales* order.

Family	Genus (Sequenced Species No.)	Subpolar Flagellum	Lateral Flagellum
*Aurantimonadaceae*	*Aurantimonas* (2)	-	+
	*Aureimonas* (5)	-	+
	*Fulvimarina* (2)	-	+
	*Martelella* (2)	n.m./n.d.	
*Bartonellaceae*	*Bartonella* (27)	-	+
*Beijerinckiaceae*	*Beijerinckia* (2)	-	+
	*Chelatococcus* (2)	-	+
	*Methylocapsa* (3)	-	+
	*Methylocella* (1)	-	+
	*Methyloferula* (1)	-	+
*Bradyrhizobiaceae*	*Afipia* (5)	+	-
	*Bosea* (4)	+	+/-
	*Bradyrhizobium* (25)	+	+/-
	*Nitrobacter* (3)	+	-
	*Oligotropha* (1)	+	-
	*Rhodoblastus* (1)	-	+
	*Rhodopseudomonas* (2)	+	+/-
	*Salinarimonas* (1)	+	+
	*Tardiphaga* (1)	+	+
*Brucellaceae*	*Brucella* (11)	-	+
	*Ochrobactrum* (9)	-	+
*Cohaesibacteraceae*	*Cohaesibacter* (1)	+	+
*Hyphomicrobiaceae*	*Blastochloris* (1)	+	-
	*Cucumibacter* (1)	+	-
	*Devosia* (11)	+	-
	*Filomicrobium* (1)	-	+
	*Hyphomicrobium* (5)	-	+
	*Maritalea* (1)	+	-
	*Pelagibacterium* (2)	+	-
	*Prosthecomicrobium* (1)	+	-
	*Rhodomicrobium* (2)	-	+
	*Meganema* (1)	n.m./n.d.	
*Methylobacteriaceae*	*Methylobacterium* (20)	+	+/-
	*Microvirga* (6)	+/-	+
	*Neomegalonema* (1)	n.d.	
*Methylocystaceae*	*Methylocystis* (3)	-	+
	*Methylosinus* (1)	-	+
	*Pleomorphomonas* (2)	+	+/-
	*Terasakiella* (1)	+	-
*Phyllobacteriaceae*	*Aminobacter* (1)	-	+
	*Aquamicrobium* (2)	-	+
	*Hoeflea* (2)	-	+
	*Mesorhizobium* (17)	-	+
	*Nitratireductor* (5)	-	+
	*Paramesorhizobium* (1)	-	+
	*Pseudaminobacter* (2)	-	+
*Rhizobiaceae*	*Kaistia* (3)	n.m./n.d.	
	*Agrobacterium* (9)	-	+
	*Neorhizobium* (1)	-	+
	*Pararhizobium* (2)	-	+
	*Pseudorhizobium* (1)	-	+
	*Rhizobium* (42)	-	+
	*Ensifer* (5)	-	+
	*Sinorhizobium* (6)	-	+
*Xanthobacteraceae*	*Ancylobacter* (1)	-	+
	*Azorhizobium* (2)	-	+
	*Pseudoxanthobacter* (1)	-	+
	*Starkeya* (1)	n.m./n.d.	
	*Xanthobacter* (1)	n.d.	
unclassified *Rhizobiales*	*Bauldia* (1)	n.d.	
	*Methylobrevis* (1)	+	-
	*Methyloceanibacter* (5)	n.d.	
	*Methyloligella* (1)	n.m./n.d.	
	*Pseudorhodoplanes* (1)	+	-

Taxonomy according to NCBI Taxonomy database. Last accessed in October 2017. For extended information, see Supplementary Data S3. n.d.: Distinctive genes of the flagellar systems as described in material and methods not detected. n.m.: Non-motile bacteria. For references, see Supplementary Data S3.

## Supplementary Materials

The following are available online at http://www.mdpi.com/2076-2607/7/2/50/s1, Figure S1: ANIb phylogenomic tree of 128 *Bradyrhizobium* genomes and clusters at phylogenetics groups and at species levels, Figure S2: Unrooted ML tree based on the concatenated sequences of 35 lateral flagellum CDSs of 66 *Bradyrhizobium* genomes, Figure S3: Syntenic organization of the lateral flagellar gene cluster among 66 *Bradyrhizobium* genomes, Table S1: General genomic features of genomes used in this study, Table S2: Subpolar and lateral flagellar systems distribution in sequenced *Bradyrhizobium* genomes used in this study, Table S3: First 10 closest relatives of 31 *Bradyrhizobium diazoefficiens* USDA 110^T^ protein sequences of the lateral flagellar system, Table S4: Blastx of 13 lateral flagellum genes of 10 representative *Rhizobiales* genomes against the nr NCBI protein database excluding *Rhizobiales* order genomes, Data S1: GBDP and ANIb comparisons of *Bradyrhizobium* genomes, Data S2: Clustering of *Bradyrhizobium* intergenomic distances for phylogenomic groups and species identification based on GBDP and ANIb, Data S3: Subpolar and lateral flagellar systems distribution in sequenced representative *Rhizobiales* order species.
