# Supplementary material for: Phylogenomic Analyses of Bradyrhizobium Reveal Uneven Distribution of the Lateral and Subpolar Flagellar Systems, Which Extends to Rhizobiales
Source: Microorganisms. 2019 Feb 13;7(2):50. doi: 10.3390/microorganisms7020050 (PMC6406911; doi:10.3390/microorganisms7020050)
Supplement: Supplementary file 1 [file Supplementary_Figure_S1.pdf › Supplementary_Figure_S1.pdf]

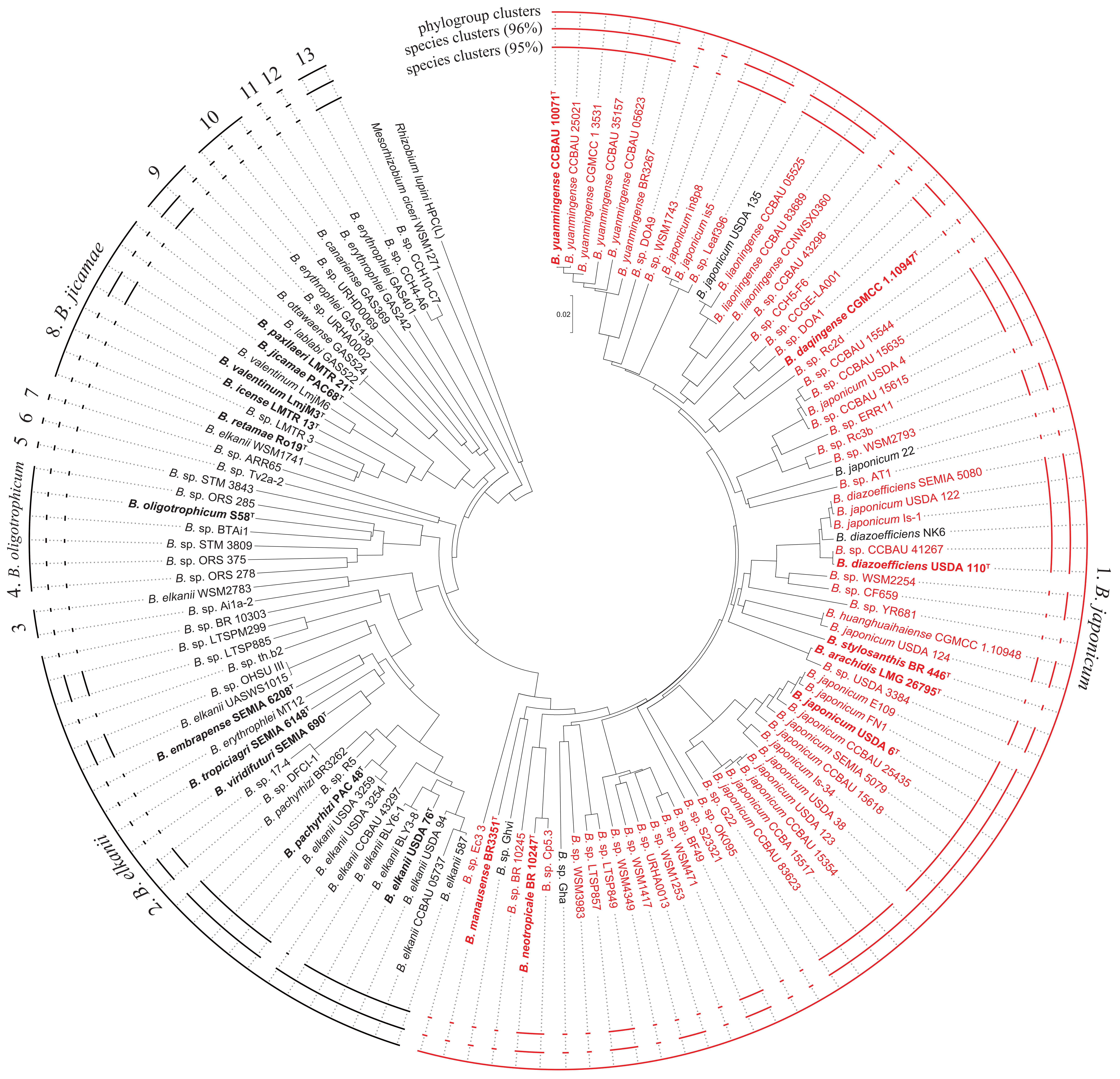

Supplementary Figure S1. Phylogenomic tree based on ANIb intergenomic distances of 128 *Bradyrhizobium* genomes and clusters at phylogenetic groups and at species levels (ANIb 95% and 96%). *Rhizobium lupini* HPC(L) and *Mesorhizobium ciceri* WSM1271 were used as outgroups. Only genomes clustered within the PG1 (*B. japonicum*) harbor both, the lateral and subpolar flagellar systems (red typing), while genomes of the remaining PGs (2 to 13) and some within the PG1 only harbor the subpolar flagellar system (black typing). Bold and ^{T} indicate type strain. Phylogroups and species clusters according to ANIb intergenomic distance-based clustering (see Supplementary Data S1).
