# Supplementary material for: Phylogenomic Analyses of Bradyrhizobium Reveal Uneven Distribution of the Lateral and Subpolar Flagellar Systems, Which Extends to Rhizobiales
Source: Microorganisms. 2019 Feb 13;7(2):50. doi: 10.3390/microorganisms7020050 (PMC6406911; doi:10.3390/microorganisms7020050)
Supplement: Supplementary file 1 [file Supplementary_Figure_S3.pdf › Supplementary_Figure_S3.pdf]

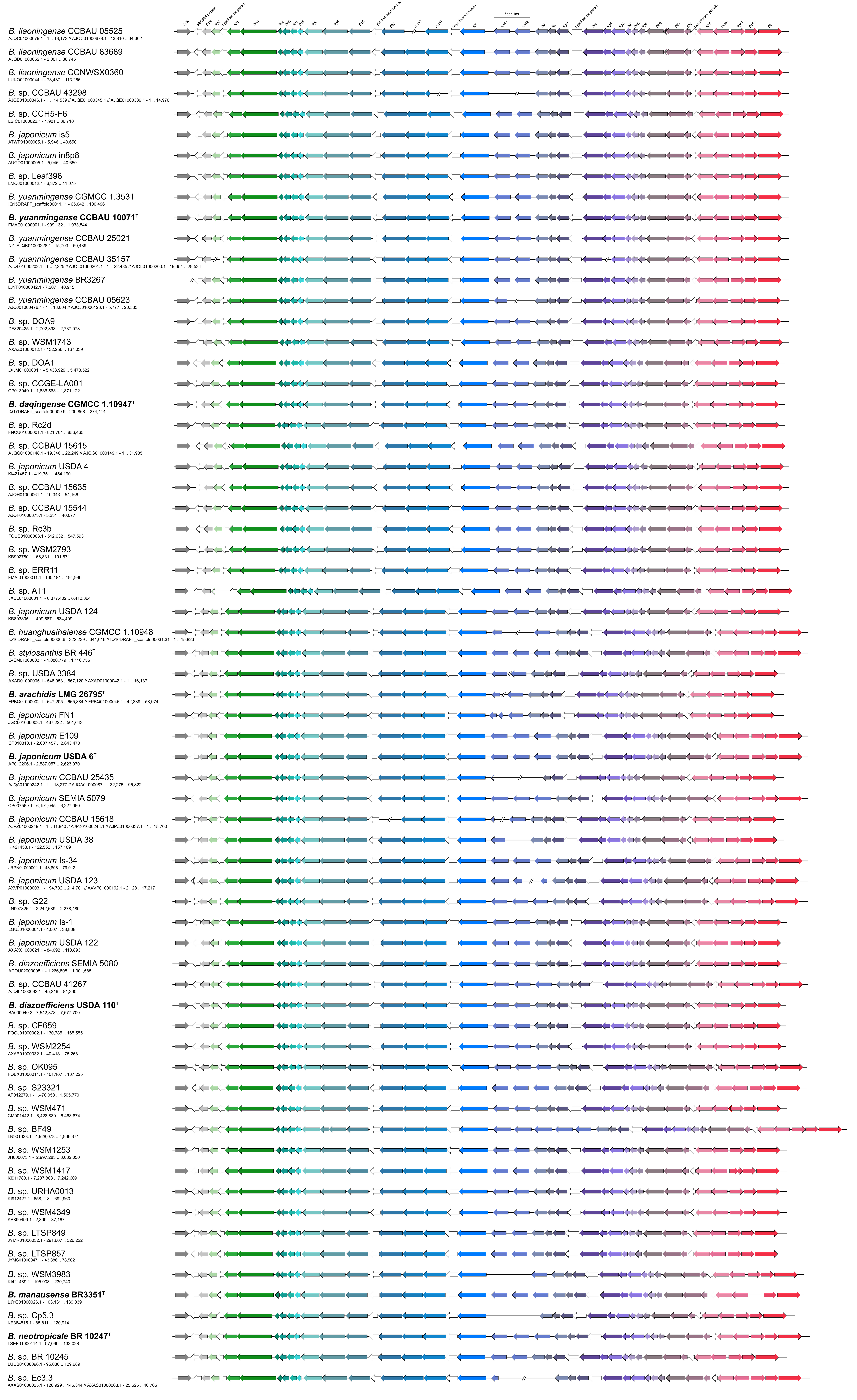

Supplementary Figure S3. Syntenic organization of the lateral flagellar gene cluster among 66 Bradyrhizobium genomes harboring both, the lateral and subpolar flagellar systems. High degree of conservation and flagellin duplications are observed. White arrows represent hypothetical or unknown proteins or coding sequences with no involvement in flagellar biosynthesis or regulation. Genome NCBI accs. no. and coordinates of the regions shown are specified under the strains name. Bold and T indicate type strain.
