# Supplementary material for: Phylogenomic Analyses of Bradyrhizobium Reveal Uneven Distribution of the Lateral and Subpolar Flagellar Systems, Which Extends to Rhizobiales
Source: Microorganisms. 2019 Feb 13;7(2):50. doi: 10.3390/microorganisms7020050 (PMC6406911; doi:10.3390/microorganisms7020050)
Supplement: Supplementary file 1 [file microorganisms-07-00050-s001.zip › Supplementary_Data_S3.pdf]

Supplementary Data S3. Subpolar and lateral flagellar systems distribution in sequenced representative *Rhizobiales* order species

Family	Genus	Species/Genome[a]	No. of sequenced genomes[b]	Subpolar flagellum	Lateral flagellum	Subpolar flagellum ascc. No	Lateral flagellum ascc. No.	Reference
Aurantimonadaceae	*Aurantimonas*	*A. coralicida* DSM 14790[T]	2	-	+		BBWN01000012.1	
		A. endophytica KCTC 52296[T]	0					
		A. litoralis KCTC 12094[T]	0					
		A. manganoxydans DSM 21871[T]	2	-	+		CH672387.1	
	Aureimonas	*A. altamirensis* DSM 21988[T]	3	n.m/n.d.				Jurado et al., 2006 (10.1099/ijs.0.64397-0)
		A. endophytica KCTC 52217[T]	0					
		A. ferruginea JCM 18444[T]	0					
		A. frigidaque JCM 14755[T]	1	n.d.				
		A. glaciei KCTC 52359[T]	0					
		A. glaciistagni JCM 30183[T]	0					
		A. rubiginis JCM 18445[T]	0					
		A. ureilytica DSM 18598[T]	4	-	+		ARQE01000005.1	
		A. jatrophae DSM 25025[T]	1	-	+		FNIT01000006.1	
		A. phyllosphaerae DSM 25026[T]	1	-	+		FOOA01000003.1	
	Fulvimarina	*F. manganoxydans* CGMCC 1.10972[T]	1	-	+		FWXR01000006.1	
		F. pelagi DSM 15513[T]	2	-	+		BBWO01000012.1	
	Jiella	*J. aquimaris* JCM 30119[T]	0					
	Martelella	*M. endophytica* YC6887[T]	1	n.m./n.d.				Bibi et al., 2013 (10.1099/ijs.0.048785-0)
		M. mangrovi DSM 28102[T]	0					
		M. mediterranea DSM 17316[T]	2	n.m/n.d.				Rivas et al., 2005 (10.1099/ijs.0.63438-0)
		M. radicis DSM 28101[T]	0					
Bartonellaceae	*Bartonella*	*B. acomydis* KCTC 23907[T]	0					
		B. alsatica IBS 382[T]	1	n.d.				
		B. ancashensis 20.00[T]	1	-	+		CP010401.1	
		B. apis PEB0122[T]	6	-	+		LXYU01000002.1	
		B. australis Aust/NH1[T]	1	n.d.				
		B. bacilliformis ATCC 35685[T]	16	-	+		CP000524.1	
		B. birtlesii IBS 325[T]	6	n.d.				
		B. bovis 91-4[T]	2	-	+		CM001844.1	
		B. callosciuri KCTC 23909[T]	0					
		B. capreoli DSM 21569[T]	0					
		B. chomelii DSM 21431[T]	0					
		B. clarridgeiae ATCC 51734[T]	2	-	+		KI912398.1	
		B. coopersplainsensis DSM 23499[T]	0					
		B. doshiae ATCC 700133[T]	3	n.d.				
		B. durdenii	0					
		B. elizabethae ATCC 49927[T]	4	n.d.				
		B. florencae R4[T]	1	n.d.				
		B. fuyuanensis DSM 100694[T]	0					
		B. grahamii ATCC 700132[T]	2	n.d.				
		B. heixiaziensis DSM 100695[T]	0					
		B. henselae Houston-1[T]	23	n.d.				
		B. jaculi KCTC 23655[T]	0					
		B. japonica DSM 23650[T]	0					
		B. koehlerae C-29[T]	1	n.d.				
		B. massiliensis	0					
		B. melophagi K-2C	1	-	+		JH725084.1	
		B. pachyuromydis	0					

		B. peromysci KCTC 23657^{T}	0					
		B. phoceensis CIP 107707^{T}	0					
		B. queenslandensis Aust/NH15	1	n.m./n.d.				Gundi et al., 2009 (10.1099/ijs.0.002865-0)
		B. quintana RM-11	14	n.d.				
		B. rattaustraliani Aust/NH4^{T}	1	n.m./n.d.				Gundi et al., 2009 (10.1099/ijs.0.002865-0)
		B. rattimassiliensis 15908	2	n.m./n.d.				Gundi et al., 2004 (10.1128/JCM.42.8.3816-3818.2004)
		B. rochalimae ATCC BAA-1498^{T}	1	-	+		FN645457.1	
		B. schoenbuchensis R1^{T}	*2*	-	+		CP019789.1	
		B. senegalensis OS02^{T}	1	n.m./n.d.				Mediannikov et al., 2013 (10.4056/sigs.3807472)
		B. silvatica DSM 23649^{T}	0					
		B. silvicola	0					
		B. talpae	0					
		B. tamiae Th307	2	n.d.				
		B. taylorii 8TBB	2	n.d.				
		B. tribocorum CIP 105476^{T}	2	n.d.				
		B. vinsonii Winnie	5	n.d.				
		B. volans	0					
		B. washoensis Sb944nv	2	n.d.				
		B. weisii	0					
Beijerinckiaceae	*Beijerinckia*	*B. derxii* DSM 2328^{T}	0					
		B. doebereinerae LMG 2819^{T}	0					
		B. fluminensis CIP 106281^{T}	0					
		B. indica ATCC 9039^{T}	1	-	+		CP001016.1	
		B. mobilis UQM 1969^{T}	1	-	+		JQJH01000002.1	
	Camelimonas	*C. abortus* DSM 24741^{T}	0					
		C. fluminis KCTC 42282^{T}	0					
		C. lactis CCUG 58638^{T}	0					
	Chelatococcus	*C. csaccharovorans* DSM 6462^{T}	0					
		C. caeni KCTC 32487^{T}	0					
		C. daeguensis TAD1	2	-	+		CP018095.1	
		C. reniformis JCM 30308^{T}	0					
		C. sambhunathii DSM 18167^{T}	2	-	+		LIOL01000001.1	
	Methylocapsa	*M. acidipphila* B2^{T}	1	n.m./n.d.				Dedysh et al., 2002 (10.1099/00207713-52-1-251)
		M. aurea KYG^{T}	1	-	+		JQKO01000014.1	
		M. palsarum NE2^{T}	1	-	+		FOSN01000001.1	
	Methylocella	*M. palustris* ATCC 700799^{T}	0					
		M. silvestris BL2^{T}	1	-	+		NC_011666.1	
		M. tundrae DSM 15673^{T}	0					
	Methyloferula	*M. stellata* AR4^{T}	1	-	+		ARWA01000001.1	
	Methylorosula	*M. polaris* DSM 22001^{T}	0					
	Methylovirgula	*M. ligni* DSM 19998^{T}	0					
	Pseudochelatococcus	*P. contaminans* LMG 28285^{T}	0					
		P. lubricantis LMG 28286^{T}	0					
Bradyrhizobiaceae	*Afipia*	*A. birgiae* 34632^{T}	1	+	-	HG326654.1		
		A. broomeae ATCC 49717^{T}	1	+	-	AGWX01000004.1		
		A. clevelandensis ATCC 49720^{T}	1	+	-	AGWY01000007.1		
		A. felis ATCC 53690^{T}	2	+	-	AGWZ01000001.1		
		A. lausannensis	0					
		A. massiliensis LC387^{T}	1	+	-	LBIA01000001.1		
	Blastobacter	*B. henricii*	0					
	Bossea	*B. eneae* CIP 106338^{T}	0					
		B. lathyri LMG 26379^{T}	0					
		B. lupini LMG 26383^{T}	1	+	-	FOAN01000010.1		
		B. massilensis CIP 106336^{T}	0					

	B. manatitlanensis ATCC 700918^{T}	0				
	B. robinae DSM 26672^{T}	1	+	-	FNBZ01000002.1	
	B. thiooxidans DSM 9653^{T}	2	+	-	FUYX01000001.1	
	B. vaviloviae **Vaf-18^{T}**	**2**	**+**	**+**	**CP017147.1**	**CP017147.1**
	B. vestrisii CIP 106340^{T}	0				
Bradyrhizobium	*B. americanum* LMG 29514^{T}	0				
	B. arachidis **LMG 26795^{T}**	**1**	**+**	**+**	**FPBQ01000003.1**	**FPBQ01000002.1**
	B. betae IMG 21987^{T}	0				
	B. canariense GAS369	15	+	-	LT629750.1	
	B. centrosemantis LMG 29515^{T}	0				
	B. cytisi LMG 25866^{T}	0				
	B. daqingense **CGMCC 1.10947^{Tc}**	**1**	**+**	**+**		
	B. denitrificans ATCC 43295^{T}	0				
	B. diazoefficiens **USDA 110^{T}**	**5**	**+**	**+**	**BA000040.2**	**BA000040.2**
	B. elkanii USDA 76^{T}	13	+	-	KB900701.1	
	B. embrapense SEMIA 6208^{T}	1	+	-	LFIP02000003.1	
	B. erythrophlei MT12	5	+	-	FNTH01000001.1	
	B. ferriligni CCBAU 51502^{T}	0				
	B. ganzhouense CCAU 101088^{T}	0				
	B. guangdongense LMG 28620^{T}	0				
	B. guangxiense LMG 28621^{T}	0				
	B. icense LMTR 13^{T}	1	+	-	CP016428	
	B. ingae HAMBI 3600^{T}	0				
	B. iriomotense LMG 24129^{T}	0				
	B. japonicum **USDA 6^{T}**	**23**	**+**	**+**	**AP012206.1**	**AP012206.1**
	B. jicamae PAC68^{T}	1	+	-	LLXZ01000016.1	
	B. kavangense DSM 100299^{T}	0				
	B. lablabi CCBAU 23086^{T}	4	+	-	LLYB01000082.1	
	B. liaonningense **CCNWSX0360**	**3**	**+**	**+**	**LUKO01000219.1**	**LUKO01000044.1**
	B. manausense **BR3351^{T}**	**1**	**+**	**+**	**LJYG01000054.1**	**LJYG01000026.1**
	B. mercantei SEMIA 6399^{T}	1	+	-	MKFI01000001.1	
	B. neotropicale **BR 10247^{T}**	**1**	**+**	**+**	**LSEF01000057.1**	**LSEF01000114.1**
	B. oligotrophicum S58^{T}	1	+	-	NC_020453.1	
	B. ottawaense GAS524	2	+	-	LT629693.1	
	B. pachyrhizi PAC48^{T}	2	+	-	LFIQ01000120.1	
	B. paxllaeri LMTR 21^{T}	1	+	-	MAXB01000079.1	
	B. retamae Ro19^{T}	1	+	-	LLYA01000191.1	
	B. rifense LMG 26781^{T}	0				
	B. stylosanthis BR 446^{T}	1	+	-	LVEM01000001.1	
	B. subterraneum DSM 100298^{T}	0				
	B. tropiciagri SEMIA 6148^{T}	1	+	-	LFIP02000003.1	
	B. valentinum LmjM3^{T}	2	+	-	LLXX01000235.1	
	B. vignae DSM 100297^{T}	0				
	B. viridifuturi SEMIA 690^{T}	1	+	-	LGTB01000070.1	
	B. yuanmingense **CCBAU 10071^{T}**	**5**	**+**	**+**	**FMAE01000011.1**	**FMAE01000001.1**
Nitrobacter	*N. alkalicus* LMD 97.163^{T}	0				
	N. hamburgensis X14^{T}	1	+	-	NC_007964	
	N. vulgaris Ab1	1	+	-	MWPQ01000026.1	
	N. winogradskyi Nb-255^{T}	1	+	-	NC_007406	
Oligotropha	*O. carboxidovorans* ATCC 49405^{T}	3	+	-	CP001196.1	
Photorhizobium	*P. thompsonianum*	0				
Rhodoblastus	*R. acidophilus* DSM 137^{T}	1	-	+		NZ_FYDG01000006.1
	R. sphagnicola DSM 16996^{T}	0				
Rhodopseudomonas	*R. boonkerdii* NBRC 106595^{T}	0				

		R. faecalis JCM 11668^{T}	0					
		R. harwoodiae NBRC 107575^{T}	0					
		R. julia DSM 11549^{T}	0					
		R. lichen	0					
		R. oryzae	0					
		R. palustris **BisA53**	**11**	**+**	**+**	**CP000463.1**	**CP000463.1**	
		R. parapalustris NBRC 106083^{T}	0					
		R. pentothenatexigens NBRC 108862^{T}	0					
		R. pseudopalustris DSM 123^{T}	1	+	-	FODT01000007.1		
		R. rhenobacensis DSM 12706^{T}	0					
		R. telluris NBRC 107609^{T}	0					
		R. thermotolerans NBRC 108863^{T}	0					
	Salinarimonas	*S. ramus* DSM 22962^{T}	0					
		S. rosea **DSM 21201^{T}**	**1**	**+**	**+**	**AUBC01000001.1**	**AUBC01000006.1**	
	Tardiphaga	***T. robiniae*** **Vaf-07**	**1**	**+**	**+**	**LVYV01000034.1**	**LVYV01000001.1**	
	Variibacter	*V. gotjawalensis* LMG 28093^{T}	0					
Brucellaceae	*Brucella*	*B. abortus* 544^{T}	197	-	+		AQIS01000009.1	
		B. canis RM6/66^{T}	20	-	+		CP007759.1	
		B. ceti TE28753-12	9	-	+		CP006899.1	
		B. inopinata BO1^{T}	2	-	+		ADEZ01000028.1	
		B. melitensis 16M^{T}	155	-	+		AE008918.1	
		B. microti CCM 4915^{T}	1	-	+		CP001579.1	
		B. neotomae 5K33^{T}	3	-	+		JMSC01000004.1	
		B. ovis ATCC 25840^{T}	16	-	+		CP000709.1	
		B. papionis NCTC 13660^{T}	0					
		B. pinnipedialis B2/94	5	-	+		CP002079.1	
		B. suis 1330^{T}	58	-	+		AE014292.2	
		B. vulpis F60^{T}	2	-	+		LN997864.1	
	Daeguia	*D. caeni* KCTC 12981^{T}	0					
	Falsochrobactrum	*F. ovis* LMG 27356^{T}	0					
	Mycoplana	*M. dimorpha* ATCC 4279^{T}	0					
		M. peli	0					
		M. ramosa ATCC 49678^{T}	0					
	Ochrobactrum	*O. anthropi* ATCC 49188^{T}	15	-	+		CP000759.1	
		O. ciceri DSM 22292^{T}	0					
		O. cytisi IPA7.2	1	-	+		MOEC01000003.1	
		O. daejeonense KCTC 22458^{T}	0					
		O. endophyticum DSM 29930^{T}	0					
		O. gallinifaecis DSM 15295^{T}	0					
		O. grignonense OgA9aT	1	-	+		NNRL01000162.1	
		O. guangzhouense	0					
		O. haematophilum CIP 109452^{T}	0					
		O. intermedium LMG 3301^{T}	8	-	+		ACQA01000002.1	
		O. lupini LUP21^{T}	1	-	+		NNRN01000065.1	
		O. oryzae DSM 17471^{T}	0					
		O. pecoris DSM 23868^{T}	0					
		O. pituitosum AA22	1	-	+		CP018782.1	
		O. pseudointermedium DSM 17490^{T}	0					
		O. pseudogrignonense CCUG 30717^{T}	2	-	+		NNRM01000020.1	
		O. rhizosphaerae PR17^{T}	2	-	+		NNRK01000035.1	
		O. shirianus	0					
		O. thiophenivorans DSM 7216^{T}	1	n.m./n.d.				Kämpfer et al., 2008 (10.1099/ijs.0.65407-0)
		O. tritici DSM 13340^{T}	0					
	Paenochrobactrum	*P. gallinarii* CCUG 57736^{T}	0					

		P. glaciei JCM 15115^T	0				
		P. pullorum LMG 28095^T	0				
	Pseudochrobactrum	*P. asaccharolytium* CIP 108977^T	0				
		P. kiredjianiae CIP 109227^T	0				
		P. lubricantis CCUG 56963^T	0				
		P. saccharolyticum CIP 108976^T	0				
Cohaesibacteraceae	*Breoghania*	*B. corrubedonensis* DSM 23382^T	0				
	Cohaesibacter	*C. gelatinilyticus* DSM 18289^T	0				
		C. halooalkalitolerans NBRC 109022^T	0				
		***C. marisflavi* CGMCC 1.9157^T**	**1**	**+**	**+**	**FOVR01000010.1**	**FOVR01000002.1**
Hyphomicrobiaceae	*Ancalomicrobium*	*A. adetum* DSM 4722^T	0				
	Angulomicrobium	*A. amanitiforme* DSM 15561^T	0				
		A. tetraedrale DSM 5895^T	0				
	Aquabacter	*A. spiritensis* DSM 9035^T	0				
	Arsenicitalea	*A. aurantica* KCTC 42835^T	0				
	Blastochloris	*B. gulmargensis* DSM 19786^T	0				
		B. sulfoviridis DSM 729^T	0				
		B. viridis DSM 133^T	3	+	-	AP014854.2	
	Cucumibacter	*C. marinus* DSM 18995^T	1	+	-	AUCR01000009.1	
	Devosia	*D. albogilva* MTCC 8594^T	0				
		D. chinhatensis IPL18^T	1	+	-	JZEY01000054.1	
		D. confluentis NBRC 112271^T	0				
		D. crocina IPL20^T	1	+	-	FPCK01000001.1	
		D. enhydra ATCC 23634^T	1	+	-	FPKU01000001.1	
		D. epidermidihirudinis E84^T	1	+	-	LANJ01000045.1	
		D. geojensis BD-c194^T	1	+	-	JZEX01000147.1	
		D. ginsengisoli	0				
		D. glacialis LMG 26051^T	0				
		D. honganensis ATCC 19737^T	0				
		D. humi KACC 18281^T	0				
		D. hwasunensis	0				
		D. insulae DS-56^T	1	+	-	LAJE02000291.1	
		D. limi DSM 17137^T	2	+	-	FQVC01000004.1	
		D. lucknowensis L15^T	1	+	-	FXWK01000001.1	
		D. mishustinii CCM 7569^T	0				
		D. nanyangense	0				
		D. neptuniae LMG 21357^T	0				
		D. pacifica KCTC 32437^T	0				
		D. psychrophila Cr7-05^T	2	+	-	LAPV01000086.1	
		D. riboflavina IFO13584^T	1	+	-	JQGC01000035.1	
		D. soli GH2-10^T	1	+	-	LAJG01000014.1	
		D. subaequois KCTC 12772^T	0				
		D. submarina NRIC 0884^T	0				
		D. terrae	0				
		D. yakushimensis LMG 24299^T	0				
	Dichotomicrobium	*D. thermohalophilum* DSM 5002^T	0				
	Filomicrobium	*F. fusiforme* DSM 5304^T	0				
		F. insigne CGMCC 1.6497^T	1	-	+		FNJC01000002.1
	Hyphomicrobium	*H. aestuarii* ATCC 27483^T	0				
		H. chloromethanicum NCIMB 13687^T	0				
		H. denitrificans ATCC 51888^T	2	-	+		CP002083.1
		H. facile DSM 1565^T	1	-	+		FPCH01000003.1
		H. hollandicum ATCC 27498^T	0				
		H. methylovorum DSM 5458^T	0				

		H. nitrativorans NL23^T	1	-	+		NC_022997.1
		H. sulfonivorans WDL6	1	-	+		LMTR01000065.1
		H. vulgare ATCC 33404^T	0				
		H. zavarzinii ATCC 27496^T	1	-	+		KB911255.1
	Maritalea	*M. mobilis* JCM 15144^T	0				
		M. myrionectae DSM 19524^T	1	+	-	AUHV01000003.1	
		M. porphyrae LMG 25872^T	0				
	Methylorhabdus	*M. multivorans* ATCC 51890^T	0				
	Methyloterrigena	*M. soli* JCM 30821^T	0				
	Paradevosia	*P. shaoguanensis* LMG 27409^T	0				
	Pedomicrobium	*P. americanum* ATCC 43612^T	0				
		P. australicum ATCC 43611^T	0				
		P. ferrugineum ATCC 33119^T	0				
		P. manganicum ATCC 33121^T	0				
	Pelagibacterium	*P. halotolerans* B2^T	1	+	-	NC_016078.1	
		P. lentulum KCTC 52551^T	0				
		P. luteolum CGMCC 1.10267^T	1	+	-	FNCS01000013.1	
		P. nitratireducens JCM 17767^T	0				
		P. rhizosphaerae KACC 18791^T	0				
	Polyprosthecobacterium	*P. yinchuanense*	0				
	Prosthecomicrobium	*P. hirschii* 16	1	+	-	LJYW01000001.1	
		P. pneumaticum DSM 8972^T	0				
	Rhodomicrobium	*R. udaipurense* JA643^T	1	-	+		JFZJ01000032.1
		R. vannielii ATCC 17100^T	2	-	+		CP002292.1
	Rhodoplanes	*R. elegans* DSM 11907^T	0				
		R. oryzae NBRC 109406^T	0				
		R. piscinae KCTC 5627^T	0				
		R. pokkaliisoli NBRC 104972^T	0				
		R. roseus DSM 5909^T	0				
		R. serenus DSM 18633^T	0				
		R. tepidamans DSM 9987^T	0				
	Robiginosimarina	*R. monoshimaensis*	0				
	Seliberia	*S. tellata* JCM 21594^T	0				
	Youhaiella	*Y. tibetensis* JCM 19854^T	0				
	Meganema	*M. perideroedes* DSM 15528^T	1	n.m./n.d.			McIlroy et al., 2015 (10.1186/s40793-015-0013-1)
Methylobacteriaceae	*Enterovirga*	*E. rhinocerotis* DSM 25903^T	0				
	Methylobacterium	*M. adhaesivum* CECT 7069^T	0				
		M. aerolatum DSM 19013^T	0				
		M. aminovorans ATCC 51358^T	0				
		***M. aquaticum* DSM 16371^T**	**5**	**+**	**+**	**LABX01000173.1**	**LABX01000132.1**
		***M. branchiatum* 111MFTsu3.1M4**	**1**	**+**	**+**	**FOQW01000013.1**	**FOQW01000020.1**
		M. brachythecii DSM 24105^T	0				
		M. bullatum DSM 21893^T	0				
		M. cerastii DSM 23679^T	0				
		M. dankookense DSM 22415^T	0				
		***M. extorquens* DM4^T**	**7**	**+**	**+**	**NC_012988.1**	**NC_012988.1**
		M. fujisawaense DSM 5686^T	0				
		M. gnaphalii DSM 24027^T	0				
		M. goesingense DSM 21331^T	0				
		M. gossipiicola Gh-105^T	1	+	-	FOPM01000007.1	
		M. gregans DSM 19564^T	0				
		M. haplocladii DSM 24195^T	0				
		M. hispanicum DSM 16372^T	0				
		***M. indicum* SE2.11^T**	**2**	**+**	**+**	**JTHF01000313.1**	**JTHF01000098.1**

		M. iners DSM 19015^{T}	0					
		M. isbiliense CECT 7068^{T}	0					
		M. jeotgali LMG 23639^{T}	0					
		M. komagatae DSM 19563^{T}	0					
		M. longum DSM 23933^{T}	0					
		M. marchantiae DSM 21328^{T}	0					
		M. mesophilicum SR1.6/6	1	+	-	ANPA01000018.1		
		M. nodulans ORS 2060^{T}	1	+	-	NC_011894.1		
		M. organophilum DSM 760^{T}	0					
		***M. oryzae* CBMB20^{T}**	**1**	**+**	**+**	**CP003811.1**	**CP003811.1**	
		M. oxalidis DSM 24028^{T}	0					
		M. persicinum DSM 19562^{T}	0					
		M. phyllosphaerae CBMB27^{T}	3	+	-	CP015367.1		
		***M. phyllostachyos* BL47^{T}**	**1**	**+**	**+**	**FNHS01000014.1**	**FNHS01000008.1**	
		***M. platani* PMB02^{T}**	**3**	**+**	**+**	**LWHQ01000006.1**	**LWHQ01000004.1**	
		M. podarium DSM 15083^{T}	0					
		***M. populi* BJ001^{T}**	**3**	**+**	**+**	**CP001029.1**	**CP001029.1**	
		M. pseudosasae NBRC 105205^{T}	0					
		M. pseudosasicola BL36^{T}	1	+	-	FOTK01000006.1		
		***M. radiotolerans* JMC 2831^{T}**	**7**	**+**	**+**	**CP001001.1**	**CP001001.1**	
		M. lusitanum DSM 14457^{T}	0					
		M. rhodesianum DSM 5687^{T}	0					
		M. rhodinum JCM 2811^{T}	1	n.d.				
		***M. salsuginis* CGMCC 1.6474^{T}**	**1**	**+**	**+**	**FOSV01000002.1**	**FOSV01000017.1**	
		M. soli KCTC 22810^{T}	0					
		M. specialis	0					
		M. suomiense DSM 14458^{T}	0					
		M. tardum DSM 19566^{T}	0					
		***M. tarhaniae* DSM 25844^{T}**	**1**	**+**	**+**	**LABZ01000200.1**	**LABZ01000071.1**	
		M. thiocyanatum JCM 10893^{T}	1	n.d.				
		M. thuringiense LMG 25777^{T}	0					
		M. trifolii LMG 25778^{T}	0					
		***M. variabile* DSM 16961^{T}**	**1**	**+**	**+**	**LABY01000037.1**	**LABY01000026.1**	
		***M. zatmanii* PSBB041**	**1**	**+**	**+**		**CP021054..1**	
	Microvirga	*M. aerilata* NBRC 106137^{T}	0					
		M. aerophila NBRC 106136^{T}	0					
		M. arabica DSM 25393^{T}	0					
		M. floculans ATCC BAA-817^{T}	1	-	+		JAEA01000009.1	
		M. guangxiensis CGMCC 1.7666^{T}	1	-	+		FMVJ01000002.1	
		M. indica KACC 18792^{T}	0					
		M. lotononidis WSM3557^{T}	1	-	+		JH660647.1	
		M. lupini Lut6^{T}	1	n.m/n.d.				Reeve et al., 2014 (10.4056/sigs.5249382)
		M. makkahensis DSM 25394^{T}	0					
		***M. massiliensis* JC119^{T}**	**2**	**+**	**+**	**CAHM01000265.1**	**CAHM01000317.1**	
		M. oossetica LMG 29787^{T}	0					
		M. soli NBRC 112417^{T}	0					
		M. subterranea DSM 14364^{T}	0					
		M. vignae BR3299^{T}	1	-	+		LCYG01000014.1	
		M. zambiensis LMG 26454^{T}	0					
	Neomegalonema	*N. perideroedes* DSM 15528^{T}	1	n.d.				
	Psychroglaciecola	*P. arctica* KACC 17684^{T}	0					
Methylocystaceae	*Albibacter*	*A. helveticus* CIP 106788^{T}	0					
		A. methylovorans DSM 22840^{T}	0					
	Chthonobacter	*C. albigriseus* JCM 30603^{T}	0					

	Hansschlegelia	*H. beijingensis* DSM 25481^{T}	0					
		H. plantiphila NCIMB 14035^{T}	0					
		H. zhihuaiae DSM 18984^{T}	0					
	Methylocystis	*M. bryophila* S285	1	n.m./n.d.				Belova et al., 2013 (10.1099/ijs.0.043505-0)
		M. echinoides IMET 10491^{T}	0					
		M. heyeri DSM16984^{T}	0					
		M. hirsuta DSM 18500^{T}	0					
		M. methanolicus	0					
		M. minimus	0					
		M. parvus OBBP^{T}	1	-	+		AJTV01000013.1	
		M. pyriformis	0					
		M. rosea SV97^{T}	1	n.m./n.d.				Wartiainen et al., 2006 (10.1099/ijs.0.63912-0)
	Methylopila	*M. capsulata* DSM 6130^{T}	0					
		M. henanensis LMG 25959^{T}	0					
		M. jiangsuensis DSM 22718^{T}	0					
		M. musalis DSM 24986^{T}	0					
		M. oligotropha CCUG 63805^{T}	0					
		M. turkensis DSM 27566^{T}	0					
	Methylosinus	*M. acidophilus*	0					
		M. pucelana	0					
		M. sporium ATCC 35069^{T}	0					
		M. trichosporium OB3b^{T}	1	-	+		ADVE02000001.1	
	Methylosporovibrio	*M. methanica*	0					
	Oharaeibacter	*O. diazotrophicus* DSM 102969^{T}	0					
	Pleomorphomonas	*P. diazotrophica* DSM 25022^{T}	0					
		P. koreensis DSM 23070^{T}	1	+	-	AULH01000007.1		
		***P. oryzae* DSM 16300^{T}**	**1**	**+**	**+**	**AUHB01000012.1**	**AUHB01000003.1**	
	Terasakiella	*T. brassicae* KCTC 42652^{T}	0					
		T. pusilla DSM 6293^{T}	1	+	-	JHYO01000010.1		
Phyllobacteriaceae	*Aliihoeflea*	*A. aestuarii* DSM 19536^{T}	0					
	Aminobacter	*A. aganoensis* DSM 7051^{T}	0					
		A. aminovorans KCTC 2477	1	-	+		CP015005.1	
		A. anthyllidis LMG 26462^{T}	0					
		A. ciceronei ATCC 202197^{T}	0					
		A. lissarensis NCIMB 13789^{T}	0					
		A. niigataensis DSM 7050^{T}	0					
	Aquamicrobium	*A. aerolatum* DSM 21857^{T}	1	n.d.				
		A. aestuarii JCM 16876^{T}	0					
		A. ahrensii DSM 19730^{T}	0					
		A. defluvii W13Z1	1	-	+		JENY01000006.1	
		A. lusatiense DSM 11099^{T}	0					
		A. segne DSM 18714^{T}	0					
		A. soli KCTC 52165^{T}	0					
		A. terrae DSM 27865^{T}	0					
	Chelativorans	*C. composti* JCM 17863^{T}	0					
		C. intermedius DSM 29391^{T}	0					
		C. multitrophicus DSM 9103^{T}	0					
		C. oligotrophicus DSM 19276^{T}	0					
	Corticibacterium	*C. populi* KCTC 42249^{T}	0					
	Hoeflea	*H. alexandrii* DSM 16655^{T}	0					
		H. anabaenae CCUG 56626^{T}	0					
		H. halophila JCM 16715^{T}	0					
		H. marina ATCC 25654^{T}	0					
		H. olei JC234^{T}	1	-	+		LQZT01000042.1	

	H. phototrophica DFL-43^T	1	-	+	CM002917.1
	H. siderophila	0			
Lentilitoribacter	*L. donghaensis* KCTC 32082^T	0			
Mesorhizobium	*M. abyssinicae* LMG 26967^T	0			
	M. acaciae JCM 30534^T	0			
	M. albiziae DSM 21822^T	0			
	M. alhagi CCNWXJ12-2^T	1	-	+	AHAM01000061.1
	M. amorphae CCNWGS0123	3	-	+	CP015318.1
	M. australicum WSM2073^T	2	-	+	CP003358.1
	M. calcicola LMG 28224^T	0			
	M. camelthorni ATCC 14549^T	0			
	M. cantuariense LMG 28225^T	0			
	M. caraganae LMG 24397^T	0			
	M. chacoense DSM 17287^T	0			
	M. ciceri WSM1271	6	-	+	CP002447.1
	M. delmonti LMG 29640^T	0			
	M. erdmanii USDA 3471^T	1	-	+	KI421455.1
	M. gobiense LMG 23949^T	0			
	M. hawassense LMG 26968^T	0			
	M. hemanticense LMG 29734^T	0			
	M. huakuii 7653R	1	-	+	CP006581.1
	M. japonicum MAFF303099	2	-	+	BA000012.4
	M. jarvisii ATCC 33669^T	0			
	M. kowhaii LMG 28222^T	0			
	M. loti NZP2037	20	-	+	CP016079.1
	M. mediterraneum USDA 3392	1	n.d.		
	M. metallidurans STM 2683^T	1	n.d.		
	M. muleiense CGMCC 1.11022^T	1	n.d.		
	M. newzealandense LMG 28226^T	0			
	M. olivaresii LMG 29295^T	0			
	M. opportunistum WSM2075^T	1	-	+	NC_015675.1
	M. parafortuitum	0			
	M. plurifarium ORS 1032^T	4	-	+	CCND01000017.1
	M. prunaredense 1	1	n.d.		
	M. qingshengii CGMCC 1.12097^T	1	-	+	FMXM01000006.1
	M. robinae ACCC 14543^T	0			
	M. sangaii ACCC 13218^T	0			
	M. sediminum KCTC 42205^T	0			
	M. septentrionale LMG 23930^T	0			
	M. shangrilense LMG 24762^T	0			
	M. shonense LMG 26966^T	0			
	M. silamurunense LMG 24822^T	0			
	M. soli JCM 19897^T	0			
	M. sophorae ICMP 19535^T	1	-	+	NNRI01000008.1
	M. tamadayense LMG 26736^T	0			
	M. tarimense LMG 24338^T	0			
	M. temperatum SDW018^T	1	n.d.		
	M. thiogangeticum LMG 22697^T	0			
	M. tianshaense DSM 11417^T	0			
	M. waimense LMG 28228^T	0			
	M. waitaakense LMG 28227^T	0			
Nitratireductor	*N. aestuarii* LMG 29090^T	0			
	N. aquibiodomus JCM 21793^T	3	-	+	BAMP01000001.1
	N. aquimarinus JCM 17288^T	0			

		N. basaltis RR3-28	1	-	+	CP019044.1	
		N. indicus C115^{T}	2	-	+	AMSI01000010.1	
		N. kimnyeongensis JCM 14851^{T}	0				
		N. lacus KCTC 52186^{T}	0				
		N. lucknowense DSM 24322^{T}	0				
		N. pacificus pht-3B^{T}	1	-	+	AMRM01000029.1	
		N. shengliensis LMG 27405^{T}	0				
		N. soli ZZ-1^{T}	1	-	+	LFVY01000010.1	
	Oricola	*O. cellulosilytica* JCM 19534^{T}	0				
	Paramesorhizobium	*P. deserti* A-3-E^{T}	1	-	+	LNTU01000034.1	
	Phyllobacterium	*P. bourgognense* LMG 22837^{T}	0				
		P. brassicacearum LMG 22836^{T}	0				
		P. catacumbae LMG 22520^{T}	0				
		P. endophyticum LMG 26470^{T}	0				
		P. ifriqiyense LMG 22831^{T}	0				
		P. laguminum LMG 22833^{T}	0				
		P. loti LMG 27289^{T}	0				
		P. myrsinacearum DSM 5892^{T}	0				
		P. sophorae LMG 27899^{T}	0				
		P. trifolii LMG 22712^{T}	0				
	Pseudahrensia	*P. aquimaris* KCTC 23345^{T}	0				
		P. todarodis JCM 30419^{T}	0				
	Pseudaminobacter	*P. defluvii* NBRC 14570^{T}	0				
		P. manganicus JH-7^{T}	1	-	+	MDET01000003.1	
		P. salicylatoxidans KCT001	1	-	+	CAIU01000025.1	
	Pseudohoeflea	*P. suedae* NBRC 107700^{T}	0				
	Roseitalea	*R. porphyridii* JCM 31538^{T}	0				
	Thalassocella	*T. subtropicalis*	0				
	Thalassocola	*T. ureilytica* JCM 30682^{T}	0				
	Tianweitania	*T. sediminis* JCM 30358^{T}	0				
Rhizobiaceae	*Amorphomonas*	*A. oryzae*	0				
	Carbophilus	*C. carboxidus* DSM 1086^{T}	0				
	Ciceribacter	*C. lividus* DSM 25528^{T}	0				
		C. thiooxidans KCTC 5223^{T}	0				
	Kaistia	*K. adipata* DSM 17808^{T}	1	n.m./n.d.			Im et al., 2004 (10.2323/jgam.50.249)
		K. dalseonensis DSM 18800^{T}	0				
		K. defluvii JCM 18034^{T}	0				
		K. geumhonensis DSM 18799^{T}	0				
		K. granuli DSM 23481^{T}	1	n.m./n.d.			Lee et al., 2007 (10.1099/ijs.0.65023-0)
		K. hirudinis LMG 26925^{T}	0				
		K. soli DSM 19436^{T}	1	n.m./n.d.			Weon et al., 20088 (10.1099/ijs.0.65366-0)
		K. terrae DSM 21341^{T}	0				
	Liberibacter[d]	*L. africanus* PTSAPSY	1	-	+	CP004021.1	
		L. americanus Sao Paulo	2	-	+	CP006604.1	
		L. asiaticus psy62	10	-	+	CP001677.5	
		L. crescens BT-1^{T}	2	-	+	CP003789.1	
		L. europaeus	0				
		L. psyllaorus	0				
		L. solanacearum CLso-ZC1	7	-	+	CP002371.1	
	Agrobacterium	*A. agile*	0				
		A. albertimagni AOL15	1	-	+	ALJF01000010.1	
		A. arsenijevicii KFB 330^{T}	1	-	+	JWIT01000016.1	
		A. aurantiacum	0				
		A. fabrum C58^{T}	2	-	+	AE007869.2	

	A. larrymoorei ATCC 51759^T	1	n.d.		
	A. rhizogenes K599	9	-	+	CP019701.1
	A. rubi NBRC 13261^T	2	n.d.		
	A. salinitolerans YIC 5082^T	1	-	+	MRDH01000013.1
	A. tumefaciens Ach5	38	-	+	CP011246.1
	A. viscosum	0			
	A. vitis S4	13	-	+	CP000633.1
Neorhizobium	*N. alkalisoli* LMG 24763^T	0			
	N. galegae HAMBI 540^T	10	-	+	HG938353.1
	N. huautlense LMG 18254^T	0			
Pararhizobium	*P. antarcticum* 59	1	-	+	LSRP01000094.1
	P. capsulatum DSM 1112^T	0			
	P. polonicum F5.1^T	1	-	+	LGLV01000018.1
Pseudorhizobium	*P. pelagicum* R1-200B4^T	2	-	+	JOKI01000001.1
Rhizobium	*R. acidisoli* FH23	1	-	+	LJSR01000011.1
	R. aegyptiacum 950	1	n.d.		
	R. aethiopicum HBR26^T	1	-	+	FMAJ01000014.1
	R. aggregatum DSM 1111^T	0			
	R. alamii YR540	2	-	+	JQMB01000017.1
	R. alvei LMG 26895^T	0			
	R. anhuiense WYCCWR10015	1	-	+	LWMO01000062.1
	R. arachis	0			
	R. arenae MIM27^T	1	-	+	MOOY01000008.1
	R. azibense CCBAU 10108^T	0			
	R. azooxidifex DSM 100211^T	0			
	R. bangladeshense 1017	3	-	+	LYPK01000011.1
	R. binae DSM 29288^T	0			
	R. borbori LMG 23925^T	0			
	R. calliandrae CIP 110456^T	0			
	R. capsici JCM 19535^T	0			
	R. cauense LMG 26832^T	0			
	R. cellulosilyticum DSM 18291^T	0			
	R. cnuense	0			
	R. daejeonense NBRC 102495^T	0			
	R. ecuadorense CNPSO 671^T	1	-	+	LFIO01000958.1
	R. endolithicum KCTC 32077^T	0			
	R. endophyticum LMG 26002^T	0			
	R. esperanzae CNPSo 668^T	1	-	+	MXPU01000010.1
	R. etli CFN 42^T	11	-	+	CP000133.1
	R. fabae LMG 23997^T	0			
	R. favelukesii LPU83^T	2	-	+	HG916852.1
	R. flavum YW14^T	1	-	+	MUXO01000046.1
	R. freirei PRF 81^T	1	-	+	AQHN01000053.1
	R. gallicum R602^T	3	-	+	CP006877.1
	R. gei LMG 27603^T	0			
	R. giardinii H152^T	1	-	+	KB902578.1
	R. grahamii CCGE 502^T	1	-	+	AEYE02000004.1
	R. hainanense CCBAU 57015^T	1	-	+	FMAC01000008.1
	R. halophytocola DSM 21600^T	0			
	R. halotolerans JCM 17536^T	0			
	R. helanshanense HAMBI 3083^T	0			
	R. helianthi KCTC 23879^T	0			
	R. herbae LMG 25718^T	0			
	R. indigoferae NBRC 100398^T	0			

R. ipomoeae LMG 27163T	0			
R. jaguaris CIP 110453T	0			
R. kunmingense KCTC 22609T	0			
R. laguerreae FB206T	1	-	+	MRDM01000010.1
R. leguminosarum WSM1689	42	-	+	CP007045.1
R. lemnae NBRC 109339T	0			
R. lentis DSM 29286T	0			
R. leucaenae USDA 9039T	2	-	+	AUFB01000003.1
R. loessense CGMCC 1.3401	1	-	+	FMTM01000001.1
R. lusitanum P1-7T	1	-	+	FMAF01000013.1
R. marinum MGL06T	1	-	+	JMQK01000002.1
R. mayense CIP 110454T	0			
R. mesoamericanum STM3625	2	-	+	HF536772.1
R. mesosinicum LMG 24135T	0			
R. metallidurans DSM 26575T	0			
R. miluonense HAMBI 2971T	1	-	+	FMAH01000020.1
R. mongolense USDA 1844T	1	-	+	ATTQ01000014.1
R. multihospitium HAMBI 2975T	1	-	+	FMAG01000002.1
R. naphthalenivorans NBRC 107585T	0			
R. nepotum 37/9T	1	-	+	JWJH01000014.1
R. oryzae CGMCC 1.7048T	3	-	+	FOAM01000013.1
R. oryzicola KCTC 32088T	0			
R. oryziradicis N19T	1	-	+	MKIM01000024.1
R. pakistanense LMG 27895T	0			
R. paknamense NBRC 109338T	0			
R. paranaense LMG 27577T	0			
R. petrolearium DSM 21882T	0			
R. phaseoli N261	16	-	+	CP013580.1
R. pisi DSM 30132T	0			
R. pongamiae	0			
R. populi JCM 19159T	0			
R. pseudoryzae KCTC 23294T	0			
R. puerariae NBRC 110722T	0			
R. pusense LMG 25623T	4	-	+	FNBB01000001.1
R. qilianshanense JCM 18337T	0			
R. rhizoryzae KCTC 23652T	0			
R. rhizosphaerae MH17T	2	-	+	MKIO01000030.1
R. rosettiformans MTCC 9454T	0			
R. selenitireducens ATCC BAA-1503T	1	-	+	KI912495.1
R. skierniewicense LMG 26191T	0			
R. smilacinae LMG 27604T	0			
R. soli JCM 14591T	0			
R. sophorae CCBAU 03386T	1	-	+	MRDK01000043.1
R. sophoriradicis LMG 27898T	0			
R. sphaerophysae ACCC 17498T	0			
R. straminoryzae JCM 19536T	0			
R. subbaraonis DSM 24765T	0			
R. sullae WSM1592T	2	-	+	ATZB01000008.1
R. taeanense	0			
R. taibaishanense ACCC 14971T	1	-	+	MKIN01000016.1
R. tarimense CCTCC AB 2011011T	0			
R. tibeticum CGMCC 1.7071T	2	-	+	FOCV01000001.1
R. tropici CIAT 899T	5	-	+	CP004015.1
R. tubonense LMG 25225T	0			

		R. undicola ATCC 700741^{T}	1	-	+	JHXQ01000003.1
		R. vallis LMG 25295^{T}	0			
		R. vignae CCBAU 05176^{T}	1	-	+	JNNU01000014.1
		R. viscosum DSM 7307^{T}	0			
		R. wenxiniae DSM 100734^{T}	0			
		R. yanglingense CCBAU 01603	1	-	+	JHUO01000002.1
		R. yantingense LMG 28229^{T}	0			
		R. zeae LMG 29735^{T}	0			
	Shinella	*S. daejeonensis* JCM 16236^{T}	0			
		S. fusca LMG 24714^{T}	0			
		S. granuli JCM 13254^{T}	0			
		S. kummerowiae LMG 24136^{T}	0			
		S. yambaruensis DSM 18801^{T}	0			
		S. zoogloeoides ATCC 19623^{T}	0			
	Ensifer	*E. adhaerens* OV14	6	-	+	CP007236.1
		E. aridi TW10	6	-	+	AZNX01000001.1
		E. garamanticus LMG 24692^{T}	0			
		E. glycinis CCBAU 23380^{T}	1	-	+	LPUX01000053.1
		E. maghrebium	0			
		E. mexicanus DSM 18446^{T}	0			
		E. numidicus LMG 24690^{T}	0			
		E. psoraleae LMG 26835^{T}	0			
		E. sesbaniae LMG 26833^{T}	0			
		E. shofinae CCBAU 251167^{T}	1	-	+	MBFK01000100.1
		E. sojae CCBAU 05684^{T}	2	-	+	CP023067.1
		E. xericitae	0			
	Sinorhizobium	*S. abri*	0			
		S. americanum CCGM7	3	-	+	CP013051.1
		S. arboris LMG 14919^{T}	1	-	+	ATYB01000014.1
		S. chiapanecum ITTG S70^{T}	0			
		S. fredii CCBAU 83666^{T}	13	-	+	CP023070.1
		S. idiaense	0			
		S. kostiense DSM 13372^{T}	0			
		S. kummerowiae NBRC 100799^{T}	0			
		S. medicae WSM419	5	-	+	CP000738.1
		S. meliloti 1021	46	-	+	AL591688.1
		S. saheli LMG 7837^{T}	1	-	+	LNQB01000073.1
		S. terrangae DSM 11282^{T}	0			
		S. xinjiangense DSM 5852^{T}	0			
Roseiarcaceae	*Roseiarcus*	*R. fermentans* DSM 24875^{T}	0			
Xanthobacteraceae	*Ancylobacter*	*A. abiegnus* Z-0056^{T}	0			
		A. aquaticus DSM 101^{T}	0			
		A. defluvii CCUG 63806^{T}	0			
		A. dichloromethanicus DSM 21507^{T}	0			
		A. oerskovii DSM 18746^{T}	0			
		A. polymorphus DSM 2457^{T}	0			
		A. pratisalsi DSM 102029^{T}	0			
		A. rudongensis CGMCC 1.1761	1	-	+	FMTP01000009.1
		A. sunchi JCM 32039^{T}	0			
		A. vacuolatus DSM 1277^{T}	0			
	Azorhizobium	*A. caulinodans* ORS 571^{T}	1	-	+	NC_009937.1
		A. deobereinerae UFLA1-100^{T}	1	-	+	AXBA01000031.1
		A. oxalatiphilum DSM 18749^{T}	0			
	Labrys	*L. gingengisoli*	0			

		L. methylaminiphilus DSM 16812[T]	0					
		L. miyagiensis NBRC 101365[T]	0					
		L. monachus DSM 5896[T]	0					
		L. neptuniae LMG 23578[T]	0					
		L. okinaaawensis DSM 18385[T]	0					
		L. oportucalensis DSM 17916[T]	0					
		L. soli JCM 19895[T]	0					
		L. wisconsinensis DSM 19619[T]	0					
	Pseudolabrys	*P. taiwanensis* CIP 108932[T]	0					
	Pseudoxanthobacter	*P. liyangensis* KACC 16601[T]	0					
		P. soli DSM 19599[T]	1	-	+		FRXO01000002.1	
	Starkeya	*S. koreensis* JCM 21669[T]	0					
		S. novella DSM 506[T]	1	n.m./n.d.				Kappler et al., 2012 (10.4056/sigs.3006378)
	Xanthobacter	*X. agilis* DSM 3770[T]	0					
		X. aminoxidans DSM 15009[T]	0					
		X. autotrophicus Py2[T]	1	n.d.				
		X. flavus DSM 338[T]	0					
		X. polyaromaticivorans 127W[T]	0					
		X. tagetidis DSM 11105[T]	0					
		X. viscosus ATCC BAA-298[T]	0					
		X. xylophilus	0					
unclassified *Rhizobiales*	*Alsobacter*	*A. metallidurans* NBRC 107718[T]	0					
	Bauldia	*B. consociata* CCM 7594[T]	0					
		B. litoralis ATCC 35022[T]	1	n.d.				
	Endowatersipora [d]	*E. palomitas*	0					
		E. rubus	0					
	Hodgkinia [d]	*E. cicadicola* Dsem	5	n.d.				
	Tokpelaia [d]	*T. hoelldoblerii* Hsal	1	-	+		CP017315	
	Hartmannibacter	*H. diazotrophicus* LMG 27460[T]	0					
	Methylobrevis	*M. pamukkalensis* PK2[T]	1	+	-		MCRJ01000144.1	
	Methyloceanibacter	*M. caenitepidi* Gela4[T]	1	n.m./n.d.				Takeuchi et al., 2014 (10.1099/ijs.0.053397-0)
		M. marginalis R-67177[T]	1	n.d.				
		M. methanicus R-67174[T]	1	n.d.				
		M. stevinii R-67176[T]	1	n.d.				
		M. superfactus R-67175[T]	1	n.d.				
	Methyloligella	*M. halotolerans* VKM B-2706[T]	1	n.m./n.d.				Doroniva et al., 2013 (10.1016/j.syapm.2012.12.001)
		M. solikamskensis DSM 25212[T]	0					
	Methylosulfonomonas	*M. methylovora*	0					
	Nordella	*N. oligomobilis* CIP 106862[T]	0					
	Pseudorhodoplanes	*P. sinuspersici* RIPI 110[T]	1	+	-	CP021112		
	Qingshengfania	*Q. soli* KCTC 42463[T]	0					
	Reichenowia	*R. ornatae*	0					
		R. parasiticae	0					
		R. pictae	0					
	Solomonas	*S. fluorantheni*	0					

Taxonomy according to NCBI taxonomy database, accessed in October 2017. Species with no sequenced genome are highlighted in red typing. Genomes harboring both, the subpolar and lateral flagellar systems are highlighted in bold. [T] indicates type strain.

[a]Only sequenced genomes of type strains and/or high-quality genomes are considered. Unclassified isolates and candidatus species are not considered.

[b]Reviewed in October, 2017 from the NCBI genome database

[c]Genome downloaded from the JGI IMG.

[d]*Candidatu* s genera.

n.d. Genes for flagellar systems not detected in genome annotations.

n.m. Non-motile bacteria.
