# Supplementary material for: Phylogenomic Analyses of Bradyrhizobium Reveal Uneven Distribution of the Lateral and Subpolar Flagellar Systems, Which Extends to Rhizobiales
Source: Microorganisms. 2019 Feb 13;7(2):50. doi: 10.3390/microorganisms7020050 (PMC6406911; doi:10.3390/microorganisms7020050)
Supplement: Supplementary file 1 [file microorganisms-07-00050-s001.zip › Supplementary_Figure_S2.pdf]

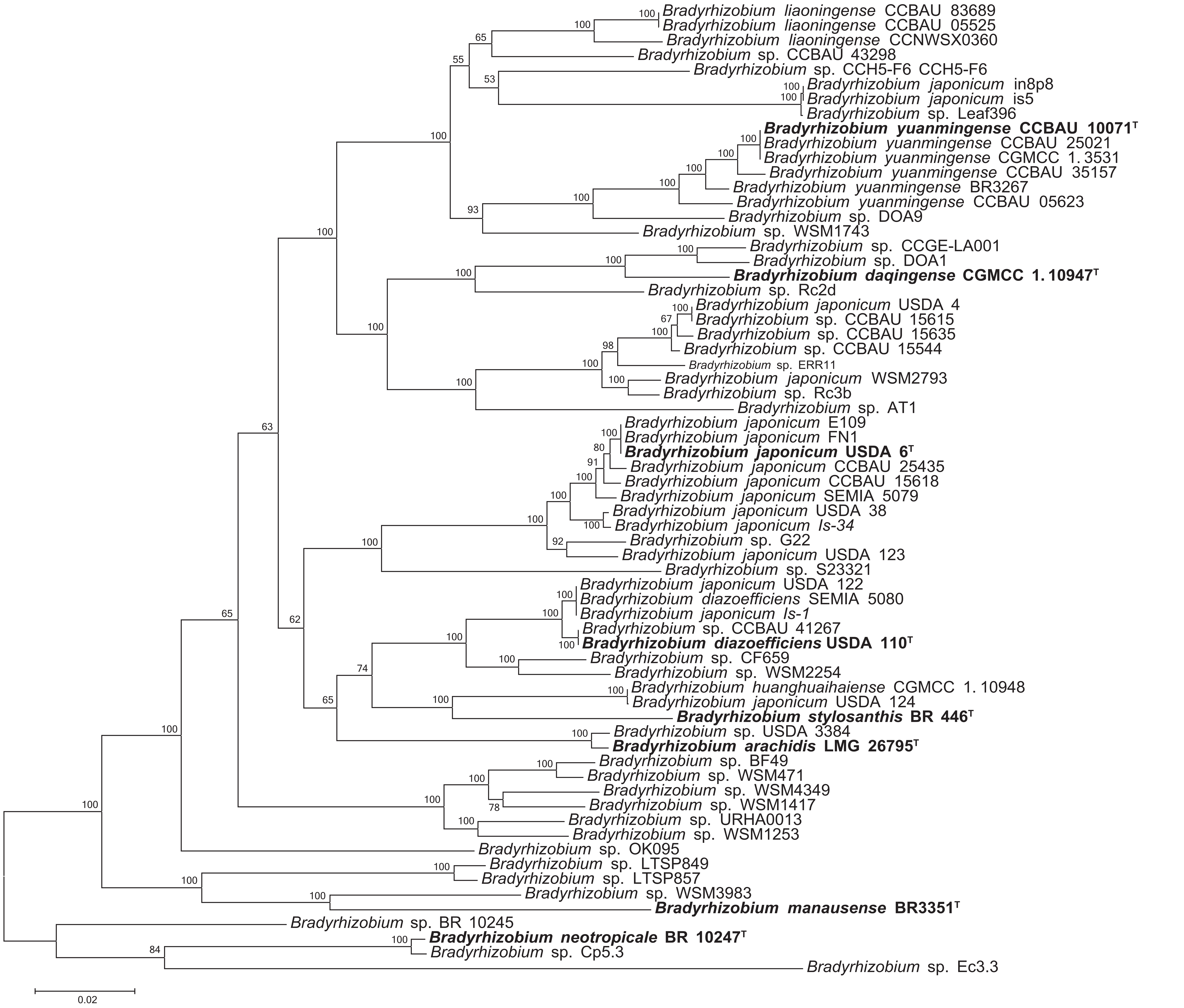

Supplementary Figure S2. Unrooted ML tree based on the concatenated sequences of 35 lateral flagellum CDSs (*lafR*, bll6847, *flgN*, *flgJ*, *fliR*, *flhA*, *fliQ*, *flgD*, *flbT*, *flaF*, *flgL*, *flgK*, *flgE*, *fliK*, *motB*, bll6863, *fliF*, *fliL*, *flgH*, bll6870, *flgI*, *flgA*, *flgG*, *fliE*, *flgC*, *flgB*, *flhB*, *fliG*, *fliN*, bll6880, *fliM*, *motA*, *flgF1*, *fliI* and bll6886 as in *B. diazoefficiens* USDA 110^T genome BA000040.2 from position 7,544,342 to 7,577,700) in 66 *Bradyrhizobium* genomes. Bold and ^{T} indicate type strain.
