# Supplementary material for: Phylogenomic Analyses of Bradyrhizobium Reveal Uneven Distribution of the Lateral and Subpolar Flagellar Systems, Which Extends to Rhizobiales
Source: Microorganisms. 2019 Feb 13;7(2):50. doi: 10.3390/microorganisms7020050 (PMC6406911; doi:10.3390/microorganisms7020050)
Supplement: Supplementary file 1 [file microorganisms-07-00050-s001.zip › Supplementary_Table_S1.pdf]

Supplementary Table S1. General genomic features of genomes used in this study.

Name	Assembly NCBI ID / IMG Genome ID	RefSeq Ascc. No.	Scaffolds	Size (Mb)	GC (%)	Genes	Proteins
Afipia birgiae 34632[T]	GCA_000308295.2	CAJQ02	65	5.33	60.8	5166	4871
Afipia broomeae ATCC 49717[T]	GCA_000314675.2	AGWX01	4	5.26	61.3	5086	4953
Afipia clevelandensis ATCC 49720[T]	GCA_000336555.1	AGWY01	19	4.4	61.7	4248	4151
Agrobacterium fabrum C58[T]	GCA_000092025.1	NC_003062.2<br>NC_003063.2	-	5.67	59.1	5459	5355
Agrobacterium sp. H13-3	GCA_000192635.1	NC_015183.1<br>NC_015508.1	-	5.57	58.5	5365	5154
Agrobacterium tumefaciens Ach5	GCA_000971565.1	NZ_CP011246.1<br>NZ_CP011247.1	-	5.67	58.5	5404	5184
Agrobacterium tumefaciens ATCC 31700	GCA_001975795.1	MTKI01	117	5.94	58.4	5709	5462
Aminobacter aminovorans KCTC 2477	GCA_001605015.1	NZ_CP015005.1	-	5.62	63.4	5542	5362
Aquamicrobium aerolatum DSM 21857[T]	GCA_900113935.1	FORF01	50	3.64	60.1	3573	3513
Aquamicrobium defluvii W13Z1	GCA_000585625.1	JENY01	65	4.77	63.1	4703	4455
Aurantimonas coralicida DSM 14790[T]	GCA_000421645.1	ATXK01	38	4.62	66.7	4359	4198
Aurantimonas manganoxydans DSM 21871[T]	GCA_001463865.1	BBWP01	94	4.25	66.8	3944	3821
Aureimonas altamirensis DSM 21988[T]	GCA_900141975.1	FQZC01	11	4.19	64.8	3983	3894
Aureimonas frigidaquae JCM 14755[T]	GCA_001463905.1	BBWR01	30	4.10	66.1	3864	3774
Aureimonas jatrophae DSM 25025[T]	GCA_900104035.1	FNIT01	27	4.59	67.8	4360	4304
Aureimonas phyllosphaerae DSM 25026[T]	GCA_900113065.1	FOOA01	62	4.72	67.5	4538	4479
Aureimonas ureilytica DSM 18598[T]	GCA_000382705.1	ARQE01	44	5.09	67.3	4792	4637
Bartonella ancashensis 20.00[T]	GCA_001281405.1	NZ_CP010401.1	-	1.47	38.4	1317	1203
Bartonella bacilliformis ATCC 35685[T]	GCA_001559035.1	NZ_CP014012.1	-	1.44	38.2	1284	1183
Bartonella bovis 91-4[T]	GCA_000384965.1	NZ_CM001844.1	1	1.63	37.4	2809	2627
Bartonella clarridgeiae ATCC 51734[T]	GCA_000518185.1	JADC01	17	1.49	35.7	1275	1170
Bartonella schoenbuchensis R1[T]	GCA_002022685.1	NZ_CP019789.1	2	1.73	37.8	1587	1330
Beijerinckia indica ATCC 9039[T]	GCA_000019845.1	NC_010581.1	-	4.17	57.1	3767	3619
Beijerinckia mobilis UQM 1969[T]	GCA_000745425.1	JQJH01	58	4.32	57.2	-	3680
Blastochloris viridis ATCC 19567	GCA_001402875.1	NZ_CP012946.1	-	3.72	67.9	3265	3157
Blastochloris viridis DSM 133[T]	GCA_001548155.2	NZ_AP014854.2	-	3.73	67.9	3260	3149
Bosea robinae DSM 26672[T]	GCA_900102525.1	FNBZ01	21	5.28	66.3	5067	5006
Bosea vaviloviae Vaf-18[T]	GCA_001741865.1	NZ_CP017147.1	-	6.71	65.4	6271	6105
Bradyrhizobium arachidis LMG 26795	GCA_900116675.1	FPBQ01	98	9.79	63.6	9374	9312

Bradyrhizobium canariense GAS369	GCA_900105125.1	LT629750.1	-	7.84	60.9	7549	7291
Bradyrhizobium daqingense CGMCC 1.10947[a]	2596583556	-	107	7.89	63.7	7559	7480
Bradyrhizobium diazoefficiens NK6	GCA_001549695.1	AP014685.1	-	10.5	63.6	10249	8598
Bradyrhizobium diazoefficiens SEMIA 5080	GCA_000648595.2	ADOU02	13	9.09	64.0	8726	8252
Bradyrhizobium diazoefficiens USDA 110	GCA_000011365.1	BA000040.2	-	9.11	64.1	8373	8317
Bradyrhizobium diazoefficiens USDA 122	GCA_000473025.1	AXAX01	101	8.98	64.0	8673	8184
Bradyrhizobium elkanii 587	GCA_000257685.2	AJJK01	2430	8.67	63.6	-	-
Bradyrhizobium elkanii BLY3-8	GCA_001718205.1	LWUI01	90	9.20	63.8	8553	8143
Bradyrhizobium elkanii BLY6-1	GCA_001718185.1	LXEM01	79	9.20	63.8	8549	8148
Bradyrhizobium elkanii CCBAU 05737	GCA_000261505.1	AJPV01	751	9.77	63.5	9578	8527
Bradyrhizobium elkanii CCBAU 43297	GCA_000261525.1	AJPW01	654	9.35	63.7	9092	8193
Bradyrhizobium elkanii UASWS1015	GCA_000878305.1	JXOF01	187	7.82	64.6	7306	7077
Bradyrhizobium elkanii USDA 3254	GCA_000472765.1	AXAH01	85	8.98	63.8	8497	7977
Bradyrhizobium elkanii USDA 3259	GCA_000473005.1	AXAW01	101	8.72	63.9	8264	7782
Bradyrhizobium elkanii USDA 76	GCA_000379145.1	ARAG01	2	9.49	63.7	8969	8534
Bradyrhizobium elkanii USDA 94	GCA_000519225.1	JAFC01	164	9.56	63.7	9038	8504
Bradyrhizobium elkanii WSM1741	GCA_000472965.1	AXAU01	69	7.95	62.1	7520	7116
Bradyrhizobium elkanii WSM2783	GCA_000472865.1	AXAP01	217	9.90	62.4	9510	8824
Bradyrhizobium embrapense SEMIA 6208	GCA_001189235.2	LFIP02	36	8.27	64.0	7729	7404
Bradyrhizobium erythrophlei GAS138	GCA_900129425.1	LT670817.1	-	9.09	61.4	8638	8280
Bradyrhizobium erythrophlei GAS242	GCA_900129505.1	LT670818.1	-	9.19	61.9	8690	8320
Bradyrhizobium erythrophlei GAS401	GCA_900142985.1	LT670849.1	-	7.53	61.2	7214	7027
Bradyrhizobium erythrophlei MT12	GCA_900105845.1	FNTH01	2	8.97	63.8	8417	8054
Bradyrhizobium huanghuaihaiense CGMCC 1.10948[a]	2596583587	-	101	9.23	63.9	8760	8654
Bradyrhizobium icense LMTR 13	GCA_001693385.1	CP016428.1	-	8.32	62.0	7868	7456
Bradyrhizobium japonicum 22	GCA_000482425.1	AXVG01	4	7.51	64.5	7121	6860
Bradyrhizobium japonicum CCBAU 15354	GCA_000261545.1	AJPX01	951	10.1	63.3	10250	8898
Bradyrhizobium japonicum CCBAU 15517	GCA_000261565.1	AJPY01	1129	9.92	63.4	10050	8694
Bradyrhizobium japonicum CCBAU 15618	GCA_000261585.1	AJPZ01	691	9.82	63.4	9846	8828
Bradyrhizobium japonicum CCBAU 25435	GCA_000261605.1	AJQA01	520	9.46	63.5	9292	8475
Bradyrhizobium japonicum CCBAU 83623	GCA_000261625.1	AJQB01	1212	10.1	63.3	10277	8854
Bradyrhizobium japonicum E109	GCA_000807315.1	CP010313.1	-	9.22	63.7	8741	8404
Bradyrhizobium japonicum FN1	GCA_001038185.1	JGCL01	87	9.14	63.7	8664	8320
Bradyrhizobium japonicum in8p8	GCA_000426845.1	AUGD01	52	7.59	63.8	7270	6947
Bradyrhizobium japonicum Is-1	GCA_001280585.1	LGUJ01	104	8.98	64.0	8656	8201
Bradyrhizobium japonicum Is-34	GCA_000773865.1	JRPN01	248	10.3	63.0	9921	9376
Bradyrhizobium japonicum is5	GCA_000421305.1	ATWP01	60	7.59	63.8	7257	6929
Bradyrhizobium japonicum SEMIA 5079	GCA_000661935.1	CP007569.1	-	9.58	63.5	9143	8675

Bradyrhizobium japonicum USDA 123	GCA_000482525.1	AXVP01	517	10.6	63.3	10272	9181
Bradyrhizobium japonicum USDA 124	GCA_000374205.1	ARFJ01	123	9.00	64.0	8593	8182
Bradyrhizobium japonicum USDA 135	GCA_000472945.1	AXAT01	547	7.70	64.0	7662	6704
Bradyrhizobium japonicum USDA 38	GCA_000472745.1	AXAG01	107	9.61	63.5	9225	8760
Bradyrhizobium japonicum USDA 4	GCA_000472725.1	AXAF01	97	8.74	63.7	8235	7778
Bradyrhizobium japonicum USDA 6	GCA_000472985.1	AXAV01	87	9.13	63.7	8715	8321
Bradyrhizobium jicamae PAC68	GCA_001440395.1	LLXZ01	235	8.74	62.2	8277	7745
Bradyrhizobium lablabi GAS522	GCA_900104755.1	FNTI01	2	8.27	62.3	7822	7504
Bradyrhizobium liaoningense CCBAU 05525	GCA_000261645.1	AJQC01	1040	8.16	63.8	8111	6957
Bradyrhizobium liaoningense CCBAU 83689	GCA_000261665.1	AJQD01	1133	8.01	63.8	7966	6843
Bradyrhizobium liaoningense CCNWSX0360	GCA_001595995.1	LUKO01	242	8.59	63.7	8155	7651
Bradyrhizobium manausense BR3351	GCA_001440035.1	LJYG01	127	9.15	62.8	8404	7794
Bradyrhizobium neotropicale BR 10247	GCA_001641695.1	LSEF01	125	8.68	63.6	7989	7682
Bradyrhizobium oligotrophicum S58	GCA_000344805.1	AP012603.1	-	8.26	65.1	7291	7061
Bradyrhizobium ottawaense GAS524	GCA_900099825.1	LT629693.1	-	8.34	62.3	7956	7624
Bradyrhizobium pachyrhizi BR3262	GCA_001440015.1	LJYE01	116	8.97	63.7	8290	7621
Bradyrhizobium pachyrhizi PAC 48	GCA_001189245.1	LFIQ019.09	124	8.71	63.8	8096	7479
Bradyrhizobium paxllaeri LMTR 21	GCA_001693515.1	MAXB01	146	8.29	62.5	7713	7430
Bradyrhizobium retamae Ro19	GCA_001440415.1	LLYA01	235	8.47	61.9	7995	7481
Bradyrhizobium sp. 17-4	GCA_001312945.1	BBEX01	2463	7.11	64.5	-	-
Bradyrhizobium sp. Ai1a-2	GCA_000426245.1	AUEZ01	246	9.03	62.6	8411	7810
Bradyrhizobium sp. ARR65	GCA_000472385.1	AWZU01	139	8.61	62.0	8126	7603
Bradyrhizobium sp. AT1	GCA_001590795.1	JXDL01	8	7.54	64.2	7029	6673
Bradyrhizobium sp. BF49[a]	2751185896	-	1	7.55	63.8	7398	7328
Bradyrhizobium sp. BR 10245	GCA_001641635.1	LUUB01	133	10.1	63.2	9486	8827
Bradyrhizobium sp. BR 10303	GCA_001542415.1	LNCU01	137	8.72	63.2	8162	7593
Bradyrhizobium sp. BTAi1	GCA_000015165.1	CP000494.1	-	8.49	64.8	7716	7496
Bradyrhizobium sp. CCBAU 15544	GCA_000261705.1	AJQF01	612	8.48	63.7	8340	7529
Bradyrhizobium sp. CCBAU 15615	GCA_000261725.1	AJQG01	453	8.85	63.7	8600	7815
Bradyrhizobium sp. CCBAU 15635	GCA_000261745.1	AJQH01	456	8.29	63.8	8091	7410
Bradyrhizobium sp. CCBAU 41267	GCA_000261765.1	AJQI01	563	9.12	64.0	9076	8388
Bradyrhizobium sp. CCBAU 43298	GCA_000261685.1	AJQE01	508	7.92	63.9	7836	7123
Bradyrhizobium sp. CCGE-LA001	GCA_000296215.2	CP013949.1	-	7.83	63.6	7234	6988
Bradyrhizobium sp. CCH10-C7	GCA_001556255.1	LSIN01	366	5.59	60.7	5487	5116
Bradyrhizobium sp. CCH4-A6	GCA_001556245.1	LSIM01	249	5.30	60.9	5151	4852
Bradyrhizobium sp. CCH5-F6	GCA_001556045.1	LSIC01	222	8.16	64.1	7720	7383
Bradyrhizobium sp. CF659	GCA_900113725.1	FOQJ01	25	8.47	64.5	7968	7608
Bradyrhizobium sp. Cp5.3	GCA_000426265.1	AUFA01	148	8.92	63.4	8486	8000

Bradyrhizobium sp. DFCI-1	GCA_000465325.1	AMFB01	89	7.65	64.4	7164	7112
Bradyrhizobium sp. DOA1	GCA_001593325.1	JXJM01	4	7.54	63.6	7085	6648
Bradyrhizobium sp. DOA9	GCA_000617845.2	BAWE01	2	7.85	64.5	7364	7109
Bradyrhizobium sp. Ec3.3	GCA_000472925.1	AXAS01	154	10.0	62.6	9477	8845
Bradyrhizobium sp. ERR11	GCA_900094605.1	FMAI01	102	9.16	63.2	8688	8037
Bradyrhizobium sp. G22	GCA_900011265.1	CZWJ01	20	9.09	63.7	8606	8188
Bradyrhizobium sp. Gha	GCA_900113735.1	FOQM01	95	8.25	62.9	7934	7318
Bradyrhizobium sp. Ghvi	GCA_900115265.1	FOVU01	41	7.93	63.0	7536	7137
Bradyrhizobium sp. Leaf396	GCA_001424605.1	LMQJ01	66	7.71	63.8	7324	7020
Bradyrhizobium sp. LMTR 3	GCA_001693485.1	MAXC01	55	7.84	62.3	7328	7024
Bradyrhizobium sp. LTSP849	GCA_000938255.1	JYMR01	57	8.57	63.2	8063	7841
Bradyrhizobium sp. LTSP857	GCA_000938285.1	JYMS01	50	8.43	63.3	7947	7719
Bradyrhizobium sp. LTSP885	GCA_000938305.1	JYMT01	43	7.85	63.4	7295	7143
Bradyrhizobium sp. LTSPM299	GCA_000938235.1	JYMU01	100	9.09	62.8	8488	8171
Bradyrhizobium sp. OHSU III	GCA_000497675.1	APJD01	82	7.94	64.7	7417	7217
Bradyrhizobium sp. OK095	GCA_900109945.1	FOBX01	51	7.85	63.9	7417	7204
Bradyrhizobium sp. ORS 278	GCA_000026145.1	CU234118.1	-	7.46	65.5	6788	6589
Bradyrhizobium sp. ORS 285	GCA_900176205.1	LT859959.1	-	7.80	65.2	7015	6749
Bradyrhizobium sp. ORS 375	GCA_000239775.2	CAFI01	497	7.86	65.5	7230	6821
Bradyrhizobium sp. R5	GCA_900101405.1	FMZW01	124	9.45	63.6	8887	8309
Bradyrhizobium sp. Rc2d	GCA_900099855.1	FNCU01	117	9.58	62.8	9149	8477
Bradyrhizobium sp. Rc3b	GCA_900114915.1	FOUS01	49	8.38	63.7	7874	7487
Bradyrhizobium sp. S23321	GCA_000284275.1	AP012279.1	-	7.23	64.3	6849	6733
Bradyrhizobium sp. STM 3809	GCA_000239795.2	CAFJ01	803	7.31	66.2	6897	6413
Bradyrhizobium sp. STM 3843	GCA_000239815.2	CAFK01	350	8.44	63.6	7822	7470
Bradyrhizobium sp. th.b2	GCA_000426785.1	AUGA01	266	10.1	63.3	9677	8877
Bradyrhizobium sp. Tv2a-2	GCA_000472425.1	AXAI01	87	8.50	62.2	7955	7609
Bradyrhizobium sp. URHA0002	GCA_000426105.1	AUES01	144	6.97	62.2	6757	6390
Bradyrhizobium sp. URHA0013	GCA_000518345.1	JADK01	23	7.19	63.8	6856	6620
Bradyrhizobium sp. URHD0069	GCA_000701345.1	JNIJ01	129	6.79	60.7	6595	6156
Bradyrhizobium sp. USDA 3384	GCA_000472685.1	AXAD01	51	9.80	63.6	9242	8791
Bradyrhizobium sp. WSM1253	GCA_000244935.1	AHMB01	2	8.72	63.1	8346	7851
Bradyrhizobium sp. WSM1417	GCA_000515415.1	AZXU01	1	8.05	63.2	7628	7292
Bradyrhizobium sp. WSM1743	GCA_000473065.1	AXAZ01	163	8.34	63.4	7858	7222
Bradyrhizobium sp. WSM2254	GCA_000472645.1	AXAB01	100	9.09	63.9	8586	8130
Bradyrhizobium sp. WSM2793	GCA_000379585.1	ARBF01	84	8.87	63.5	8409	7865
Bradyrhizobium sp. WSM3983	GCA_000473045.1	AXAY01	70	8.79	63.0	8416	7942
Bradyrhizobium sp. WSM4349	GCA_000373125.1	ARDH01	104	8.29	63.1	8029	7404

Bradyrhizobium sp. WSM471	GCA_000244915.1	CM001442.1	-	7.78	63.4	7336	6996
Bradyrhizobium sp. YR681	GCA_000282615.1	AKIY01	351	7.83	64.7	7575	7139
Bradyrhizobium stylosanthis BR 446	GCA_001641335.1	LVEM01	22	8.80	64.6	8195	7957
Bradyrhizobium tropiciagri SEMIA 6148	GCA_001189845.1	LFLZ01	189	9.77	63.5	-	-
Bradyrhizobium valentinum LmjM3	GCA_001440405.1	LLXX01	242	8.85	61.9	8566	7796
Bradyrhizobium valentinum LmjM6	GCA_001440385.1	LLXY01	242	9.15	61.9	8718	8034
Bradyrhizobium viridifuturi SEMIA 690	GCA_001238275.1	LGTB01	152	8.81	64.0	8372	7130
Bradyrhizobium yuanmingense BR3267	GCA_001439885.1	LJYF01	55	7.90	63.9	7318	6847
Bradyrhizobium yuanmingense CCBAU 05623	GCA_000261785.1	AJQJ01	570	8.25	63.8	8077	7249
Bradyrhizobium yuanmingense CCBAU 10071	GCA_900094575.1	FMAE01	108	8.20	63.8	7826	7334
Bradyrhizobium yuanmingense CCBAU 25021	GCA_000261805.1	AJQK01	490	7.99	63.8	7810	7021
Bradyrhizobium yuanmingense CCBAU 35157	GCA_000261825.1	AJQL01	457	7.85	63.9	7612	6906
Bradyrhizobium yuanmingense CGMCC 1.3531[a]	2596583575	-	111	8.20	63.8	7860	7777
Brucella abortus 544[T]	GCA_000369945.1	AQIS01	3	3.30	57.2	3386	3155
Brucella abortus NCTC 10505	GCA_000740175.1	NZ_CP007700.1<br>NZ_CP007701.1	-	3.29	57.2	3371	3140
Brucella canis RM6/66[T]	GCA_000740335.1	NZ_CP007758.1<br>NZ_CP007759.1	-	3.31	57.2	3361	3152
Brucella melitensis 16M[T]	GCA_000007125.1	NC_003317.1<br>NC_003318.1	-	3.31	57.2	3369	3099
Brucella melitensis 20236	GCA_001431745.1	NZ_CP008750.1<br>NZ_CP008751.1	-	3.31	57.2	3381	3140
Brucella microti CCM 4915[T]	GCA_000022745.1	NC_013119.1<br>NC_013118.1	-	3.34	57.2	3366	3198
Brucella ovis ATCC 25840[T]	GCA_000016845.1	NC_009505.1<br>NC_009504.1	-	3.28	57.2	3395	3043
Chelatococcus daeguensis TAD1	GCA_001887265.1	NZ_CP018095.1	-	4.10	67.9	3848	3724
Chelatococcus sambhunathii DSM 18167[T]	GCA_001418005.1	LIOL01	35	4.37	67.8	4173	4018
Cohaesibacter marisflavi CGMCC 1.9157[T]	GCA_900115225.1	FOVR01	44	5.34	53.8	4868	4804
Cucumibacter marinus DSM 18995[T]	GCA_000429865.1	AUCR01	27	3.71	62.2	3586	3499
Devosia chinhatensis IPL18[T]	GCA_000969445.1	JZEY01	98	3.50	62.4	3398	3257
Devosia enhydra ATCC 23634[T]	GCA_900119845.1	FPKU01	11	4.22	65.6	4080	3975
Devosia lucknowensis L15[T]	GCA_900177655.1	FXWK01	3	3.72	62.8	3665	3584
Devosia soli GH2-10[T]	GCA_000970455.1	LAJG01	48	4.14	61.0	4062	3709
Ensifer sojae CCBAU 05684[T]	GCA_002288525.1	NZ_CP023067.1	-	6.09	62.0	5887	5474
Filomicrobium insigne CGMCC 1.6497[T]	GCA_900104305.1	FNJC01	11	3.85	57.4	3676	3562
Fulvimarina manganoxydans CGMCC 1.10972[T]	GCA_900176465.1	FWXR01	55	4.83	62.9	4528	4469
Fulvimarina pelagi DSM 15513[T]	GCA_001463845.1	BBWO01	61	3.77	61.3	3523	3419

Hoeflea olei JC234 T	GCA_001703635.1	LQZT01	74	4.72	65.5	4357	4181
Hoeflea phototrophica DFL-43 T	GCA_000154705.2	NZ_CM002917.1	-	4.47	59.8	4227	4118
Hyphomicrobium denitrificans ATCC 51888 T	GCA_000143145.1	NC_014313.1	-	3.64	60.8	3542	3473
Hyphomicrobium nitrativorans NL23 T	GCA_000503895.1	NC_022997.1	-	3.65	63.9	3400	3279
Hyphomicrobium zavarzinii ATCC 27496 T	GCA_000383415.1	ARTG01	20	4.65	63.7	4276	4175
Kaistia adipata DSM 17808 T	GCA_000423225.1	AUHO01	14	4.84	67.1	4545	4423
Kaistia soli DSM 19436 T	GCA_900129325.1	FQUP01	18	5.24	64.5	4892	4851
Liberibacter crescens BT-0	GCA_001543305.1	NZ_CP010522.1	-	1.52	35.4	1372	1299
Liberibacter crescens BT-1T	GCA_000325745.1	NC_019907.1	-	1.51	35.4	1363	1245
Maritalea myrionectae DSM 19524 T	GCA_000423365.1	AUHV01	12	3.54	52.6	3472	3416
Martelella endophytica YC6887 T	GCA_000960975.1	NZ_CP010803.1	-	4.82	62.1	4526	4393
Martelella mediterranea DSM 17316 T	GCA_000376125.1	AQWH01	75	5.64	62.4	5301	5068
Mesorhizobium australicum WSM2073 T	GCA_000230995.3	NC_019973.1	-	6.20	62.8	6068	5795
Mesorhizobium ciceri CC1192	GCA_001618825.1	NZ_CP015062.1	-	6.94	62.5	6826	6551
Mesorhizobium ciceri WSM1271	GCA_000185905.1	NC_014923.1	-	6.69	62.6	6532	6264
Mesorhizobium huakuii 7653R	GCA_000709395.1	NZ_CP006581.1	-	6.36	63.3	6288	5885
Mesorhizobium japonicum MAFF303099	GCA_000009625.1	NC_002678.2	-	7.04	62.5	7487	7114
Mesorhizobium opportunistum WSM2075 T	GCA_000176035.2	NC_015675.1	-	6.88	62.9	6779	6514
Mesorhizobium sophorae ICMP 19535 T	GCA_002270415.1	NNRI01	24	8.05	62.2	7780	7205
Mesorhizobium sp. WSM1497	GCA_001672455.2	NZ_CP021070.1	-	7.20	62.4	7008	6654
Methylobacterium extorquens DM4 T	GCA_000083545.1	NC_012988.1	-	6.12	68.0	5763	5503
Methylobacterium nodulans ORS 2060 T	GCA_000022085.1	NC_011894.1	-	8.84	68.5	7471	7020
Methylobacterium oryzae CBMB20 T	GCA_000757795.1	NZ_CP003811.1	-	6.29	69.8	5771	5530
Methylobacterium phyllosphaerae CBMB27 T	GCA_001936175.1	NZ_CP015367.1	-	6.32	69.6	5722	5506
Methylobacterium populi BJ001 T	GCA_000019945.1	NC_010725.1	-	5.85	69.4	5480	5289
Methylocapsa aurea KYG T	GCA_000746085.1	JQKO01	37	3.97	61.3	3703	3551
Methylocapsa palsarum NE2 T	GCA_900114285.1	FOSN01	78	4.11	61.7	3885	3820
Methylocella silvestris BL2 T	GCA_000021745.1	NC_011666.1	-	4.31	63.1	4011	3873
Methylocystis bryophila S285	GCA_002117405.1	NZ_CP019948.1	-	4.53	63.2	4286	4150
Methylocystis rosea SV97 T	GCA_000372845.1	ARCT01	2	3.91	62.5	3895	3709
Methyloferula stellata AR4 T	GCA_000385335.1	ARWA01	1	4.24	59.6	3961	3877
Methylosinus trichosporium OB3b T	GCA_002752655.1	CP023737.1	-	5.18	65.8	4806	4565
Microvirga lotononidis WSM3557 T	GCA_000262405.1	AJUA01	104	7.08	63.1	6915	6524
Microvirga massiliensis JC119 T	GCA_001006805.1	CDSD01	50	9.17	62.8	8575	8045
Neomegalonema perideroedes DSM 15528 T	GCA_000374145.1	ARFG01	22	3.41	67.2	3121	3023
Neorhizobium galegae HAMBI 540 T	GCA_000731315.1	NZ_HG938353.1	-	6.46	61.3	6271	6033
Nitratireductor basaltis RR3-28	GCA_001953055.1	NZ_CP019044.1	-	3.36	58.6	3237	3082
Nitratireductor soli ZZ-1 T	GCA_001050155.1	LFVY01	60	5.13	64.1	5000	4839

Nitrobacter hamburgensis X14 [T]	GCA_000013885.1	NC_007964.1	-	4.41	61.7	4240	396
Nitrobacter vulgaris Ab1	GCA_002028545.1	MWPQ01	96	3.90	59.8	3739	3501
Nitrobacter winogradskyi Nb-255 [T]	GCA_000012725.1	NC_007406.1	-	3.40	62.0	3224	2984
Ochrobactrum anthropi ATCC 49188 [T]	GCA_000017405.1	NC_009667.1<br>NC_009668.1	-	5.21	56.2	5087	4870
Ochrobactrum intermedium LMG 3301 [T]	GCA_000182645.1	ACQA01	4	4.73	57.7	4571	4269
Oligotropha carboxidovorans ATCC 49405 [T]	GCA_000021365.1	NC_011386.1	-	3.75	62.4	3616	3518
Paramesorhizobium deserti A-3-E [T]	GCA_001558695.1	LNTU011	41	5.48	60.9	5149	4946
Pararhizobium polonicum F5.1 [T]	GCA_001687365.1	LGLV01	31	5.90	61.2	5591	5430
Pelagibacterium halotolerans B2 [T]	GCA_000230555.1	NC_016078.1	-	3.94	61.4	3858	3755
Pelagibacterium luteolum CGMCC 1.10267 [T]	GCA_900100665.1	FNCS01	51	4.30	60.4	4327	4266
Pleomorphomonas koreensis DSM 23070 [T]	GCA_000425185.1	AULH01	76	4.62	67.2	4318	4230
Pleomorphomonas oryzae DSM 16300 [T]	GCA_000422965.1	AUHB01	39	5.23	63.0	4857	4733
Prosthecomicrobium hirschii 16	GCA_001305515.1	LJYW01	52	6.48	68.8	5710	5502
Pseudaminobacter manganicus JH-7 [T]	GCA_002075885.1	MEDT01	60	4.84	61.2	4685	4504
Pseudaminobacter salicylatoxidans KCT001	GCA_000304395.1	CAIU01	53	4.61	62.8	4456	3981
Pseudorhizobium pelagicum R1-200B4	GCA_000722615.1	JAKI01	62	5.14	62.8	4998	4791
Rhizobium etli CFN 42 [T]	GCA_000092045.1	NC_007761.1	-	6.53	61.1	6435	5992
Rhizobium favelukesii LPU83 [T]	GCA_000577275.1	NZ_HG916852.1	-	7.04	59.6	8202	7513
Rhizobium gallicum R602 [T]	GCA_000816845.1	NZ_CP006877.1	-	7.31	59.6	7138	6674
Rhizobium grahamii CCGE 502 [T]	GCA_000298315.2	AEYE02	80	7.15	59.4	6898	6844
Rhizobium leguminosarum WSM1689	GCA_000517605.1	NZ_CP007045.1	-	6.90	60.9	6760	6429
Rhizobium tropici CIAT 899 [T]	GCA_000330885.1	NC_020059.1	-	6.69	59.5	6419	6208
Rhodoblastus acidophilus DSM 137 [T]	GCA_900187365.1	FYDG01	74	4.71	65.3	4490	4422
Rhodomicrobium vannielii ATCC 17100 [T]	GCA_000166055.1	NC_014664.1	-	4.02	62.2	3724	3600
Rhodopseudomonas palustris BisA53	GCA_000014825.1	NC_008435.1	-	5.51	64.4	4959	4834
Rhodopseudomonas palustris BisB18	GCA_000013745.1	NC_007925.1	-	5.51	65.0	5000	4858
Rhodopseudomonas palustris CGA009	GCA_000195775.1	NC_005296.1	-	5.47	65.0	4996	4874
Rhodopseudomonas palustris HaA2	GCA_000013365.1	NC_007778.1	-	5.33	66.0	4785	4673
Rhodopseudomonas pseudopalustris DSM 123 [T]	GCA_900110435.1	FODT01	29	5.27	64.6	4883	4821
Salinarimonas rosea DSM 21201 [T]	GCA_000429045.1	AUBC01	62	5.25	71.8	4903	4735
Sinorhizobium fredii NGR234	GCA_000018545.1	NC_012587.1	-	6.89	62.4	6472	6362
Sinorhizobium fredii USDA 205T	GCA_001461695.1	AUTC01	209	6.58	62.3	6377	5774
Sinorhizobium meliloti 1021	GCA_000006965.1	NC_003047.1	-	6.69	62.2	6292	6218
Tardiphaga robiniae Vaf-07	GCA_001618955.1	LVYV01	56	6.36	61.5	5883	5722
Tardiphaga sp. OK246	GCA_900188215.1	FZOX01	19	6.75	61.4	6346	6165
Terasakiella pusilla DSM 6293 [T]	GCA_000688235.1	JHYO01	85	4.05	50.0	3967	3783

[a]Genomes downloaded from the JGI IMG server instead of the NCBI ftp server.
