# Supplementary material for: Phylogenomic Analyses of Bradyrhizobium Reveal Uneven Distribution of the Lateral and Subpolar Flagellar Systems, Which Extends to Rhizobiales
Source: Microorganisms. 2019 Feb 13;7(2):50. doi: 10.3390/microorganisms7020050 (PMC6406911; doi:10.3390/microorganisms7020050)
Supplement: Supplementary file 1 [file microorganisms-07-00050-s001.zip › Supplementary_Table_S2.pdf]

Supplementary Table S2. Subpolar and lateral flagellar systems distribution in sequenced *Bradyrhizobium* genomes used in this study.

Genome name	Subpolar flagellum	Lateral flagellum	Subpolar flagellum ascc. No.	Lateral flagellum ascc. No.
Bradyrhizobium liaoningense CCBAU 05525	+	+	AJQC01000206.1	AJQC01000679.1
Bradyrhizobium liaoningense CCBAU 83689	+	+	AJQD01000348.1	AJQD01000052.1
Bradyrhizobium japonicum USDA 135	+	-	AXAT01000174.1	-
Bradyrhizobium liaoningense CCNWSX0360	+	+	LUKO01000219.1	LUKO01000044.1
Bradyrhizobium sp. CCBAU 43298	+	+	AJQE01000185.1	AJQE01000346.1
Bradyrhizobium sp. CCH5-F6	+	+	LSIC01000115.1	LSIC01000022.1
Bradyrhizobium japonicum is5	+	+	ATWP01000009.1	ATWP01000005.1
Bradyrhizobium japonicum in8p8	+	+	AUGD01000009.1	AUGD01000005.1
Bradyrhizobium sp. Leaf396	+	+	LMQJ01000065.1	LMQJ01000012.1
Bradyrhizobium yuanmingense CGMCC 1.3531[b]	+	+	IQ15DRAFT_scaffold00012.12	IQ15DRAFT_scaffold00011.11
***Bradyrhizobium yuanmingense* CCBAU 10071[T]**	**+**	**+**	**FMAE01000011.1**	**FMAE01000001.1**
Bradyrhizobium yuanmingense CCBAU 25021	±	+	AJQK01000243.1	AJQK01000228.1
Bradyrhizobium yuanmingense CCBAU 35157	+	+	AJQL01000348.1	AJQL01000201.1
Bradyrhizobium yuanmingense BR3267	+	+	LJYF01000050.1	LJYF01000042.1
Bradyrhizobium yuanmingense CCBAU 05623	+	+	AJQJ01000382.1	AJQJ01000476.1
Bradyrhizobium sp. DOA9	+	+	DF820425.1	DF820425.1
Bradyrhizobium sp. WSM1743	+	+	AXAZ01000061.1	AXAZ01000012.1
Bradyrhizobium sp. DOA1	+	+	JXJM01000001.1	JXJM01000001.1
Bradyrhizobium sp. CCGE-LA001	+	+	CP013949.1	CP013949.1
***Bradyrhizobium daqingense* CGMCC 1.10947T[Tb]**	**+**	**+**	IQ17DRAFT_scaffold00002.2	IQ17DRAFT_scaffold00009.9
Bradyrhizobium sp. Rc2d	+	+	FNCU01000064.1	FNCU01000001.1
Bradyrhizobium sp. CCBAU 15615	+	+	AJQG01000101.1	AJQG01000149.1
Bradyrhizobium japonicum USDA 4	+	+	AXAF01000018.1	KI421457.1
Bradyrhizobium sp. CCBAU 15635	+	+	AJQH01000144.1	AJQH01000061.1
Bradyrhizobium sp. CCBAU 15544	+	+	AJQF01000166.1	AJQF01000373.1
Bradyrhizobium sp. Rc3b	+	+	FOUS01000025.1	FOUS01000003.1
Bradyrhizobium sp. WSM2793	+	+	KB902809.1	KB902780.1
Bradyrhizobium sp. ERR11	+	+	FMAI01000022.1	FMAI01000011.1
Bradyrhizobium sp. AT1	+	+	JXDL01000001.1	JXDL01000001.1
Bradyrhizobium japonicum 22	+	-	KI519399.1	-
Bradyrhizobium japonicum USDA 124	+	+	KB893818.1	KB893805.1
Bradyrhizobium huanghuaihaiense CGMCC 1.10948[b]	+	+	IQ16DRAFT_scaffold00014.14	IQ16DRAFT_scaffold00006.6
***Bradyrhizobium stylosanthis* BR 446[T]**	**+**	**+**	**LVEM01000001.1**	**LVEM01000003.1**
Bradyrhizobium sp. USDA 3384	+	+	AXAD01000004.1	AXAD01000005.1
***Bradyrhizobium arachidis* LMG 26795[T]**	**+**	**+**	**FPBQ01000003.1**	**FPBQ01000002.1**
Bradyrhizobium japonicum FN1	+	+	JGCL01000027.1	JGCL01000003.1
Bradyrhizobium japonicum E109	+	+	CP010313.1	CP010313.1
***Bradyrhizobium japonicum* USDA 6[T]**	**+**	**+**	**AP012206.1**	**AP012206.1**
Bradyrhizobium japonicum CCBAU 25435	+	+	AJQA01000196.1	AJQA01000242.1
Bradyrhizobium japonicum SEMIA 5079	+	+	CP007569.1	CP007569.1

Bradyrhizobium japonicum CCBAU 15618	+	+	AJPZ01000150.1	AJPZ01000249.1
Bradyrhizobium japonicum USDA 38	+	+	AXAG01000016.1	KI421458.1
Bradyrhizobium japonicum Is-34	+	+	JRPN01000005.1	JRPN01000001.1
Bradyrhizobium japonicum CCBAU 83623	+	+	AJQB01000715.1	AJQB01000642.1
Bradyrhizobium japonicum CCBAU 15354	+	+	AJPX01000310.1	AJPX01000508.1
Bradyrhizobium japonicum CCBAU 15517	+	+	AJPY01000263.1	AJPY01000027.1
Bradyrhizobium japonicum USDA 123	+	+	AXVP01000018.1	AXVP01000003.1
Bradyrhizobium sp. G22	+	+	LN907826.1	LN907826.1
Bradyrhizobium japonicum Is-1	+	+	LGUJ01000012.1	LGUJ01000001.1
Bradyrhizobium diazoefficiens USDA 122	+	+	CP013127.1	CP013127.1
Bradyrhizobium diazoefficiens SEMIA 5080	+	+	ADOU02000005.1	ADOU02000005.1
Bradyrhizobium sp. CCBAU 41267	+	+	AJQI01000329.1	AJQI01000093.1
***Bradyrhizobium diazoefficiens* USDA 110^T**	**+**	**+**	**BA000040.2**	**BA000040.2**
Bradyrhizobium diazoefficiens NK6	+	-	AP014685.1	-
Bradyrhizobium sp. CF659	+	+	FOQJ01000013.1	FOQJ01000002.1
Bradyrhizobium sp. WSM2254	+	+	KI421444.1	AXAB01000032.1
Bradyrhizobium sp. YR681	+	+	AKIY01000099.1	AKIY01000020.1
Bradyrhizobium sp. OK095	+	+	FOBX01000006.1	FOBX01000014.1
Bradyrhizobium sp. S23321	+	+	AP012279.1	AP012279.1
Bradyrhizobium sp. WSM471	+	+	CM001442.1	CM001442.1
Bradyrhizobium sp. BF49	+	+	LN901633.1	LN901633.1
Bradyrhizobium sp. WSM1253	+	+	JH600073.1	JH600073.1
Bradyrhizobium sp. WSM1417	+	+	KI911783.1	KI911783.1
Bradyrhizobium sp. URHA0013	+	+	JADK01000009.1	KI912427.1
Bradyrhizobium sp. WSM4349	+	+	KB890518.1	KB890499.1
Bradyrhizobium sp. LTSP849	+	+	JYMR01000051.1	JYMR01000052.1
Bradyrhizobium sp. LTSP857	+	+	JYMS01000049.1	JYMS01000047.1
Bradyrhizobium sp. WSM3983	+	+	AXAY01000034.1	KI421489.1
Bradyrhizobium sp. Gha	+	+	FOQM01000005.1	-
Bradyrhizobium sp. Ghvi	+	+	FOVU01000004.1	-
***Bradyrhizobium manausense* BR3351^T**	**+**	**+**	**LJYG01000054.1**	**LJYG01000026.1**
Bradyrhizobium sp. Cp5.3	+	+	AUFA01000010.1	KE384515.1
***Bradyrhizobium neotropicale* BR 10247^T**	**+**	**+**	**LSEF01000057.1**	**LSEF01000114.1**
Bradyrhizobium sp. BR 10245	+	+	LUUB01000034.1	LUUB01000096.1
Bradyrhizobium sp. Ec3.3	+	+	AXAS01000048.1	AXAS01000025.1
Bradyrhizobium elkanii CCBAU 43297	+	-	AJPW01000504.1	-
***Bradyrhizobium elkanii* USDA 76^T**	**+**	**-**	**KB900701.1**	-
Bradyrhizobium elkanii 587	+	-	AJJK01002424	-
Bradyrhizobium elkanii BLY6-1	+	-	LXEM01000041.1	-
Bradyrhizobium elkanii BLY3-8	+	-	LWUI01000007.1	-
Bradyrhizobium elkanii CCBAU 05737	+	-	AJPV01000282.1	-
Bradyrhizobium elkanii USDA 94	+	-	JAFC01000011.1	-
Bradyrhizobium elkanii USDA 3254	+	-	AXAH01000036.1	-
Bradyrhizobium elkanii USDA 3259	+	-	AXAW01000035.1	-
***Bradyrhizobium pachyrhizi* PAC48^T**	**+**	**-**	**LFIQ01000120.1**	-
Bradyrhizobium sp. R5	+	-	FMZW01000021.1	-

Bradyrhizobium pachyrhizi BR3262	+	-	LJYE01000083.1	-
Bradyrhizobium erythrophlei MT12	+	-	**FNTH01000001.1**	-
***Bradyrhizobium embrapense* SEMIA 6208^{T}**	**+**	**-**	**LFIP02000003.1**	-
***Bradyrhizobium tropiciagri* SEMIA 6148^{T}**	**+**	**-**	**LFIP02000003.1**	-
***Bradyrhizobium viridifuturi* SEMIA 690^{T}**	**+**	**-**	**LGTB01000070.1**	-
Bradyrhizobium sp. 17-4	+	-	BBEX01000323	-
Bradyrhizobium sp. DFCI-1	+	-	KE747856.1	-
Bradyrhizobium sp. th.b2	+	-	KE384569.1	-
Bradyrhizobium elkanii UASWS1015	+	-	JXOF01000179.1	-
Bradyrhizobium sp. OHSU III	+	-	APJD01000008.1	-
Bradyrhizobium sp. LTSP885	+	-	JYMT01000040.1	-
Bradyrhizobium sp. LTSPM299	+	-	JYMU01000006.1	-
Bradyrhizobium sp. BR 10303	+	-	LNCU01000116.1	-
Bradyrhizobium elkanii WSM2783	+	-	AXAP01000001.1	-
Bradyrhizobium sp. Ai1a-2	+	-	AUEZ01000036.1	-
Bradyrhizobium sp. STM 3809	+	-	CAFJ01000695.1	-
Bradyrhizobium sp. ORS 278	+	-	CU234118.1	-
Bradyrhizobium sp. ORS 375	+	-	CAFI01000219.1	-
Bradyrhizobium sp. ORS 285	+	-	LT859959.1	-
***Bradyrhizobium oligotrophicum* S58^{T}**	+	-	**NC_020453.1**	-
Bradyrhizobium sp. BTAi1	+	-	CP000494.1	-
Bradyrhizobium sp. STM 3843	+	-	CAFK01000334.1	-
Bradyrhizobium sp. Tv2a-2	+	-	AXAI01000011.1	-
Bradyrhizobium sp. ARR65	+	-	AWZU01000062.1	-
Bradyrhizobium sp. LMTR 3	+	-	MAXC01000053.1	-
***Bradyrhizobium retamae* Ro19^{T}**	**+**	**-**	**LLYA01000191.1**	-
Bradyrhizobium elkanii WSM1741	+	-	AXAU01000009.1	-
***Bradyrhizobium icense* LMTR 13^{T}**	**+**	**-**	**CP016428**	-
Bradyrhizobium valentinum LmjM6	+	-	LLXY01000001.1	-
***Bradyrhizobium valentinum* LmjM3^{T}**	**+**	**-**	**LLXX01000235.1**	-
***Bradyrhizobium paxllaeri* LMTR 21^{T}**	**+**	**-**	**MAXB01000079.1**	-
***Bradyrhizobium jicamae* PAC68^{T}**	+	-	LLXZ01000016.1	-
Bradyrhizobium lablabi GAS522	+	-	FNTI01000001.1	-
Bradyrhizobium ottawaense GAS524	+	-	LT629693.1	-
Bradyrhizobium sp. URHA0002	+	-	AUES01000035.1	-
Bradyrhizobium erythrophlei GAS138	+	-	LT670817.1	-
Bradyrhizobium sp. URHD0069	+	-	JNIJ01000018.1	-
Bradyrhizobium erythrophlei GAS242	+	-	LT670818.1	-
Bradyrhizobium canariense GAS369	+	-	LT629750.1	-
Bradyrhizobium erythrophlei GAS401	+	-	LT670849.1	-
Bradyrhizobium sp. CCH4-A6	+	-	LSIM01000119.1	-
Bradyrhizobium sp. CCH10-C7	+	-	LSIN01000025.1	-

[b]Genomes downloaded from the JGI IMG.
Type strains are indicated with ^{T} and bold.
