# Supplementary material for: Phylogenomic Analyses of Bradyrhizobium Reveal Uneven Distribution of the Lateral and Subpolar Flagellar Systems, Which Extends to Rhizobiales
Source: Microorganisms. 2019 Feb 13;7(2):50. doi: 10.3390/microorganisms7020050 (PMC6406911; doi:10.3390/microorganisms7020050)
Supplement: Supplementary file 1 [file microorganisms-07-00050-s001.zip › Supplementary_Table_S3.pdf]

Supplementary Table S3. First 10 closest relatives of 31 *Bradyrhizobium diazoefficiens* USDA 110^{T} protein sequences of the lateral flagellar system based on blastp against the nr NCBI protein database. *Rhodopseudomonas* and *Tardiphaga* are highlighted in green and orange respectively.

Query ID	Closest relative	Subject ID	Identity %	Align. length	Mismatches	Gap opens	Q. start	Q. end	S. start	S. end	evalue	bit score	Cover . %
LafR	*Rhodopseudomonas palustris* BisA53	WP_011662623.1	62.34	231	83	1	9	235	1	231	2.00E-97	298	86
	Tardiphaga sp. OK245	SEH75824.1	60.18	221	88	0	9	229	1	221	9.00E-94	288	84
	Tardiphaga sp. Vaf0	WP_068729849.1	60.18	221	88	0	9	229	1	221	1.00E-93	288	84
	Rhodopseudomonas palustris BisB18	WP_011471593.1	60.18	221	88	0	9	229	1	221	2.00E-91	282	84
	Microvirga massiliensis	WP_046868826.1	56.42	218	95	0	9	226	1	218	1.00E-80	254	83
	Pseudaminobacter salicylatoxidans	WP_019173192.1	54.09	220	101	0	9	228	1	220	9.00E-79	249	83
	Fulvimarina pelagi HTCC2506	WP_007067687.1	52.7	222	105	0	9	230	1	222	9.00E-79	249	84
	Aquamicrobium defluvii	WP_035024249.1	55.66	221	96	2	9	228	1	220	1.00E-78	249	83
	Mesorhizobium sp. WSM1293	WP_027161427.1	54.55	220	100	0	9	228	1	220	1.00E-78	249	83
	Beijerinckia indica ATCC 9039	WP_012386292.1	54.59	218	99	0	9	226	1	218	2.00E-78	249	83
FlgN	*Pleomorphomonas oryzae*	WP_051228408.1	37.91	153	81	4	12	161	1	142	7.00E-20	92.4	88
	Rhodopseudomonas palustris BisA53	WP_011662621.1	45.1	102	56	0	61	162	52	153	4.00E-19	90.5	60
	Tardiphaga sp. Vaf07	WP_068729853.1	43.14	102	58	0	61	162	54	155	1.00E-18	89.4	60
	Afifella marina DSM 2698	SCZ28776.1	43.81	105	59	0	58	162	14	118	2.00E-18	87.8	62
	Tardiphaga sp. OK245	SEH75855.1	42.16	102	59	0	61	162	54	155	3.00E-18	88.2	60
	Methylobacterium nodulans ORS 2060	WP_015933062.1	43.27	104	59	0	60	163	2	105	4.00E-18	86.7	61
	Hyphomicrobium sp. CS1GBMeth3	WP_072397425.1	46	100	54	0	63	162	2	101	6.00E-18	85.9	59
	Hyphomicrobium nitrativorans NL23	WP_023786207.1	34.84	155	86	3	11	162	1	143	1.00E-17	86.3	89
	Afifella pfennigii	WP_051630254.1	43.81	105	59	0	60	164	17	121	1.00E-17	85.5	62
	Aurantimonas sp. Leaf443	WP_055852416.1	38.83	103	63	0	60	162	5	107	2.00E-17	84.7	61
FlgJ	*Rhodopseudomonas palustris* BisB18	WP_011471590.1	52.32	151	63	4	12	156	1	148	4.00E-39	142	85
	Tardiphaga sp. OK245	SEH75870.1	50.35	143	65	3	19	160	9	146	2.00E-38	140	84
	Tardiphaga sp. Vaf07	WP_068729855.1	50.35	143	65	3	19	160	9	146	2.00E-38	140	84
	Rhodopseudomonas palustris BisA53	WP_011662620.1	52.63	152	60	5	12	155	1	148	3.00E-35	132	85
	Sinorhizobium americanum CCGM7	WP_037378661.1	49.67	151	67	4	12	161	1	143	4.00E-32	125	88
	Sinorhizobium saheli	WP_066875342.1	49.33	150	69	3	12	161	1	143	8.00E-32	124	88
	Sinorhizobium sp. CCBAU 05631	WP_037423965.1	49.67	151	67	4	12	161	1	143	1.00E-31	124	88
	Rhizobium sp. 2MFCol3.1	WP_026282998.1	47.22	144	70	2	12	155	1	138	1.00E-31	124	85
	Rhizobium grahamii CCGE 502	WP_016552788.1	47.22	144	70	3	12	155	1	138	2.00E-31	123	85
	Rhizobium favelukesii	CDM56506.1	44.67	150	77	2	6	155	28	171	2.00E-31	124	88

FliR	*Rhodopseudomonas palustris* BisA53	WP_011662618.1	59.04	249	100	2	1	249	1	247	2.00E-84	264	99
	Rhodopseudomonas palustris BisB18	WP_011471588.1	57.43	249	104	2	1	249	1	247	2.00E-80	254	99
	Tardiphaga sp. OK245	SEH75895.1	56.22	249	107	2	1	249	1	247	7.00E-80	253	99
	Tardiphaga sp. Vaf07	WP_068729859.1	55.82	249	108	2	1	249	1	247	1.00E-79	253	99
	Microvirga ossetica	ANY79453.1	50.4	250	124	0	1	250	1	250	4.00E-79	251	100
	Labrenzia alexandrii	WP_050776216.1	48.96	241	123	0	9	249	11	251	1.00E-73	237	96
	Methylobacterium sp. 174MFSha1.1	SFV13187.1	45.45	242	132	0	7	248	7	248	2.00E-72	234	97
	Methylobacterium sp. WSM2598	WP_018263278.1	50	244	122	0	7	250	12	255	5.00E-72	233	98
	Methylobacterium sp. 4-46	WP_012336149.1	50	244	122	0	7	250	12	255	6.00E-72	233	98
	Labrenzia sp. OB1	KZM48079.1	48.98	245	123	2	6	249	8	251	6.00E-72	233	98
FlhA	*Rhodopseudomonas palustris* BisB18	WP_011471587.1	76.69	695	160	1	1	693	1	695	0	1065	100
	Tardiphaga sp. OK245	SEH75910.1	76.55	695	161	1	1	693	1	695	0	1036	100
	Rhodopseudomonas palustris BisA53	WP_011662617.1	76.83	695	159	1	1	693	1	695	0	1036	100
	Tardiphaga sp. Vaf07	WP_068729860.1	76.26	695	163	1	1	693	1	695	0	1034	100
	Methylocella silvestris BL2	WP_012589102.1	70.4	696	203	2	1	693	1	696	0	996	100
	Methylocapsa palsarum	SFK04492.1	69.81	699	202	3	1	693	1	696	0	991	100
	Chelatococcus sambhunathii	WP_055457833.1	71.22	695	198	1	1	693	1	695	0	991	100
	Pseudoxanthobacter soli DSM 19599	WP_073626723.1	69.67	699	206	3	1	693	1	699	0	989	100
	Ochrobactrum rhizosphaerae	WP_024895880.1	69.54	696	201	3	6	693	4	696	0	989	99
	Ochrobactrum sp. CDB2	WP_007877012.1	69.7	693	205	2	6	693	4	696	0	988	99
FliQ	*Rhodopseudomonas palustris* BisB18	WP_011471586.1	76.14	88	21	0	1	88	1	88	1.00E-40	140	100
	Rhodopseudomonas palustris BisA53	WP_011662616.1	73.86	88	23	0	1	88	1	88	5.00E-40	139	100
	Rhizobium sp. Root482	WP_056329526.1	70.45	88	26	0	1	88	1	88	8.00E-37	131	100
	Azorhizobium doebereinerae	WP_029005600.1	70.45	88	26	0	1	88	1	88	1.00E-36	130	100
	Methylocapsa aurea	WP_036261320.1	71.59	88	25	0	1	88	1	88	1.00E-36	130	100
	Rhizobium arenae	WP_075292506.1	69.32	88	27	0	1	88	1	88	2.00E-36	130	100
	Rhizobium sp. Root1204	WP_060504651.1	69.32	88	27	0	1	88	1	88	2.00E-36	129	100
	Rhizobium giardinii	WP_018324196.1	69.32	88	27	0	1	88	1	88	3.00E-36	129	100
	Pararhizobium polonicum	WP_068957982.1	69.32	88	27	0	1	88	1	88	3.00E-36	129	100
	Sinorhizobium sp. A49	WP_077504318.1	70.45	88	26	0	1	88	1	88	6.00E-36	129	100
FlgD	*Tardiphaga* sp. OK245	SEH75945.1	63.48	115	42	0	24	138	22	136	3.00E-47	161	82
	Tardiphaga sp. Vaf07	WP_068729864.1	63.96	111	40	0	28	138	25	135	2.00E-46	159	79
	Rhodopseudomonas palustris BisA53	WP_011662615.1	65.18	112	39	0	27	138	24	135	8.00E-46	157	80
	Rhodopseudomonas palustris BisB18	WP_011471585.1	63.96	111	40	0	28	138	25	135	6.00E-44	153	79
	Mesorhizobium sp. YR577	SFU06062.1	59.65	114	43	1	27	140	24	134	1.00E-40	144	81
	Chelatococcus sambhunathii	WP_055457835.1	52.17	138	61	2	1	138	1	133	4.00E-39	140	99

	Hyphomicrobium sulfonivorans	WP_068462040.1	49.63	135	65	1	4	138	6	137	7.00E-39	140	96
	Hyphomicrobium sulfonivorans	KWT67430.1	49.63	135	65	1	4	138	11	142	8.00E-39	140	96
	Mesorhizobium sp. SCN 65-12	ODT17668.1	57.14	119	48	2	19	137	12	127	3.00E-38	138	85
	Mesorhizobium sp. 65-26	OJX71690.1	57.14	119	48	2	19	137	17	132	4.00E-38	137	85
FlbT	*Rhodopseudomonas palustris*	WP_041802495.1	62.2	127	48	0	1	127	1	127	6.00E-52	173	93
	Rhodopseudomonas palustris BisB18	ABD86679.1	62.2	127	48	0	1	127	5	131	8.00E-52	173	93
	Tardiphaga sp. OK245	SEH75959.1	62.12	132	50	0	1	132	5	136	2.00E-51	172	97
	Tardiphaga sp. Vaf07	WP_068730432.1	59.85	132	53	0	1	132	1	132	2.00E-50	169	97
	Rhodopseudomonas palustris BisA53	ABJ05128.1	58.91	129	53	0	1	129	5	133	2.00E-47	161	95
	Aurantimonas coralicida	WP_024350343.1	53.97	126	58	0	1	126	4	129	1.00E-43	152	93
	Aurantimonas manganoxydans	WP_009210322.1	53.97	126	58	0	1	126	4	129	3.00E-43	151	93
	Aurantimonas sp. USBA 369	WP_078707276.1	52.38	126	60	0	1	126	4	129	5.00E-43	151	93
	Methylocapsa aurea	WP_036261316.1	55.88	136	60	0	1	136	1	136	1.00E-42	149	100
	Beijerinckia mobilis	WP_034991350.1	52.21	136	65	0	1	136	1	136	4.00E-41	145	100
FlaF	*Rhodopseudomonas palustris* BisB18	WP_011471583.1	58.77	114	47	0	4	117	1	114	2.00E-40	142	97
	Rhodopseudomonas palustris BisA53	WP_011662613.1	52.63	114	54	0	4	117	1	114	2.00E-36	132	97
	Tardiphaga sp. OK245	SEH75973.1	55.26	114	51	0	4	117	1	114	3.00E-36	132	97
	Tardiphaga sp. Vaf07	WP_068729866.1	54.39	114	52	0	4	117	1	114	2.00E-35	129	97
	Pseudovibrio sp. W64	WP_063303770.1	46.36	110	59	0	8	117	5	114	1.00E-26	107	94
	Pseudovibrio sp. JE062	WP_008551915.1	47.27	110	58	0	8	117	5	114	2.00E-26	106	94
	Pseudovibrio stylochi	WP_068082031.1	48.65	111	55	2	8	117	5	114	1.00E-25	104	94
	Pseudovibrio sp. W74	WP_063288346.1	45.45	110	60	0	8	117	5	114	2.00E-25	103	94
	Pseudovibrio sp. POLY-S9	WP_057466032.1	46.36	110	59	0	8	117	5	114	3.00E-25	103	94
	Pseudovibrio sp. FO-BEG1	WP_014285252.1	46.36	110	59	0	8	117	5	114	4.00E-25	103	94
FlgL	*Tardiphaga* sp. Vaf07	WP_068729868.1	55.84	351	151	1	2	352	1	347	2.00E-128	385	99
	Rhodopseudomonas palustris BisB18	WP_011471582.1	57.26	351	146	1	2	352	1	347	7.00E-128	383	99
	Rhodopseudomonas palustris BisA53	WP_011662612.1	54.99	351	154	1	2	352	1	347	4.00E-124	374	99
	Tardiphaga sp. OK245	SEH75988.1	51.85	351	165	1	2	352	1	347	8.00E-111	340	99
	Salinarimonas rosea	WP_029030043.1	43.91	353	192	2	2	352	1	349	3.00E-92	292	99
	Cohaesibacter marisflavi	SFN83554.1	43.97	348	191	1	5	352	3	346	4.00E-89	284	99
	Beijerinckia mobilis	WP_034991347.1	44.44	351	191	1	2	352	1	347	3.00E-88	281	99
	Labrenzia sp. CP4	WP_062488688.1	44.44	351	192	2	2	352	1	348	6.00E-88	281	99
	Bosea vaviloviae	WP_069688505.1	45.99	337	180	1	16	352	15	349	1.00E-86	278	96
	Bosea sp. RAC05	WP_069053035.1	43.63	353	193	2	2	352	1	349	1.00E-85	275	99
FlgK	*Rhodopseudomonas palustris* BisA53	WP_011662611.1	67.89	489	157	0	1	489	1	489	0	668	99
	Tardiphaga sp. OK245	SEH76002.1	66.33	490	164	1	1	490	1	489	0	652	99

	Tardiphaga sp. Vaf07	WP_068729871.1	64.49	490	173	1	1	490	1	489	0	637	99
	Rhodopseudomonas palustris BisB18	WP_011471581.1	65.92	490	167	0	1	490	1	490	0	636	99
	Bosea vaviloviae	WP_069688504.1	52.03	492	232	3	1	489	1	491	1.00E-163	485	99
	Pleomorphomonas oryzae	WP_026790439.1	52.76	489	223	4	1	489	1	481	1.00E-161	480	99
	Hyphomicrobium sp. NDB2Meth4	WP_072370935.1	51.12	491	237	3	1	490	1	489	5.00E-160	476	99
	Polymorphum gilvum SL003B-26A1	WP_013654603.1	52.12	495	229	3	1	488	1	494	1.00E-158	473	99
	Microvirga ossetica	ANY79446.1	52.32	495	223	6	1	490	1	487	3.00E-158	471	99
	Pannonibacter phragmitetus	WP_019963533.1	50.71	495	236	4	1	489	1	493	1.00E-156	467	99
	Tardiphaga sp. Vaf07	WP_068729872.1	60.85	424	144	5	1	410	1	416	3.00E-166	486	100
	Tardiphaga sp. OK245	SEH76016.1	60.38	424	146	5	1	410	1	416	2.00E-165	484	100
	Rhodopseudomonas palustris BisA53	WP_011662610.1	62.62	420	138	6	1	410	1	411	5.00E-160	470	100
	Rhodopseudomonas palustris BisB18	WP_011471580.1	60.24	420	148	6	1	410	1	411	5.00E-147	436	100
FlgE	*Phyllobacterium* sp. OV277	SDP79438.1	52.07	434	178	9	1	410	1	428	3.00E-128	389	100
	Mesorhizobium sp. F7	WP_047565792.1	51.72	435	171	10	1	410	1	421	6.00E-128	388	100
	Bosea sp. Root381	WP_056798292.1	51.64	428	179	10	1	410	1	418	3.00E-124	379	100
	Chelatococcus sambhunathii	WP_055457839.1	51.8	417	179	8	1	410	1	402	4.00E-124	378	100
	Chelatococcus daeguensis	WP_063187178.1	51.8	417	179	8	1	410	1	402	4.00E-124	378	100
	Mesorhizobium opportunistum	WP_013893199.1	52.19	433	169	11	1	410	1	418	1.00E-123	377	100
	Rhodopseudomonas palustris BisB18	WP_011471578.1	44.37	142	77	2	232	371	146	287	1.00E-23	110	32
	Tardiphaga sp. OK245	SEH76046.1	37.74	212	120	6	234	435	198	407	1.00E-21	106	46
	Tardiphaga sp. Vaf07	WP_068729874.1	39.57	139	80	3	225	360	189	326	7.00E-21	103	31
	Rhodopseudomonas palustris BisA53	WP_011662608.1	37.44	219	120	7	228	435	113	325	5.00E-20	100	48
FliK	*Bosea vaviloviae*	WP_069688529.1	29.84	258	148	8	185	426	143	383	2.00E-16	91.3	56
	Labrenzia sp. C1B10	WP_022998508.1	34.45	209	123	6	228	431	213	412	2.00E-14	85.9	47
	Labrenzia aggregata	WP_075282140.1	33.97	209	124	6	228	431	213	412	1.00E-13	83.2	47
	Labrenzia sp. CP4	WP_062488686.1	34.45	209	121	7	228	431	213	410	3.00E-13	82	47
	Pleomorphomonas oryzae	WP_026790470.1	33.77	151	83	5	215	355	192	335	4.00E-13	81.3	32
	Bosea sp. PAMC 26642	WP_066617408.1	32.72	162	92	5	240	392	185	338	1.00E-12	79.7	35
	Tardiphaga sp. Vaf07	WP_068729875.1	49.01	355	177	2	24	374	29	383	1.00E-100	316	91
	Tardiphaga sp. OK245	SEH76064.1	48.73	355	178	2	24	374	29	383	5.00E-100	315	91
	Rhodopseudomonas palustris BisB18	WP_011471577.1	48.18	357	180	4	25	376	30	386	4.00E-98	310	91
MotC	*Rhodopseudomonas palustris* BisA53	WP_011662607.1	44.2	371	203	2	19	385	24	394	4.00E-95	302	95
	Chelatococcus sambhunathii	WP_055457858.1	47.29	332	171	3	24	351	29	360	2.00E-86	282	85
	Chelatococcus daeguensis	WP_063187161.1	47.29	332	171	3	24	351	29	360	2.00E-86	282	85
	Methylocapsa aurea	WP_051953209.1	41.37	336	193	2	7	338	18	353	2.00E-77	257	86
	Azorhizobium caulinodans ORS 571	WP_012169155.1	43.93	321	177	2	20	337	390	710	5.00E-75	261	82

	Methyloferula stellata	WP_020176989.1	38.73	346	207	3	25	365	50	395	9.00E-73	245	88
	Azorhizobium doebereinerae	WP_051357059.1	43.91	312	172	2	25	333	430	741	1.00E-71	252	80
MotB	*Rhodopseudomonas palustris* BisB18	WP_011471576.1	46.43	448	209	12	2	445	3	423	5.00E-106	333	99
	Tardiphaga sp. OK245	SEH76075.1	45.55	461	198	14	7	441	8	441	6.00E-106	334	98
	Tardiphaga sp. Vaf07	WP_068729876.1	44.54	458	207	11	7	441	8	441	5.00E-105	332	98
	Methylobacterium aquaticum	WP_060848203.1	42.92	445	238	6	1	441	1	433	1.00E-98	315	99
	Methylobacterium sp. ap11	SEP39975.1	43.15	445	241	6	1	441	1	437	3.00E-98	314	99
	Polymorphum gilvum SL003B-26A1	WP_013654608.1	43.65	449	214	10	7	442	9	431	4.00E-98	313	98
	Methylobacterium variabile	WP_048442237.1	42.83	453	222	12	6	441	7	439	2.00E-97	312	98
	Methylobacterium aquaticum	WP_048463748.1	42.76	449	239	9	1	441	1	439	2.00E-97	312	99
	Microvirga massiliensis	WP_046866642.1	42.99	442	221	9	6	442	7	422	2.00E-97	311	98
	Phyllobacterium sp. YR531	WP_008121293.1	40.62	453	204	10	3	441	6	407	3.00E-97	310	99
FliF	*Rhodopseudomonas palustris* BisB18	WP_011471574.1	65.99	544	182	1	13	553	1	544	0	719	98
	Tardiphaga sp. OK245	SEH76103.1	64.84	546	186	3	13	552	1	546	0	713	98
	Tardiphaga sp. Vaf07	WP_068729879.1	64.84	546	186	3	13	552	1	546	0	712	98
	Rhodopseudomonas palustris BisA53	WP_011662604.1	65.51	548	174	2	13	551	1	542	0	708	97
	Chelatococcus sp. CO-6	WP_019404749.1	57.46	543	229	1	13	553	1	543	0	616	98
	Chelatococcus daeguensis	WP_063187202.1	57.27	543	230	1	13	553	1	543	0	610	98
	Chelatococcus sambhunathii	WP_055457861.1	57.27	543	230	1	13	553	1	543	0	610	98
	Microvirga guangxiensis	SCY06191.1	56.15	545	231	2	16	552	4	548	0	602	97
	Methylobacterium brachiatum	SFJ50956.1	56.41	546	226	5	16	553	4	545	0	595	97
	Methylobacterium sp. Leaf108	WP_056283382.1	55.3	557	233	3	13	553	1	557	0	593	98
FliP	*Rhodopseudomonas palustris* BisA53	WP_011662602.1	78.99	238	50	0	8	245	6	243	5.00E-126	370	97
	Tardiphaga sp. Vaf07	WP_068729883.1	79.3	227	47	0	19	245	17	243	1.00E-123	364	92
	Tardiphaga sp. OK245	SEH76131.1	79.3	227	47	0	19	245	18	244	8.00E-123	362	92
	Rhodopseudomonas palustris BisB18	WP_011471572.1	79.46	224	46	0	22	245	24	247	6.00E-122	360	91
	Methylobacterium platani JCM 14648	WP_048437206.1	77.13	223	51	0	23	245	34	256	5.00E-120	355	91
	Methylobacterium extorquens DM4	WP_015821058.1	77.13	223	51	0	23	245	37	259	5.00E-120	355	91
	Methylobacterium sp. 174MFSha1.1	SFV15311.1	77.13	223	51	0	23	245	38	260	6.00E-120	355	91
	Methylobacterium sp. Leaf123	WP_056194052.1	76.68	223	52	0	23	245	37	259	2.00E-119	354	91
	Methylobacterium extorquens CM4	WP_012605699.1	76.68	223	52	0	23	245	37	259	3.00E-119	353	91
	Methylobacterium extorquens PA1	WP_012252383.1	76.68	223	52	0	23	245	37	259	3.00E-119	353	91
FliL	*Rhodopseudomonas palustris*	WP_041800380.1	57.82	147	62	0	2	148	1	147	4.00E-54	179	99
	Rhodopseudomonas palustris BisA53	ABJ05115.1	57.14	147	63	0	2	148	17	163	2.00E-53	178	99
	Rhodopseudomonas palustris BisB18	WP_011471571.1	53.74	147	68	0	2	148	1	147	3.00E-51	172	99
	Tardiphaga sp. Vaf07	WP_068730434.1	53.06	147	69	0	2	148	1	147	2.00E-50	170	99

	Tardiphaga sp. OK245	SEH76144.1	52.03	148	71	0	1	148	14	161	3.00E-49	167	100
	Chelatococcus sambhunathii	WP_055457849.1	44.3	149	78	2	3	148	13	159	8.00E-39	140	99
	Chelatococcus daeguensis	WP_063187171.1	43.62	149	79	2	3	148	13	159	1.00E-38	140	99
	Polymorphum gilvum	WP_041376329.1	54.55	143	57	2	11	148	5	144	1.00E-36	134	93
	Polymorphum gilvum SL003B-26A1	ADZ72304.1	52	150	64	2	4	148	23	169	2.00E-36	135	98
	Chelatococcus sp. CO-6	WP_019402933.1	42.76	145	78	2	7	148	17	159	8.00E-36	133	96
FlgH	*Rhodopseudomonas palustris* BisA53	ABJ05114.1	55.95	227	100	0	5	231	8	234	1.00E-86	269	98
	Tardiphaga sp. Vaf07	WP_068730435.1	53.25	231	108	0	1	231	1	231	3.00E-82	258	100
	Tardiphaga sp. OK245	SEH76157.1	52.81	231	109	0	1	231	4	234	1.00E-81	256	100
	Tardiphaga sp. Vaf07	AMH39574.1	53.74	227	105	0	5	231	8	234	2.00E-81	256	98
	Bosea sp. PAMC 26642	WP_066617509.1	53.85	234	105	2	1	231	1	234	8.00E-78	246	100
	Microvirga lotononidis	WP_009493113.1	51.29	232	112	1	1	231	1	232	2.00E-76	243	100
	Methylobacterium brachiatum	SFJ51057.1	52.59	232	109	1	1	231	1	232	8.00E-75	239	100
	Microvirga lupini	WP_051454790.1	51.29	232	112	1	1	231	1	232	1.00E-74	238	100
	Bosea vaviloviae	WP_069688494.1|	53.85	234	105	2	1	231	1	234	2.00E-74	238	100
	Bosea sp. Root381	WP_056798268.1	51.71	234	110	2	1	231	1	234	5.00E-74	236	100
FlgI	*Tardiphaga* sp. Vaf07	WP_068730436.1	72.85	361	87	1	16	376	16	365	0	530	96
	Tardiphaga sp. OK245	SEH76185.1	72.93	362	86	2	16	376	17	367	0	530	96
	Rhodopseudomonas palustris BisA53	ABJ05112.1	70.29	377	100	2	1	376	2	367	1.00E-179	516	100
	Rhodopseudomonas palustris BisB18	ABD86663.1	71.35	377	96	2	1	376	2	367	8.00E-178	512	100
	Methylobacterium sp. Leaf117	WP_056484894.1	65.42	373	118	2	4	376	7	368	4.00E-161	469	99
	Methylobacterium sp. Leaf113	WP_056181387.1	65.15	373	119	2	4	376	7	368	1.00E-160	469	99
	Rhizobiales bacterium GAS113	SDR61185.1	65.55	357	122	1	21	376	75	431	5.00E-160	469	95
	Rhizobiales bacterium GAS188	SED39458.1	65.55	357	122	1	21	376	75	431	7.00E-160	469	95
	Methyloferula stellata	WP_020176999.1	62.5	376	130	1	1	376	1	365	7.00E-160	466	100
	Microvirga flocculans	WP_027316337.1	62.23	376	131	1	1	376	1	365	3.00E-159	464	100
FlgA	*Rhodopseudomonas palustris* BisA53	ABJ05111.1	57.53	146	61	1	30	174	16	161	5.00E-46	160	83
	Rhodopseudomonas palustris	WP_041800376.1	59.85	132	53	0	43	174	12	143	6.00E-46	159	75
	Tardiphaga sp. Vaf07	WP_068729887.1	52.9	155	71	1	22	174	4	158	3.00E-44	155	87
	Tardiphaga sp. OK245	SEH76198.1	55.64	133	59	0	42	174	26	158	3.00E-42	150	76
	Microvirga lotononidis	WP_009493116.1	45.95	148	80	0	24	171	4	151	6.00E-35	132	85
	Methylosinus sp. 3S-1	WP_003613354.1	48.99	149	73	2	27	174	3	149	8.00E-35	131	85
	Microvirga lupini	WP_036353842.1	45.77	142	77	0	30	171	10	151	2.00E-34	130	81
	Microvirga sp. BSC39	WP_036358817.1	46.15	143	77	0	29	171	9	151	5.00E-33	126	82
	Microvirga guangxiensis	SCY06422.1	46.9	145	75	1	29	171	6	150	2.00E-32	125	82
	Microvirga flocculans	WP_027316336.1	44.76	143	79	0	29	171	9	151	1.00E-31	123	82

FlgG	Rhodopseudomonas palustris BisA53	WP_011662596.1	78.24	262	57	0	1	262	1	262	5.00E-152	437	100
	Rhodopseudomonas palustris BisB18	WP_011471566.1	78.63	262	56	0	1	262	1	262	2.00E-151	436	100
	Tardiphaga sp. OK245	SEH76213.1	74.43	262	67	0	1	262	1	262	4.00E-143	415	100
	Tardiphaga sp. Vaf07	WP_068729889.1	74.05	262	68	0	1	262	1	262	2.00E-142	413	100
	Chelatococcus sambhunathii	WP_055457853.1	72.9	262	71	0	1	262	1	262	1.00E-136	399	100
	Chelatococcus sp. CO-6	WP_019402938.1	73.28	262	70	0	1	262	1	262	2.00E-136	398	100
	Salinarimonas rosea	WP_029030032.1	72.14	262	73	0	1	262	1	262	1.00E-135	396	100
	Labrenzia alexandrii	WP_055673361.1	66.79	262	87	0	1	262	1	262	9.00E-132	386	100
	Labrenzia alexandrii DFL-11	WP_008197053.1	66.41	262	88	0	1	262	1	262	2.00E-131	385	100
	Microvirga lotononidis	WP_009493117.1	69.08	262	81	0	1	262	1	262	6.00E-131	384	100
FliE	*Rhodopseudomonas palustris* BisB18	WP_011471565.1	64.55	110	34	3	1	109	1	106	7.00E-31	117	100
	Rhodopseudomonas palustris BisA53	WP_011662595.1	59.63	109	41	1	1	109	1	106	7.00E-30	114	100
	Tardiphaga sp. Vaf07	WP_068729891.1	58.18	110	41	2	1	109	1	106	2.00E-29	113	100
	Microvirga ossetica	ANY79425.1	69.57	69	21	0	41	109	41	109	7.00E-23	97.1	63
	Microvirga sp. BSC39	WP_036358811.1	60.71	84	32	1	26	109	28	110	1.00E-22	96.3	77
	Methylobacterium brachiatum	SFJ51193.1	59.09	88	32	1	22	109	27	110	1.00E-22	96.3	81
	Azorhizobium doebereinerae	WP_029005584.1	50.43	117	42	3	1	109	1	109	7.00E-22	94.4	100
	Rhizobiales bacterium GAS188	SED39315.1	48.62	109	54	1	1	109	1	107	8.00E-22	94	100
	Microvirga lupini	WP_036353848.1	65.22	69	24	0	41	109	41	109	2.00E-21	92.8	63
	Rhizobium arenae	WP_075292484.1	47.75	111	55	2	1	109	1	110	2.00E-21	92.8	100
FlgC	*Rhodopseudomonas palustris* BisB18	WP_011471564.1	73.72	137	36	0	1	137	1	137	5.00E-71	222	99
	Tardiphaga sp. OK245	SEH76238.1	73.19	138	37	0	1	138	1	138	1.00E-70	221	100
	Tardiphaga sp. Vaf07	WP_068730437.1	72.46	138	38	0	1	138	1	138	2.00E-70	220	100
	Bosea sp. Root381	WP_056798285.1	70.29	138	41	0	1	138	1	138	4.00E-67	212	100
	Bosea vaviloviae	WP_069688500.1	70.29	138	41	0	1	138	1	138	8.00E-67	211	100
	Bosea sp. PAMC 26642	WP_066617484.1	69.57	138	42	0	1	138	1	138	7.00E-65	206	100
	Hyphomicrobium nitrativorans NL23	WP_023786219.1	71.01	138	40	0	1	138	1	138	9.00E-65	206	100
	Methylocella silvestris BL2	WP_012589078.1	70.29	138	41	0	1	138	1	138	1.00E-64	206	100
	Bosea sp. RAC05	WP_069053030.1	66.67	138	46	0	1	138	1	138	1.00E-64	205	100
	Aurantimonas sp. USBA 369	WP_078707251.1	68.12	138	44	0	1	138	1	138	4.00E-64	204	100
FlgB	*Rhodopseudomonas palustris* BisB18	WP_011471563.1	61.6	125	47	1	8	132	1	124	2.00E-51	171	94
	Rhodopseudomonas palustris BisA53	ABJ05107.1	59.06	127	51	1	8	133	8	134	5.00E-50	168	95
	Rhodopseudomonas palustris	WP_041800375.1	58.27	127	52	1	8	133	1	127	2.00E-49	166	95
	Tardiphaga sp. Vaf07	WP_068729893.1	56.35	126	54	1	8	133	1	125	2.00E-48	164	95
	Microvirga lotononidis	EIM24941.1	54.33	127	58	0	7	133	24	150	1.00E-44	155	95
	Microvirga lotononidis	WP_040639265.1	53.54	127	59	0	7	133	1	127	1.00E-44	154	95

	Microvirga flocculans	WP_051435267.1	55.12	127	57	0	7	133	12	138	3.00E-44	153	95
	Microvirga ossetica	ANY79423.1	55.12	127	57	0	7	133	12	138	1.00E-43	152	95
	Microvirga guangxiensis	SCY06584.1	54.84	124	56	0	10	133	4	127	2.00E-43	151	93
	Chelatococcus sambhunathii	WP_055457856.1	53.66	123	57	0	10	132	3	125	5.00E-43	150	92
FlhB	*Tardiphaga* sp. Vaf07	WP_068729895.1	62.61	353	132	0	1	353	1	353	2.00E-161	469	98
	Tardiphaga sp. OK245	SEH76264.1	62.32	353	133	0	1	353	1	353	5.00E-161	468	98
	Rhodopseudomonas palustris BisA53	WP_011662592.1	61.76	353	135	0	1	353	1	353	7.00E-161	468	98
	Rhodopseudomonas palustris BisB18	WP_011471562.1	62.04	353	134	0	1	353	1	353	3.00E-159	464	98
	Methylobacterium sp. WSM2598	WP_018260418.1	60.86	350	135	2	1	349	1	349	1.00E-146	431	97
	Pannonibacter phragmitetus	WP_019963519.1	55.34	356	159	0	1	356	1	356	6.00E-146	430	99
	Methylobacterium sp. 4-46	WP_012333023.1	60.57	350	136	2	1	349	1	349	6.00E-146	430	97
	Labrenzia sp. OB1	WP_068418034.1	56.21	354	155	0	1	354	1	354	7.00E-146	430	98
	Pannonibacter phragmitetus	WP_050474412.1	54.78	356	161	0	1	356	1	356	5.00E-145	427	99
	Pannonibacter indicus	WP_055457260.1	55.06	356	160	0	1	356	1	356	1.00E-144	427	99
FliG	*Tardiphaga* sp. Vaf07	WP_068729897.1	64.71	340	118	1	8	345	5	344	1.00E-161	469	98
	Tardiphaga sp. OK245	SEH76281.1	64.71	340	118	1	8	345	5	344	2.00E-161	468	98
	Rhodopseudomonas palustris BisA53	WP_011662591.1	63.72	339	122	1	8	345	5	343	8.00E-161	466	98
	Rhodopseudomonas palustris BisB18	WP_011471561.1	64.94	328	115	0	18	345	16	343	2.00E-156	455	95
	Chelatococcus sambhunathii	WP_055457845.1	63.31	338	123	1	9	346	13	349	2.00E-153	448	98
	Chelatococcus sp. CO-6	WP_019402928.1	63.02	338	124	1	9	346	14	350	9.00E-153	446	98
	Afifella pfennigii	WP_051630248.1	60.67	328	129	0	15	342	12	339	2.00E-150	440	95
	Microvirga sp. BSC39	WP_036358842.1	58.06	341	143	0	5	345	2	342	3.00E-146	429	99
	Microvirga lupini	WP_036353817.1	58.06	341	143	0	5	345	2	342	4.00E-146	429	99
	Pseudoxanthobacter soli DSM 19599	WP_073626753.1	58.95	324	133	0	18	341	15	338	6.00E-146	429	94
FliN	*Tardiphaga* sp. OK245	SEH76298.1	66.94	124	34	1	1	117	1	124	1.00E-47	161	94
	Rhodopseudomonas palustris BisA53	WP_011662590.1	64.52	124	37	1	1	117	1	124	2.00E-46	158	94
	Tardiphaga sp. Vaf07	WP_068729899.1	66.13	124	33	2	1	117	1	122	3.00E-46	158	94
	Methylobacterium brachiatum	SFJ51806.1	69.91	113	34	0	6	118	13	125	2.00E-45	155	91
	Methylobacterium phyllosphaerae	WP_043348558.1	66.39	119	38	1	2	118	5	123	9.00E-44	151	94
	Microvirga flocculans	WP_027316349.1	79.79	94	19	0	25	118	11	104	1.00E-43	150	76
	Rhodopseudomonas palustris BisB18	WP_011471560.1	63.71	124	38	2	1	117	1	124	1.00E-43	151	94
	Microvirga lotononidis	EIM24921.1	66.1	118	35	1	1	118	1	113	1.00E-43	151	95
	Methylobacterium oryzae CBMB20	AIQ89866.1	67.24	116	36	1	5	118	1	116	2.00E-43	150	92
	Microvirga ossetica	ANY79440.1	67.26	113	36	1	7	118	5	117	3.00E-43	150	90
FliM	*Rhodopseudomonas palustris* BisB18	WP_011471558.1	56.27	311	136	0	3	313	1	311	9.00E-133	393	99
	Tardiphaga sp. OK245	SEH76328.1	56.27	311	136	0	3	313	1	311	5.00E-130	385	99

	Tardiphaga sp. Vaf07	WP_068729903.1	56.27	311	136	0	3	313	1	311	7.00E-130	385	99
	Rhodopseudomonas palustris	WP_041799496.1	55.95	311	137	0	3	313	1	311	1.00E-129	385	99
	Microvirga vignae	WP_047187614.1	53.69	298	138	0	16	313	12	309	9.00E-123	367	95
	Salinarimonas rosea	WP_029031688.1	55.85	299	132	0	14	312	15	313	1.00E-121	364	96
	Microvirga guangxiensis	SCY05701.1	52.68	298	141	0	16	313	12	309	4.00E-120	360	95
	Microvirga ossetica	ANY79442.1	52.01	298	143	0	16	313	12	309	4.00E-119	358	95
	Chelatococcus sp. CO-6	WP_019402925.1	54.73	296	134	0	18	313	17	312	6.00E-119	357	95
	Chelatococcus daeguensis	WP_055457947.1	54.39	296	135	0	18	313	13	308	2.00E-118	356	95
	Tardiphaga sp. Vaf07	AMH39587.1	74.14	290	75	0	57	346	6	295	1.00E-146	429	83
	Tardiphaga sp. OK245	SEH76342.1	74.14	290	75	0	57	346	6	295	1.00E-146	429	83
	Phyllobacterium sp. OV277	SDP79884.1	72.04	279	78	0	55	333	4	282	6.00E-144	422	80
	Ochrobactrum intermedium LMG 3301	EEQ94082.1	67.67	300	96	1	34	333	22	320	3.00E-143	421	86
MotA	*Ochrobactrum anthropi*	WP_061347648.1	70.14	278	83	0	56	333	5	282	2.00E-141	416	80
	Ochrobactrum anthropi ATCC 49188	WP_010660722.1	70.14	278	83	0	56	333	5	282	2.00E-141	415	80
	Ochrobactrum intermedium M86	WP_006471432.1	70.14	278	83	0	56	333	5	282	3.00E-141	415	80
	Ochrobactrum sp. EGD-AQ16	WP_021585290.1	70.14	278	83	0	56	333	5	282	4.00E-141	414	80
	Paramesorhizobium deserti	WP_068882774.1	70.76	277	81	0	56	332	5	281	6.00E-141	414	79
	Ochrobactrum sp. P6BS-III	WP_078340856.1	70.5	278	82	0	56	333	5	282	8.00E-141	414	80
	Rhodopseudomonas palustris BisA53	WP_011662584.1	70.82	377	110	0	1	377	63	439	0	560	99
	Tardiphaga sp. OK245	SEH76381.1	70.03	377	113	0	1	377	63	439	0	559	99
	Tardiphaga sp. Vaf07	WP_068729909.1	70.03	377	113	0	1	377	63	439	0	559	99
	Rhodopseudomonas palustris BisB18	WP_011471555.1	70.56	377	111	0	1	377	63	439	0	557	99
FliI	*Chelatococcus* sp. CO-6	WP_019402921.1	71.35	377	108	0	1	377	63	439	0	548	99
	Chelatococcus sambhunathii	WP_055457840.1	71.09	377	109	0	1	377	63	439	0	548	99
	Methyloferula stellata	WP_020177015.1	69.76	377	114	0	1	377	63	439	0	543	99
	Pleomorphomonas oryzae	WP_026790449.1	69.5	377	115	0	1	377	66	442	0	543	99
	Hyphomicrobium nitrativorans	WP_051388668.1	71.51	372	106	0	1	372	57	428	0	541	98
	Hyphomicrobium nitrativorans NL23	AHB47756.1	71.51	372	106	0	1	372	82	453	0	541	98
