# Supplementary material for: Phylogenomic Analyses of Bradyrhizobium Reveal Uneven Distribution of the Lateral and Subpolar Flagellar Systems, Which Extends to Rhizobiales
Source: Microorganisms. 2019 Feb 13;7(2):50. doi: 10.3390/microorganisms7020050 (PMC6406911; doi:10.3390/microorganisms7020050)
Supplement: Supplementary file 1 [file microorganisms-07-00050-s001.zip › Supplementary_Table_S4.pdf]

Supplementary Table S4. Blastx of 13 lateral flagellum genes of 10 representative Rhizobiales genomes against the nr NCBI protein database excluding *Rhizobiales* order genomes. Only first 3 genera hits are shown. Best blast hit of each gene is indicated in green.

Gene	Query	Subject species	% identity	% cover.	e-value	Subject Accs. No.
		Labrenzia sp. VG12	50%	86%	4.00E-68	WP_094070877.1
	Bradyrhizobium diazoefficiens USDA 110^T	*Pannonibacter phragmitetus*	44%	96%	6.00E-62	WP_050474407.1
		Polymorphum gilvum	47%	96%	7.00E-56	WP_013654593.1
		Labrenzia alexandrii	50%	97%	6.00E-65	WP_008194671.1
	Rhodopseudomonas palustris BisA53	*Pannonibacter phragmitetus*	47%	95%	3.00E-61	WP_050474407.1
		Polymorphum gilvum	48%	96%	1.00E-52	WP_013654593.1
		Pannonibacter phragmitetus	49%	96%	2.00E-60	WP_050474407.1
	Pleomorphomonas oryzae DSM 16300^T	*Labrenzia alexandrii*	47%	96%	2.00E-56	WP_008194671.1
		Pseudomonas bauzanensis	45%	96%	2.00E-51	EZQ16437.1
		Labrenzia sp. OB1	51%	93%	2.00E-71	KZM48079.1
	Cohaesibacter marisflavi CGMCC 1.9157^T	*Pannonibacter phragmitetus*	46%	94%	3.00E-66	WP_019963526.1
		Polymorphum gilvum	44%	94%	1.00E-54	WP_013654593.1
fliR		*Labrenzia alexandrii*	49%	97%	2.00E-81	WP_008194671.1
	Methylobacterium extorquens DM4^T	*Pannonibacter phragmitetus*	47%	97%	5.00E-76	WP_019963526.1
		Polymorphum gilvum	48%	96%	1.00E-66	WP_013654593.1
		Labrenzia marina	51%	98%	1.00E-71	WP_103223518.1
	Microvirga massiliensis JC119^T	*Pannonibacter indicus*	46%	97%	4.00E-64	WP_055457255.1
		Polymorphum gilvum	47%	97%	3.00E-62	WP_013654593.1
		Pannonibacter phragmitetus	51%	97%	9.00E-73	WP_019963526.1
	Bosea vaviloviae Vaf-18^T	*Labrenzia* sp. OB1	51%	96%	3.00E-72	KZM48079.1
		Polymorphum gilvum	52%	97%	1.00E-69	WP_013654593.1
		Labrenzia aggregata	53%	97%	1.00E-76	WP_006934609.1
	Methyloferula stellata AR4^T	*Pannonibacter phragmitetus*	48%	99%	3.00E-72	WP_050474407.1
		Pseudomonas bauzanensis	49%	96%	2.00E-61	EZQ16437.1
	Methylocella sylvestris BL2^T	*Labrenzia* sp. OB1	46%	98%	1.00E-54	KZM48079.1

		Pseudomonas bauzanensis	46%	96%	5.00E-51	EZQ16437.1
		Pannonibacter phragmitetus	42%	98%	6.00E-51	WP_050474407.1
		Pseudomonas bauzanensis	41%	97%	2.00E-60	EZQ16437.1
	Rhizobium etli CFN42T	*Ahrensia kielensis*	40%	98%	2.00E-55	WP_018690483.1
		Pannonibacter phragmitetus	37%	94%	2.00E-47	WP_050474407.1
flhA	*Bradyrhizobium diazoefficiens* USDA 110T	*Pannonibacter phragmitetus*	69%	91%	0	WP_019963527.1
		Labrenzia sp. VG12	67%	99%	0	WP_094070878.1
		Polymorphum gilvum	68%	91%	0	WP_013654594.1
	Rhodopseudomonas palustris BisA53	*Labrenzia aggregata*	68%	98%	0	WP_040439904.1
		Stappia aggregata IAM 12614	68%	98%	0	EAV44093.1
		Pannonibacter phragmitetus	68%	98%	0	WP_019963527.1
	Pleomorphomonas oryzae DSM 16300T	*Labrenzia aggregata*	72%	99%	0	WP_040439904.1
		Stappia aggregata IAM 12614	72%	99%	0	EAV44093.1
		Pannonibacter phragmitetus	72%	98%	0	WP_019963527.1
	Cohaesibacter marisflavi CGMCC 1.9157T	*Pannonibacter phragmitetus*	73%	99%	0	WP_019963527.1
		Labrenzia marina	73%	99%	0	WP_103223519.1
		Polymorphum gilvum	74%	99%	0	WP_013654594.1
	Methylobacterium extorquens DM4T	*Pannonibacter phragmitetus*	73%	98%	0	WP_019963527.1
		Polymorphum gilvum	72%	98%	0	WP_013654594.1
		Labrenzia alexandrii	71%	98%	0	WP_008192641.1
	Microvirga massiliensis JC119T	*Labrenzia* sp. VG12	73%	98%	0	WP_094070878.1
		Polymorphum gilvum	72%	98%	0	WP_013654594.1
		Pannonibacter phragmitetus	72%	98%	0	WP_019963527.1
	Bosea vaviloviae Vaf-18T	*Polymorphum gilvum*	72%	98%	0	WP_013654594.1
		Labrenzia sp. VG12	70%	99%	0	WP_094070878.1
		Pannonibacter phragmitetus	71%	98%	0	WP_019963527.1
	Methyloferula stellata AR4T	*Pannonibacter phragmitetus*	72%	99%	0	WP_050474406.1
		Labrenzia alexandrii	70%	99%	0	WP_008192641.1
		Polymorphum gilvum	72%	99%	0	WP_013654594.1

	Methylocella sylvestris BL2^T	*Pannonibacter phragmitetus*	71%	99%	0	WP_050474406.1
		Labrenzia sp. VG12	69%	99%	0	WP_094070878.1
		Stappia aggregata IAM 12614	70%	99%	0	EAV44093.1
	Rhizobium etli CFN42^T	*Pseudomonas bauzanensis*	73%	93%	0	EZQ16438.1
		Polymorphum gilvum	69%	93%	0	WP_013654594.1
		Labrenzia marina	67%	93%	0	WP_103223519.1
fliQ	*Bradyrhizobium diazoefficiens* USDA 110^T	*Pseudovibrio stylochi*	67%	98%	3.00E-27	WP_068082022.1
		Nesiotobacter exalbescens	64%	98%	3.00E-25	WP_028482867.1
		Polymorphum gilvum	64%	98%	2.00E-23	WP_013654598.1
	Rhodopseudomonas palustris BisA53	*Pseudovibrio stylochi*	69%	98%	3.00E-39	WP_068082022.1
		Nesiotobacter exalbescens	69%	98%	3.00E-37	WP_028482867.1
		Labrenzia sp. VG12	65%	98%	2.00E-34	WP_094070879.1
	Pleomorphomonas oryzae DSM 16300^T	*Polymorphum gilvum*	78%	98%	1.00E-40	WP_013654598.1
		Labrenzia aggregata	76%	98%	5.00E-40	WP_006934616.1
		Pannonibacter phragmitetus	76%	98%	2.00E-39	WP_050474405.1
	Cohaesibacter marisflavi CGMCC 1.9157^T	*Pseudovibrio stylochi*	67%	98%	2.00E-35	WP_068082022.1
		Labrenzia	61%	98%	3.00E-33	WP_022998516.1
		Nesiotobacter exalbescens	64%	98%	3.00E-33	WP_028482867.1
	Methylobacterium extorquens DM4^T	*Pseudovibrio stylochi*	67%	98%	2.00E-36	WP_068082022.1
		Polymorphum gilvum	66%	98%	2.00E-35	WP_013654598.1
		Nesiotobacter exalbescens	66%	98%	3.00E-35	WP_028482867.1
	Microvirga massiliensis JC119^T	*Nesiotobacter exalbescens*	68%	98%	3.00E-37	WP_028482867.1
		Pseudovibrio axinellae	68%	98%	2.00E-36	WP_068007089.1
		Polymorphum gilvum	67%	98%	1.00E-34	WP_013654598.1
	Bosea vaviloviae Vaf-18^T	*Nesiotobacter exalbescens*	74%	98%	2.00E-29	WP_028482867.1
		Pseudovibrio hongkongensis	68%	98%	4.00E-29	WP_068316686.1
		Polymorphum gilvum	64%	98%	1.00E-25	WP_013654598.1
	Methyloferula stellata AR4^T	*Nesiotobacter exalbescens*	67%	98%	3.00E-38	WP_028482867.1
		Pseudovibrio axinellae	63%	98%	4.00E-36	WP_068007089.1

		Labrenzia aggregata	61%	98%	1.00E-34	WP_006934616.1
		Pseudovibrio axinellae	74%	98%	1.00E-27	WP_068007089.1
	Methylocella sylvestris BL2^{T}	*Polymorphum gilvum*	68%	98%	7.00E-24	WP_013654598.1
		Pannonibacter phragmitetus	65%	98%	2.00E-22	WP_050474405.1
		Nesiotobacter exalbescens	64%	98%	6.00E-34	WP_028482867.1
	Rhizobium etli CFN42^{T}	*Labrenzia* sp. VG12	63%	98%	4.00E-33	WP_094070879.1
		Polymorphum gilvum	64%	98%	5.00E-33	WP_013654598.1
		Polymorphum gilvum	55%	94%	0	WP_013654610.1
	Bradyrhizobium diazoefficiens USDA 110^{T}	*Pannonibacter phragmitetus*	53%	94%	0	WP_019963540.1
		Labrenzia aggregata	53%	94%	0	WP_055654459.1
		Polymorphum gilvum	54%	99%	4.00E-166	WP_013654610.1
	Rhodopseudomonas palustris BisA53	*Pannonibacter phragmitetus*	53%	99%	8.00E-164	WP_058898120.1
		Labrenzia aggregata	52%	99%	6.00E-163	WP_055654459.1
		Labrenzia sp. VG12	57%	99%	0	WP_094070890.1
	Pleomorphomonas oryzae DSM 16300^{T}	*Polymorphum gilvum*	56%	99%	3.00E-179	WP_013654610.1
		Pannonibacter phragmitetus	56%	99%	6.00E-177	WP_058898120.1
		Pannonibacter phragmitetus	61%	99%	0	WP_058898120.1
fliF	*Cohaesibacter marisflavi* CGMCC 1.9157^{T}	*Polymorphum gilvum*	60%	99%	0	WP_013654610.1
		Labrenzia sp. VG12	59%	99%	0	WP_094070890.1
		Polymorphum gilvum	55%	99%	2.00E-179	WP_013654610.1
	Methylobacterium extorquens DM4^{T}	*Labrenzia aggregata*	54%	99%	8.00E-178	WP_055654459.1
		Pannonibacter phragmitetus	52%	99%	2.00E-166	WP_019963540.1
		Polymorphum gilvum	57%	99%	0	WP_013654610.1
	Microvirga massiliensis JC119^{T}	*Pannonibacter phragmitetus*	56%	99%	0	WP_019963540.1
		Labrenzia aggregata	56%	99%	0	WP_055654459.1
		Labrenzia aggregata	55%	99%	0	WP_055654459.1
	Bosea vaviloviae Vaf-18^{T}	*Pannonibacter phragmitetus*	56%	99%	0	WP_019963540.1
		Polymorphum gilvum	56%	99%	0	WP_013654610.1
	Methyloferula stellata AR4^{T}	*Labrenzia aggregata*	55%	99%	0	WP_055654459.1

		Polymorphum gilvum	57%	99%	2.00E-180	WP_013654610.1
		Pannonibacter phragmitetus	54%	99%	2.00E-167	WP_019963540.1
		Labrenzia alexandrii	56%	99%	0	WP_055673358.1
	Methylocella sylvestris BL2^T	*Polymorphum gilvum*	57%	99%	0	WP_013654610.1
		Pannonibacter phragmitetus	54%	99%	3.00E-176	WP_019963540.1
		Pseudomonas bauzanensis	48%	97%	6.00E-159	EZQ16450.1
	Rhizobium etli CFN42^T	*Nesiotobacter exalbescens*	48%	80%	2.00E-130	WP_028482879.1
		Pseudovibrio stylochi	43%	97%	2.00E-125	WP_068082061.1
		Labrenzia aggregata	74%	91%	3.00E-111	WP_006934633.
	Bradyrhizobium diazoefficiens USDA 110^T	*Polymorphum gilvum*	73%	90%	1.00E-107	WP_013654612.1
		Nesiotobacter exalbescens	72%	91%	3.00E-107	OKL43095.1
		Labrenzia aggregata	72%	98%	3.00E-115	WP_006934633.1
	Rhodopseudomonas palustris BisA53	*Polymorphum gilvum*	75%	91%	5.00E-112	WP_013654612.1
		Nesiotobacter exalbescens	72%	93%	2.00E-110	OKL43095.1
		Pannonibacter phragmitetus	78%	94%	1.00E-120	WP_058898122.1
	Pleomorphomonas oryzae DSM 16300^T	*Labrenzia marina*	77%	93%	8.00E-119	WP_103223635.1
		Polymorphum gilvum	78%	93%	4.00E-118	WP_013654612.1
		Labrenzia marina	74%	91%	1.00E-120	POF30148.1
fliP	*Cohaesibacter marisflavi* CGMCC 1.9157^T	*Pannonibacter phragmitetus*	74%	90%	1.00E-118	WP_058898122.1
		Polymorphum gilvum	72%	91%	9.00E-117	WP_013654612.1
		Labrenzia aggregata	74%	84%	7.00E-112	WP_006934633.1
	Methylobacterium extorquens DM4^T	*Pannonibacter phragmitetus*	74%	84%	2.00E-111	WP_094463440.1
		Polymorphum gilvum	74%	84%	2.00E-110	WP_013654612.1
		Labrenzia aggregata	72%	90%	7.00E-107	WP_006934633.1
	Microvirga massiliensis JC119^T	*Polymorphum gilvum*	71%	91%	2.00E-103	WP_013654612.1
		Stappia sp. 22II-S9-Z10	71%	90%	5.00E-103	ORE95796.1
		Labrenzia aggregata	72%	95%	1.00E-106	WP_006934633.1
	Bosea vaviloviae Vaf-18^T	*Pannonibacter phragmitetus*	71%	94%	9.00E-103	WP_019963542.1
		Polymorphum gilvum	73%	84%	2.00E-102	WP_013654612.1

		Labrenzia aggregata	71%	95%	1.00E-105	WP_006934633.1
	Methyloferula stellata AR4^{T}	*Polymorphum gilvum*	74%	91%	4.00E-102	WP_013654612.1
		Pannonibacter phragmitetus	69%	93%	8.00E-99	WP_058898122.1
		Labrenzia sp. VG12	71%	91%	3.00E-96	WP_094074561.1
	Methylocella sylvestris BL2^{T}	*Polymorphum gilvum*	73%	83%	3.00E-94	WP_013654612.1
		Pannonibacter phragmitetus	73%	83%	4.00E-94	WP_094463440.1
		Pseudomonas bauzanensis	72%	92%	7.00E-112	EZQ16453.1
	Rhizobium etli CFN42^{T}	*Ahrensia marina*	68%	97%	7.00E-110	WP_053999577.1
		Labrenzia marina	65%	99%	2.00E-101	POF30148.1
		Pannonibacter phragmitetus	49%	93%	2.00E-64	WP_026355489.1
	Bradyrhizobium diazoefficiens USDA 110^{T}	*Pseudovibrio* sp. Ab134	52%	88%	5.00E-64	WP_075698969.1
		Labrenzia alexandrii	50%	93%	3.00E-62	WP_008192303.1
		Labrenzia marina	55%	89%	3.00E-64	WP_103223535.1
	Rhodopseudomonas palustris BisA53	*Nesiotobacter exalbescens*	50%	89%	3.00E-63	WP_036490176.1
		Pannonibacter phragmitetus	53%	89%	3.00E-62	WP_026355489.1
		Nesiotobacter exalbescens	50%	93%	4.00E-63	WP_036490176.1
	Pleomorphomonas oryzae DSM 16300^{T}	*Pannonibacter phragmitetus*	52%	93%	1.00E-58	WP_050474389.1
		Labrenzia marina	53%	93%	4.00E-58	WP_103223535.1
		Pannonibacter phragmitetus	55%	97%	3.00E-77	WP_050474389.1
flgH	*Cohaesibacter marisflavi* CGMCC 1.9157^{T}	*Nesiotobacter exalbescens*	55%	93%	2.00E-75	WP_036490176.1
		Labrenzia sp. VG12	54%	97%	2.00E-74	WP_094070893.1
		Pseudovibrio stylochi	52%	93%	1.00E-61	WP_068082075.1
	Methylobacterium extorquens DM4^{T}	*Nesiotobacter exalbescens*	52%	92%	1.00E-60	WP_036490176.1
		Pannonibacter phragmitetus	52%	92%	1.00E-57	WP_026355489.1
		Pseudomonas bauzanensis	52%	99%	2.00E-73	EZQ16455.1
	Microvirga massiliensis JC119^{T}	*Labrenzia marina*	57%	88%	2.00E-70	WP_103223535.1
		Nesiotobacter exalbescens	53%	90%	6.00E-69	WP_036490176.1
	Bosea vaviloviae Vaf-18^{T}	*Polymorphum gilvum*	55%	99%	5.00E-71	WP_013654614.1
		Nesiotobacter exalbescens	53%	95%	1.00E-70	WP_036490176.1

		Labrenzia marina	53%	99%	1.00E-67	WP_103223535.1
		Pannonibacter phragmitetus	50%	99%	3.00E-73	WP_026355489.1
	Methyloferula stellata AR4^T	*Labrenzia aggregata*	53%	99%	1.00E-72	WP_040439910.1
		Stappia aggregata IAM 12614	54%	96%	3.00E-71	EAV44111.1
		Polymorphum gilvum	56%	93%	1.00E-76	WP_013654614.1
	Methylocella sylvestris BL2^T	*Pannonibacter phragmitetus*	53%	97%	1.00E-75	WP_050474389.1
		Labrenzia marina	51%	99%	7.00E-75	WP_103223535.1
		Pseudomonas bauzanensis	50%	92%	2.00E-66	EZQ16455.1
	Rhizobium etli CFN42^T	*Pseudovibrio hongkongensis*	47%	89%	4.00E-62	WP_068317312.1
		Polymorphum gilvum	50%	89%	5.00E-60	WP_013654614.1
		Polymorphum gilvum	64%	94%	7.00E-152	WP_013654616.1
	Bradyrhizobium diazoefficiens USDA 110^T	*Pannonibacter indicus*	65%	94%	8.00E-152	WP_055454342.1
		Labrenzia marina	62%	94%	2.00E-149	WP_103223537.1
		Polymorphum gilvum	70%	93%	1.00E-139	WP_013654616.1
	Rhodopseudomonas palustris BisA53	*Labrenzia alexandrii*	68%	99%	3.00E-136	CTQ75041.1
		Pannonibacter indicus	71%	93%	4.00E-134	WP_055454342.1
		Labrenzia aggregata	68%	89%	1.00E-157	WP_006934650.1
	Pleomorphomonas oryzae DSM 16300^T	*Pannonibacter indicus*	69%	89%	2.00E-154	WP_055454342.1
		Polymorphum gilvum	67%	89%	5.00E-152	WP_013654616.1
flgI		*Polymorphum gilvum*	68%	93%	2.00E-144	WP_013654616.1
	Cohaesibacter marisflavi CGMCC 1.9157^T	*Labrenzia aggregata*	66%	94%	2.00E-143	WP_006934650.1
		Pannonibacter phragmitetus	69%	93%	2.00E-142	WP_050474388.1
		Pannonibacter phragmitetus	68%	91%	5.00E-153	WP_050474388.1
	Methylobacterium extorquens DM4^T	*Labrenzia alexandrii*	65%	94%	3.00E-151	CTQ75041.1
		Polymorphum gilvum	66%	93%	2.00E-150	WP_013654616.1
		Labrenzia alexandrii	63%	99%	6.00E-152	CTQ75041.1
	Microvirga massiliensis JC119^T	*Stappia* sp. 22II-S9-Z10	65%	95%	1.00E-146	WP_083548138.1
		Polymorphum gilvum	67%	93%	2.00E-146	WP_013654616.1
	Bosea vaviloviae Vaf-18^T	*Pannonibacter phragmitetus*	66%	97%	9.00E-148	WP_019963546.1

		Labrenzia alexandrii DFL-11	66%	97%	6.00E-146	EEE44706.1
		Polymorphum gilvum	66%	93%	1.00E-147	WP_013654616.1
		Labrenzia alexandrii	64%	99%	8.00E-148	WP_040451985.1
	Methyloferula stellata AR4^T	*Polymorphum gilvum*	67%	95%	1.00E-143	WP_013654616.1
		Pannonibacter phragmitetus	64%	99%	2.00E-143	WP_019963546.1
		Labrenzia alexandrii DFL-11	62%	98%	1.00E-130	EEE44706.1
	Methylocella sylvestris BL2^T	*Polymorphum gilvum*	64%	94%	6.00E-126	WP_013654616.1
		Pannonibacter phragmitetus	64%	93%	7.00E-126	WP_050474388.1
		Pseudomonas bauzanensis	64%	93%	1.00E-151	EZQ16457.1
	Rhizobium etli CFN42^T	*Acuticoccus yangtzensis*	57%	93%	1.00E-129	WP_075221725.1
		Stappia sp. 22II-S9-Z10	57%	93%	2.00E-129	WP_083548138.1
		Labrenzia alexandrii	66%	99%	9.00E-122	WP_055673361.1
	Bradyrhizobium diazoefficiens USDA 110^T	*Polymorphum gilvum*	68%	99%	6.00E-116	WP_013654618.1
		Pannonibacter phragmitetus	67%	99%	9.00E-116	WP_050474386.1
		Labrenzia sp. OB1	71%	99%	2.00E-130	WP_068417949.1
	Rhodopseudomonas palustris BisA53	*Pannonibacter phragmitetus*	68%	99%	1.00E-122	WP_050474386.1
		Polymorphum gilvum	68%	99%	4.00E-122	WP_013654618.1
		Labrenzia sp. OB1	71%	99%	2.00E-129	WP_068417949.1
	Pleomorphomonas oryzae DSM 16300^T	*Pannonibacter phragmitetus*	72%	99%	2.00E-127	WP_050474386.1
		Polymorphum gilvum	71%	99%	6.00E-125	WP_013654618.1
flgG		*Labrenzia aggregata*	71%	99%	6.00E-135	WP_006934654.1
	Cohaesibacter marisflavi CGMCC 1.9157^T	*Pannonibacter phragmitetus*	73%	99%	1.00E-132	WP_019963548.1
		Polymorphum gilvum	74%	99%	4.00E-132	WP_013654618.1
		Labrenzia sp. VG12	70%	99%	2.00E-133	WP_094070897.1
	Methylobacterium extorquens DM4^T	*Pannonibacter phragmitetus*	68%	99%	2.00E-127	WP_050474386.1
		Polymorphum gilvum	69%	99%	2.00E-127	WP_013654618.1
		Labrenzia alexandrii	68%	99%	2.00E-118	WP_055673361.1
	Microvirga massiliensis JC119^T	*Polymorphum gilvum*	69%	99%	2.00E-116	WP_013654618.1
		Pannonibacter phragmitetus	67%	99%	2.00E-111	WP_050474386.1

	Bosea vaviloviae Vaf-18^T	*Labrenzia sp. VG12*	68%	99%	4.00E-131	WP_094070897.1
		Pannonibacter phragmitetus	69%	99%	1.00E-130	WP_050474386.1
		Polymorphum gilvum	70%	99%	3.00E-130	WP_013654618.1
	Methyloferula stellata AR4^T	*Labrenzia marina*	68%	98%	8.00E-114	WP_103223539.1
		Polymorphum gilvum	66%	98%	2.00E-108	WP_013654618.1
		Pannonibacter phragmitetus	66%	98%	7.00E-108	WP_050474386.1
	Methylocella sylvestris BL2^T	*Labrenzia marina*	65%	99%	2.00E-115	WP_103223539.1
		Pannonibacter phragmitetus	65%	99%	9.00E-112	WP_050474386.1
		Polymorphum gilvum	64%	99%	4.00E-111	WP_013654618.1
	Rhizobium etli CFN42^T	*Pseudomonas bauzanensis*	68%	99%	2.00E-120	EZQ16459.1
		Ahrensia marina	67%	99%	5.00E-113	WP_053999582.1
		Labrenzia alexandrii	62%	99%	2.00E-107	WP_055673361.1
fliE	*Bradyrhizobium diazoefficiens* USDA 110^T	*Labrenzia aggregata*	58%	66%	7.00E-20	WP_006934656.1
		Polymorphum gilvum	57%	62%	6.00E-17	WP_013654619.1
		Pannonibacter phragmitetus	57%	65%	7.00E-17	KND17501.1
	Rhodopseudomonas palustris BisA53	*Labrenzia aggregata*	48%	99%	1.00E-23	WP_077292789.1
		Polymorphum gilvum	52%	99%	2.00E-23	WP_013654619.1
		Pannonibacter phragmitetus	46%	99%	1.00E-19	ALV26355.1
	Pleomorphomonas oryzae DSM 16300^T	*Labrenzia aggregata*	66%	57%	3.00E-20	WP_006934656.1
		Polymorphum gilvum	68%	57%	1.00E-19	WP_013654619.1
		Pannonibacter phragmitetus	66%	57%	6.00E-19	KND17501.1
	Cohaesibacter marisflavi CGMCC 1.9157^T	*Polymorphum gilvum*	50%	99%	2.00E-24	WP_013654619.1
		Labrenzia alexandrii	47%	95%	4.00E-23	WP_008190823.1
		Pannonibacter phragmitetus	47%	99%	6.00E-22	ALV26355.1
	Methylobacterium extorquens DM4^T	*Labrenzia aggregata*	54%	67%	2.00E-21	WP_077292789.1
		Polymorphum gilvum	45%	99%	2.00E-20	WP_013654619.1
		Nesiotobacter exalbescens	46%	98%	5.00E-20	WP_028482887.1
	Microvirga massiliensis JC119^T	*Labrenzia aggregata*	74%	60%	3.00E-26	WP_006934656.1
		Polymorphum gilvum	70%	60%	7.00E-24	WP_013654619.1

		Pannonibacter phragmitetus	71%	60%	9.00E-24	KND17501.1
		Labrenzia sp. VG12	45%	99%	3.00E-06	WP_094070898.1
	Bosea vaviloviae Vaf-18^{T}	*Pannonibacter phragmitetus*	55%	64%	3.00E-04	KND17501.1
		Polymorphum gilvum	47%	99%	0.003	WP_013654619.1
		Labrenzia sp. OB1	59%	64%	1.00E-20	WP_068417941.1
	Methyloferula stellata AR4^{T}	*Polymorphum gilvum*	61%	64%	2.00E-19	WP_013654619.1
		Pannonibacter phragmitetus	51%	72%	6.00E-18	KND17501.1
		Labrenzia aggregata	62%	69%	5.00E-23	WP_077292789.1
	Methylocella sylvestris BL2^{T}	*Polymorphum gilvum*	62%	70%	8.00E-20	WP_013654619.1
		Pannonibacter phragmitetus	58%	68%	3.00E-19	KND17501.1
		Pseudomonas bauzanensis	53%	59%	1.00E-15	EZQ16460.1
	Rhizobium etli CFN42^{T}	*Ahrensia marina*	46%	60%	4.00E-15	WP_053999583.1
		Labrenzia aggregata	47%	61%	8.00E-15	WP_077292789.1
		Pannonibacter phragmitetus	66%	98%	5.00E-64	WP_050474385.1
	Bradyrhizobium diazoefficiens USDA 110^{T}	*Polymorphum gilvum*	64%	98%	1.00E-63	WP_013654620.1
		Labrenzia sp. OB1	64%	99%	3.00E-60	WP_068417937.1
		Pannonibacter phragmitetus	66%	98%	4.00E-58	WP_019963550.1
	Rhodopseudomonas palustris BisA53	*Polymorphum gilvum*	67%	98%	2.00E-57	WP_013654620.1
		Labrenzia sp. OB1	67%	98%	4.00E-56	WP_068417937.1
		Pannonibacter indicus	64%	98%	4.00E-61	WP_055454345.1
	Pleomorphomonas oryzae DSM 16300^{T}	*Labrenzia* sp. OB1	64%	97%	6.00E-58	WP_068417937.1
flgC		*Polymorphum gilvum*	61%	98%	1.00E-57	WP_013654620.1
		Pannonibacter phragmitetus	74%	98%	2.00E-72	WP_019963550.1
	Cohaesibacter marisflavi CGMCC 1.9157^{T}	*Polymorphum gilvum*	69%	98%	2.00E-69	WP_013654620.1
		Labrenzia sp. VG12	71%	99%	3.00E-67	WP_094070899.1
		Polymorphum gilvum	64%	98%	2.00E-52	WP_013654620.1
	Methylobacterium extorquens DM4^{T}	*Pannonibacter indicus*	63%	98%	4.00E-51	WP_055454345.1
		Stappia sp. 22II-S9-Z10	67%	97%	2.00E-50	WP_083548139.1
	Microvirga massiliensis JC119^{T}	*Pannonibacter phragmitetus*	74%	98%	2.00E-69	WP_019963550.1

		Polymorphum gilvum	70%	98%	3.00E-68	WP_013654620.1
		Pseudovibrio axinellae	69%	98%	2.00E-63	WP_068010184.1
		Pannonibacter indicus	69%	98%	3.00E-67	WP_055454345.1
	Bosea vaviloviae Vaf-18^T	*Nesiotobacter exalbescens*	67%	98%	2.00E-64	WP_028482888.1
		Polymorphum gilvum	66%	98%	4.00E-64	WP_013654620.1
		Pannonibacter indicus	63%	97%	4.00E-59	WP_055454345.1
	Methyloferula stellata AR4^T	*Pseudovibrio stylochi*	61%	98%	1.00E-58	WP_068082084.1
		Polymorphum gilvum	60%	97%	5.00E-57	WP_013654620.1
		Pannonibacter indicus	71%	97%	2.00E-67	WP_055454345.1
	Methylocella sylvestris BL2^T	*Polymorphum gilvum*	67%	97%	8.00E-65	WP_013654620.1
		Pseudovibrio stylochi	65%	97%	1.00E-63	WP_068082084.1
		Pseudovibrio stylochi	62%	99%	4.00E-59	WP_068082084.1
	Rhizobium etli CFN42^T	*Inquilinus limosus*	60%	97%	8.00E-57	WP_026869949.1
		Pannonibacter indicus	62%	97%	1.00E-56	WP_055454345.1
		Pannonibacter phragmitetus	48%	91%	2.00E-37	WP_050474384.1
	Bradyrhizobium diazoefficiens USDA 110^T	*Labrenzia* sp. VG12	47%	92%	1.00E-35	WP_094070900.1
		Polymorphum gilvum	47%	91%	2.00E-35	WP_013654621.1
		Pannonibacter phragmitetus	54%	91%	3.00E-41	WP_050474384.1
	Rhodopseudomonas palustris BisA53	*Labrenzia aggregata*	48%	92%	2.00E-38	WP_006934660.1
		Polymorphum gilvum	48%	91%	1.00E-37	WP_013654621.1
		Pseudovibrio hongkongensis	60%	99%	1.00E-44	WP_068316793.1
flgB	*Pleomorphomonas oryzae* DSM 16300^T	*Pannonibacter phragmitetus*	52%	99%	1.00E-40	WP_050474384.1
		Polymorphum gilvum	50%	99%	2.00E-40	WP_013654621.1
		Labrenzia aggregata	57%	98%	4.00E-46	WP_006934660.1
	Cohaesibacter marisflavi CGMCC 1.9157^T	*Pannonibacter indicus*	56%	98%	7.00E-45	WP_055454346.1
		Polymorphum gilvum	54%	98%	2.00E-43	WP_013654621.1
		Polymorphum gilvum	53%	94%	8.00E-39	WP_013654621.1
	Methylobacterium extorquens DM4^T	*Pannonibacter phragmitetus*	50%	94%	2.00E-36	WP_050474384.1
		Labrenzia sp. VG12	48%	94%	4.00E-35	WP_094070900.1

	Microvirga massiliensis JC119T	*Pannonibacter phragmitetus*	62%	95%	1.00E-49	WP_050474384.1
		Polymorphum gilvum	59%	95%	1.00E-48	WP_013654621.1
		Pseudovibrio sp. Tun.PSC04-5.I4	60%	94%	8.00E-46	WP_093179797.1
	Bosea vaviloviae Vaf-18T	*Pannonibacter indicus*	50%	98%	2.00E-31	WP_055454346.1
		Polymorphum gilvum	46%	98%	2.00E-29	WP_013654621.1
		Labrenzia alexandrii	46%	98%	3.00E-29	WP_040451983.1
	Methyloferula stellata AR4T	*Pannonibacter phragmitetus*	50%	94%	8.00E-39	WP_050474384.1
		Polymorphum gilvum	52%	94%	9.00E-39	WP_013654621.1
		Nesiotobacter exalbescens	52%	93%	3.00E-37	WP_028482889.1
	Methylocella sylvestris BL2T	*Polymorphum gilvum*	50%	99%	7.00E-38	WP_013654621.1
		Pseudovibrio sp. Tun.PSC04-5.I4	51%	97%	5.00E-36	WP_093179797.1
		Pannonibacter phragmitetus	47%	99%	8.00E-35	WP_050474384.1
	Rhizobium etli CFN42T	*Pseudomonas bauzanensis*	41%	95%	2.00E-28	EZQ16462.1
		Polymorphum gilvum	40%	98%	6.00E-28	WP_013654621.1
		Nesiotobacter exalbescens	42%	99%	7.00E-28	WP_028482889.1
flhB	*Bradyrhizobium diazoefficiens* USDA 110T	*Pannonibacter phragmitetus*	55%	98%	2.00E-127	WP_019963519.1
		Labrenzia sp. OB1	56%	97%	8.00E-127	WP_068418034.1
		Polymorphum gilvum	58%	97%	2.00E-119	WP_013654587.1
	Rhodopseudomonas palustris BisA53	*Pannonibacter phragmitetus*	59%	93%	3.00E-141	WP_019963519.1
		Labrenzia sp. OB1	55%	94%	1.00E-132	WP_068418034.1
		Brevundimonas diminuta	56%	93%	4.00E-128	OYX21155.1
	Pleomorphomonas oryzae DSM 16300T	*Pannonibacter phragmitetus*	58%	96%	4.00E-151	WP_050474412.1
		Labrenzia sp. OB1	55%	96%	6.00E-137	WP_068418034.1
		Polymorphum gilvum	57%	95%	6.00E-133	WP_013654587.1
	Cohaesibacter marisflavi CGMCC 1.9157T	*Pannonibacter phragmitetus*	56%	98%	2.00E-134	WP_050474412.1
		Labrenzia sp. OB1	57%	96%	1.00E-133	WP_068418034.1
		Polymorphum gilvum	57%	96%	2.00E-122	WP_013654587.1
	Methylobacterium extorquens DM4T	*Brevundimonas diminuta*	55%	98%	3.00E-115	OYX21155.1
		Labrenzia alexandrii	53%	98%	1.00E-111	WP_055670191.1

		Nesiotobacter exalbescens	54%	99%	3.00E-109	WP_028482890.1
	Microvirga massiliensis JC119^{T}	*Brevundimonas diminuta*	60%	99%	5.00E-142	OYX21155.1
		Pannonibacter phragmitetus	57%	98%	4.00E-141	WP_050474412.1
		Labrenzia marina	55%	98%	1.00E-133	WP_103223513.1
	Bosea vaviloviae Vaf-18^{T}	*Pannonibacter phragmitetus*	55%	98%	7.00E-119	WP_019963519.1
		Labrenzia aggregata	51%	99%	1.00E-116	WP_055654437.1
		Polymorphum gilvum	55%	99%	1.00E-108	WP_013654587.1
	Methyloferula stellata AR4^{T}	*Labrenzia* sp. OB1	52%	99%	1.00E-112	WP_068418034.1
		Pannonibacter indicus	55%	99%	8.00E-112	WP_055457260.1
		Polymorphum gilvum	53%	99%	5.00E-105	WP_013654587.1
	Methylocella sylvestris BL2^{T}	*Pannonibacter phragmitetus*	51%	91%	7.00E-111	WP_019963519.1
		Labrenzia sp. VG12	54%	91%	6.00E-110	WP_094070869.1
		Brevundimonas diminuta	51%	91%	2.00E-106	OYX21155.1
	Rhizobium etli CFN42^{T}	*Pannonibacter indicus*	49%	97%	2.00E-88	WP_055457260.1
		Pseudomonas bauzanensis	50%	98%	5.00E-86	EZQ16471.1
		Brevundimonas diminuta	45%	98%	4.00E-82	OYX21155.1
fliG	*Bradyrhizobium diazoefficiens* USDA 110^{T}	*Pannonibacter phragmitetus*	60%	95%	3.00E-125	WP_050474413.1
		Polymorphum gilvum	59%	95%	6.00E-123	WP_013654586.1
		Labrenzia marina	55%	94%	1.00E-111	WP_103223512.1
	Rhodopseudomonas palustris BisA53	*Pannonibacter phragmitetus*	60%	95%	1.00E-146	WP_050474413.1
		Polymorphum gilvum	60%	95%	7.00E-146	WP_013654586.1
		Labrenzia sp. VG12	56%	95%	6.00E-136	WP_094070868.1
	Pleomorphomonas oryzae DSM 16300^{T}	*Pannonibacter phragmitetus*	60%	99%	6.00E-144	WP_019963518.1
		Polymorphum gilvum	61%	95%	2.00E-142	WP_013654586.1
		Labrenzia marina	59%	95%	2.00E-136	WP_103223512.1
	Cohaesibacter marisflavi CGMCC 1.9157^{T}	*Pannonibacter indicus*	65%	94%	1.00E-160	WP_055457261.1
		Polymorphum gilvum	63%	97%	1.00E-157	WP_013654586.1
		Labrenzia sp. OB1	62%	96%	3.00E-155	WP_068418037.1
	Methylobacterium extorquens DM4^{T}	*Polymorphum gilvum*	61%	91%	4.00E-148	WP_013654586.1

	Pannonibacter phragmitetus	58%	92%	2.00E-142	WP_050474413.1
	Labrenzia alexandrii	57%	91%	1.00E-137	WP_050775945.1
	Pannonibacter phragmitetus	57%	97%	4.00E-138	WP_050474413.1
Microvirga massiliensis JC119^T	*Polymorphum gilvum*	58%	97%	2.00E-136	WP_013654586.1
	Labrenzia sp. VG12	56%	96%	3.00E-134	WP_094070868.1
	Polymorphum gilvum	56%	96%	5.00E-124	WP_013654586.1
Bosea vaviloviae Vaf-18^T	*Pannonibacter phragmitetus*	54%	92%	2.00E-121	WP_050474413.1
	Labrenzia sp. OB1	53%	92%	1.00E-117	WP_068418037.1
	Pannonibacter indicus	53%	98%	2.00E-118	WP_055457261.1
Methyloferula stellata AR4^T	*Polymorphum gilvum*	54%	98%	5.00E-117	WP_013654586.1
	Labrenzia sp. VG12	54%	94%	2.00E-114	WP_094070868.1
	Pannonibacter phragmitetus	55%	94%	2.00E-109	WP_050474413.1
Methylocella sylvestris BL2^T	*Polymorphum gilvum*	55%	95%	1.00E-107	WP_013654586.1
	Labrenzia aggregata	53%	98%	8.00E-107	WP_006934600.1
	Pseudomonas bauzanensis	42%	92%	1.00E-78	EZQ16470.1
Rhizobium etli CFN42^T	*Pseudovibrio axinellae*	32%	92%	9.00E-49	WP_068010189.1
	Ahrensia marina	33%	93%	1.00E-46	WP_082376687.1
